# Synthesis of Hybrid Molecules with Imidazole-1,3,4-thiadiazole Core and Evaluation of Biological Activity on *Trypanosoma cruzi* and *Leishmania donovani*

**DOI:** 10.3390/molecules29174125

**Published:** 2024-08-30

**Authors:** Ali Mijoba, Nereida Parra-Giménez, Esteban Fernandez-Moreira, Hegira Ramírez, Xenón Serrano, Zuleima Blanco, Sandra Espinosa, Jaime E. Charris

**Affiliations:** 1 Laboratorio de Síntesis Orgánica, Facultad de Farmacia, Universidad Central de Venezuela, Apartado 47206, Los Chaguaramos, Caracas 1041-A, Venezuela; alimjoba@gmail.com (A.M.); blancomzule@gmail.com (Z.B.); 2 Laboratorio de Fisiología de Parásitos, Centro de Biofísica y Bioquímica, Instituto Venezolano de Investigaciones Científicas (IVIC), Altos de Pipe, Caracas 1020-A, Venezuela; bionereida@gmail.com; 3 Escuela de Medicina, Universidad Espíritu Santo, Guayaquil 092301, Ecuador; estebanfernandez@uees.edu.ec; 4 Dirección de Investigación, Universidad ECOTEC, Km. 13.5 Vía Samborondón, Guayaquil 092302, Ecuador; hramirez@ecotec.edu.ec; 5 Centro de Química Orgánica, Facultad de Ciencias, Universidad Central de Venezuela (UCV), Caracas 1058-A, Venezuela; xenonserrano@gmail.com; 6 Departamento de Química, Universidad Técnica Particular de Loja, Loja 1101608, Ecuador

**Keywords:** nitroimidazole, thiadiazole, hybridization, *T. cruzi*, *L. donovani*

## Abstract

The aim of this work was to obtain and evaluate, as antiprotozoals, new derivatives of benzoate imidazo-1,3,4-thiadiazole **18**–**23** based on the concepts of molecular repositioning and hybridization. In the design of these compounds, two important pharmacophoric subunits of the fexnidazole prototype were used: metronidazole was used as a repositioning molecule, p-aminobenzoic acid was incorporated as a bridge group, and 1,3,4-thiadiazole group was incorporated as a second pharmacophore, which at position 5 has an aromatic group with different substituents incorporated. The final six compounds were obtained through a five-step linear route with moderate to good yields. The biological results demonstrated the potential of this new class of compounds, since three of them **19**–**21** showed inhibitory activity on proliferation, in the order of 50%, in the in vitro assay against epimastigotes of *T. cruzi* (Strain Y sensitive to nifurtimox and benznidazole) and promastigotes of *L. donovani*, at a single concentration of 50 μM.

## 1. Introduction

Millions of people around the world are affected by trypanosomatids; parasites that cause serious illnesses in those who suffer from them. In the Americas, these kinetoplastids cause Chagas disease (CD) (*Trypanosoma cruzi*) and Leishmaniasis caused by over 20 *Leishmania* species. These neglected tropical diseases (NTDs) reduce human potential and they are especially harmful in vulnerable populations [1].

CD or American trypanosomiasis is a neglected tropical disease (NTD) caused by a hemoflagellate protozoan known as *Trypanosoma cruzi* (*T. cruzi*) [1,2,3]. It is endemic in 21 countries, and an estimated 8 million people are infected with *T. cruzi* worldwide, mainly in Latin America, where it remains one of the most serious public health problems, resulting in more than 10,000 deaths per year [4].

It is mostly transmitted when humans come into contact with feces and/or urine of infected blood-sucking triatomine bugs (vector-borne transmission). Other routes of transmission have been identified and include blood transfusion and congenital infections. There are even reported cases of oral infection through ingestion of contaminated foods [1,2]. Because of immigration from South and Central America, hundreds of thousands of people in countries such as Canada and the United States of America, and in much of Europe and some of Africa, the Eastern Mediterranean, and the Western Pacific can also carry the disease [3].

The disease is characterized by two clinical phases. The short acute phase (2 months) is relatively rare, and no specific symptoms are detected, but it can be fatal in children. The chronic phase can remain latent, without symptoms and with low parasitism, for the rest of the patient’s life, or severe symptoms may develop after asymptomatic onset. Approximately 40% of infected individuals progress from the asymptomatic to symptomatic chronic phase, which is mainly characterized by cardiac disorders and up to 10% experience digestive, neurological, or mixed disorders [2,5].

Leishmaniasis caused by various species of the genus *Leishmania*, transmitted by sand flies, currently infects around 12 million people worldwide and is spreading, with ca. 0.7–1 million new cases and around 30,000 deaths annually. The disease comprises three major syndromes: cutaneous, mucocutaneous and visceral leishmaniasis. Dramatically, its visceral form has a 95% fatality rate among the poorest people in the world [6].

**Figure 1:**
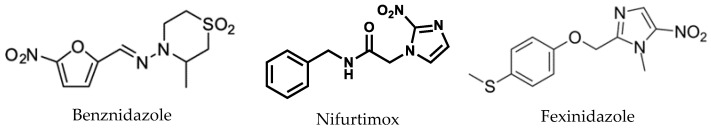
Structures for benznidazole (Bnz), nifurtimox (Nfx), and fexinidazole (Fx).

To date, there is no perspective on an efficacious vaccine against trypanosomiasis or leishmaniasis, the alternative is the development of safe and efficient molecules to treat this disease. Currently, two drugs developed in the 1970s have been clinically used as treatments for this disease: the nitroheterocyclic agents benznidazole (Bnz) and nifurtimox (Nfx) (Figure 1). Both drugs have high antiparasitic efficacy in the acute phase, including the cases of congenital transmission. However, they show low effectiveness and toxicity during the chronic phase and thus a high drop-out rate of patients due to these effects [7,8,9]. Fexinidazole (Fx) represents an important advance in CD drug discovery (Figure 1) [10,11].

These molecules act as prodrugs and are metabolized to exert biological activity. Bnz bioactivation occurs through a series of nonenzymatic reactions producing toxic and highly reactive metabolites capable of modifying lipids, proteins, and *T. cruzi* DNA. Nfx is bioactivated through two sequential reductions of two electrons of the nitro group through type I nitroreductases (TcNTR), resulting in the fragmentation of the heterocyclic ring, producing nitriles that are toxic metabolites to the parasite [12,13].

Despite the great importance of nitroimidazoles as molecules with broad biological activity, the introduction of new congeners has been a challenge to overcome, which has been attributed in the discussions to the high degree of cytotoxicity, genotoxicity, and mutagenicity due to the low selectivity of nitro group bioreduction products, which also affect mammalian cells [14,15]. Several studies have been published where it has been attempted to separate the positive effects of this type of molecule from their toxicity [14,15,16]. Once the low toxicity to the host has been clarified, the use of tools provided by medicinal chemistry is allowed, such as bioisosterism, molecular simplification, and hybridization, to propose new bioactive molecules for this class of structures [17].

**Figure 2:**
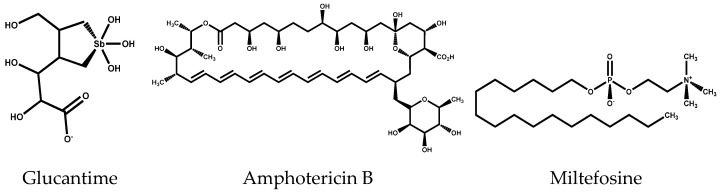
Structures for glucantime, amphotericin B, and miltefosine.

The main chemotherapy for the treatment of leishmaniasis has been based for over 60 years on the use of pentavalent antimonial drugs, meglumine antimoniate (glucantime™) or sodium stibogluconate (pentostam™) being the first-choice drugs, which are highly toxic. Second-line drugs used when patients do not respond to antimonial drugs are amphotericin B and miltefosine. In addition to toxicity, significant drawbacks such as length of treatment, complex route of administration, emergence of drug resistance, and costs limit their use in endemic areas (Figure 2) [18,19,20].

**Figure 3:**
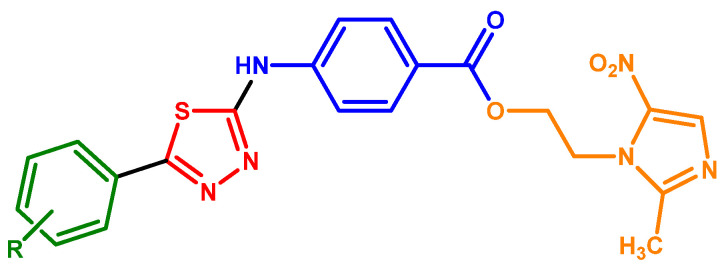
General structures for the benzoates imidazo-1,3,4-thiadiazole **18**–**23**.

**Figure 4:**
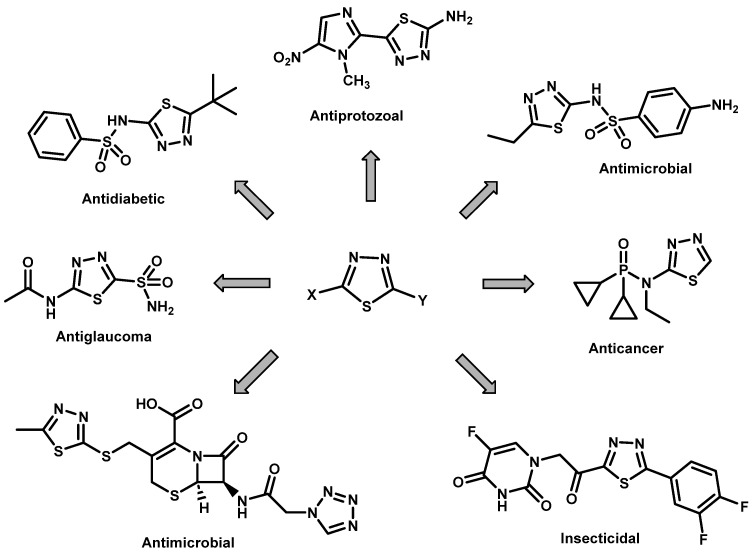
Some active substances placed on the market containing 1,3,4-thiadiazole.

Based on the concepts of repositioning and hybridization, which have proven to be successful for the development of biologically effective agents [21,22,23,24,25], we designed and synthesized the benzoate derivatives imidazo-1,3,4-thiadiazole **1**–**6**, which were also subjected to preliminary evaluation as trypanocides. Metronidazole (Mtz), a heterocycle clinically employed in the treatment of diseases caused by protozoa, was used as a repositioning molecule [26]. The pharmacophoric subunits 5-nitroimidazole were preserved, *p*-aminobenzoic acid was used as a bridge group, and the 1,3,4-thiadiazole group was incorporated as a second pharmacophore (Figure 3). This heterocycle constitutes the central structure of numerous molecules used in medicine, agriculture, and chemical materials (Figure 4) [27,28,29,30]. This time, 1,3,4-thiadiazole at position 5 has an aromatic group with different substituents incorporated.

## 2. Results and Discussion

### 2.1. Synthesis

**Scheme 1:**
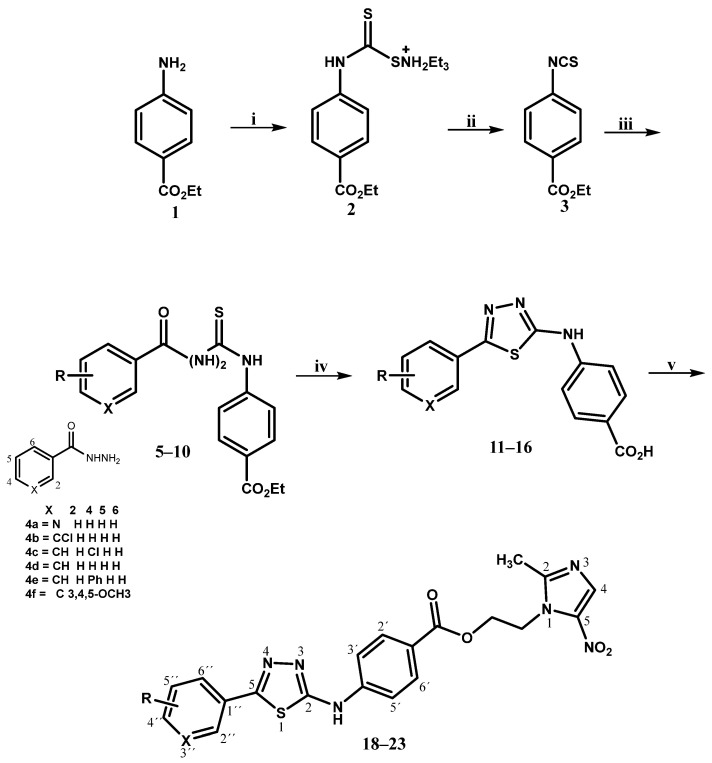
Synthesis of derivatives **18**–**23**. **i.** Et_3_N, CS_2_, 72 h, rt **ii.** ClCO_2_Et, Et_3_N, CHCl_3_, rt **iii. 4a**–**f**, EtOH, 78 °C, 4 h **iv.** a. H_2_SO_4_, 0 °C ‣ rt, 24 h b. LiOH 2N, THF, H_2_O, 80 °C, 24 h **v.** EDCI, DMAP, DMF, Mtz **17**, rt, 48 h.

As shown in Scheme 1, the treatment of ethyl 4-aminobenzoate **1** with carbon disulfide in the presence of triethylamine led to the obtaining of the salt triethylammonium dithiocarbamate **2**, which without prior characterization underwent an elimination reaction using triethylamine, methyl chloroformate, chloroform and room temperature [31]. 4-ethoxycarbonyl isothiocyanate **3** was obtained with excellent yield after purification through chromatographic column [32]. Following the proposed linear synthesis scheme, intermediate **3** was subjected to a reaction with the respective aromatic hydrazides **4a**–**f** in an inert atmosphere to obtain the respective thiosemicarbazides **5**–**10** with a yield between 59–86% [33]. The formation of the 1,3,4-thiadiazole ring was promoted using concentrated sulfuric acid, which constitutes a modification of the methodology described [34], where the intramolecular attack of the sulfur atom on the carbonyl group activated by the strong reaction conditions is facilitated, which allows the obtaining of a mixture of the acid with a minimal portion of the non-hydrolyzed compound. We infer that acid hydrolysis is due to the small amount of water contained in the sulfuric acid used. To ensure complete hydrolysis, the mixture was subjected to a saponification process allowing intermediate compounds **11**–**16** to be obtained with yields above 70% [34]. The final compounds **18**–**23** were generated by a modified version of the Steglich esterification reaction, at room temperature, between acids **11**–**16** with Mtz **17** using *N*-(3-Dimethylamino propyl)-*N*′-ethyl carbodiimide (EDCI) hydrochloride and 4-(Dimethylamino)pyridine (DMAP) in DMF [35].


**No**

**R2″**

**X**

**R4″**

**R5″**

**R6″**

**18**
HNHHH
**19**
HCClHHH
**20**
HCHClHH
**21**
HCHHHH
**22**
HCHPhHH
**23**
HCOCH_3_OCH_3_OCH_3_H

As a model to illustrate the discussion, the compound Benzoate of 2-(2-methyl-5-nitro-1*H*-imidazole-1-yl)-ethyl-4-[5-(4-chlorophenyl)-1,3,4-thiadiazole-2-yl]amino **20** was selected. This compound was obtained with a yield of 75%, as a white solid, soluble in DMSO, presenting a molecular formula C_21_H_17_ClN_6_O_4_S, with a melting point of 202–204 °C. The ^1^H NMR spectrum shows us in the high field a 2.45 ppm singlet, which integrates for 3H, which was assigned to the protons of the methyl group at position 2 of the imidazole, centered at 4.58 and 4.69 ppm. Two coupled triplets are observed, which integrate for 2H each, with coupling constants (*J*) around 5 Hz assigned to the CH_2_O and CH_2_N, respectively, of the ethyl chain attached to position 1 of imidazole, and the signals corresponding to the protons of the aromatic rings present an A′B′ pattern, appearing as centered doublets at 7.54, 7.72, 7.80 and 7.85 ppm with *J* in the order of 8 Hz. The signal corresponding to the proton at position 4 of the imidazole ring appears as a singlet at 8.02 ppm and finally, a brought singlet at 11.1 ppm was assigned to the amino group.

In the ^13^C NMR spectrum, the presence of the condensation product is also confirmed, based on the 17 signals corresponding to the carbon atoms that make up the aforementioned molecule, wherein the high field signals are seen at 14.4, 45.3 and 62.9 ppm assigned to the methyl group at position 2 of imidazole and CH_2_ bound to oxygen and nitrogen, respectively. The signals at 117.3, 128.9, 129.8, 131.1 ppm are assigned to the carbons of the two *p*-substituted aromatic systems and at 133.6 ppm the signal assigned is to the C-4 of the imidazole ring. These unambiguous assignments are based on the results of the DEPT 135° experiment. Other signals that were unequivocally assigned based on the analysis of the HMBC spectrum are 139.1 and 151.9 ppm assigned to imidazole carbons 5 and 2, respectively. The signals at 158.2, 163.9, and 165.3 ppm were assigned to carbons 5 and 2 of the thiadiazole ring and CO group of the ester, respectively. The signals at 122.3, 129.4, 135.5 and 145.1 ppm correspond to the quaternary carbons of the 1,4-substituted aromatic rings. For the unambiguous assignments, it was also necessary to study the analysis of the COSY HMQC, and HMBC spectra (Supplementary Information).

In the IR spectrum, intense bands were observed at 3273 and 3199 cm^−1^, corresponding to the N-H stretch. At 3114–3070 cm^−1^ characteristic bands of stretches =C-H of the aromatic zone can be seen, between 3000 and 2880 cm^−1^ characteristic bands of C-H stretches of the aliphatic zone appear, and at 1711 cm^−1^ there is a typical band of carbonyl carbon C=O of the ester and at 1613, 1480, 1271 cm^−1^ bands corresponding to the aromatic stretches (-C=C-) can be seen, and at 1271 cm^−1^ (C-O-C) (C=C) of the alkoxy groups is present in the molecule. The analytical data for all compounds are summarized in the experimental section.

### 2.2. Antiprotozoal Activity

**Figure 5:**
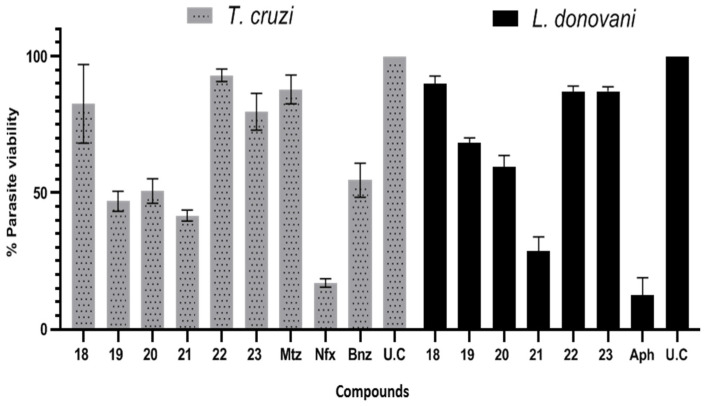
Proliferation of *T. cruzi* epimastigotes and *L. donovani* promastigotes at a concentration of 50 μM for compounds **18**–**23**. Metronidazole, Benznidazole, Amphotericin B and Nifurtimox as treatment control, U.C, untreated control of the parasites.

The in vitro antiproliferative activity of final compounds **18**–**23** was evaluated after 72 h of incubation on epimastigotes of *T. cruzi* [36] and promastigotes of *L. donovani* at a single concentration of 50 μM. A drug-free control (negative control) was included and positive controls were Metronidazole (Mtz), Benznidazole (Bnz), Amphotericin B (Aph), and Nifurtimox (Nfx), (50 μM). The percentage of parasite proliferation inhibition was determined by quantitative metabolic staining with 3-(4,5-dimethyl-2-thiazoyl)-2,5-diphenyltetrazole (MTT) bromide, and the results are described in Figure 5 and Table 1 [37,38].

The results show that the compounds presented antiprotozoal activity against *T. cuzi* and *L. donovani*, with the exception of compounds **18**, **22** and **23** which presented an inhibition percentage of 17.44 ± 14.42, 7.00 ± 2.28, and 20.30 ± 6.73, respectively, on epimastigotes of *T. cruzi*, and compound **19** that showed an inhibition percentage of 31.77 ± 1.95 on promastigotes of *L. donovani.* The rest of the compounds (**19**, **20** and **21**) presented an inhibition percentage on *T. cruzi* epimastigotes in the order of 50%, with IC_50_ = 56.34 μM, 51.70 μM and 55.48 μM, respectively. In addition, it can be observed that these three compounds are more potent than Bnz and four times more powerful than Mtz; however, they are less powerful than Nfx. Relative to the activity against promastigotes of *L. donovani*, compound **21** appeared to be more potent than amphotericin B, with an IC_50_ of 10.07 μM, and an inhibition percentage of 71.42 ± 5.28. The cytotoxicity of all compounds was evaluated on Vero cells by the MTT method and showed low toxicity on mammalian cells (Table 2). From the antiproliferative activities observed, it can be inferred that the hybridization process increased the potency of Mtz, particularly when position 5 of 1,3,4-thiadiazole is occupied by aromatic rings containing low-polarity substituent groups. In this case, with H and halogen atoms such as Cl, polar or bulky groups do not favor the increase in power.

## 3. Materials and Methods

IR spectra were determined using a Perkin-Elmer™ Spectrum two ATR spectrophotometer (Waltham, MA, USA) and are expressed in cm^−1^. The ^1^H and ^13^C NMR spectra were performed using a spectrometer Nanalysis^TM^ 100 MHz PRO Benchtop (Calgary, AB, Canada), JEOL^TM^ Eclipse 270 (Akishima, Japan) or Bruker^TM^ DRX-500 Avance spectrometer (Billerica, MA, USA) (at 100, 270, and 500 MHz for ^1^H and 25.8, 67.9, and 125 MHz for ^13^C) using CDCl_3_ or DMSO-*d*_6_ as the solvents, and are reported in ppm downfield from the residual CHCl_3_ δ 7.25 for ^1^H NMR and 77.0 for ^13^C NMR or DMSO δ 2.54 ppm for ^1^H NMR and 44.5 ppm for ^13^C NMR, respectively. Elemental analyses were achieved using a Perkin Elmer^TM^ 2400 CHN elemental analyzer, and the results were within ±0.4% of the predicted values. Melting points were determined on a Fisher-Johns™ fusiometer (Thermo Fisher Scientific, Waltham, MA, USA) and were not corrected. Thin-layer chromatography (TLC) was carried out on Merck^TM^ silica F254 0.255-mm plates (Darmstadt, Germany), and spots were visualized by UV fluorescence at 254 nm. Chemical reagents were obtained from Aldrich Chemical Co^TM^, St. Louis, MO, USA. All solvents were distilled and dried in the usual manner.

### 3.1. Synthesis of 4-Ethoxycarbonylphenylphenyl Isothiocyanate ***3***

A mixture of 3.75 g (22.7 mmol) of 4-aminobenzoate ethyl **1** in 75 mL of freshly distilled triethylamine was stirred at room temperature (rt) for 15 min, after which 1.5 mL (24.8 mmol) of carbon disulfide was added drop by drop. The mixture was stirred to rt for a time of 24 h, the solid formed was filtered and washed with cold diethyl ether, then it was subjected to vacuum drying at rt for 10 h, producing 6.05 g, 78% of the crude product 4-Ethoxycarbonylphenyltriethyllammonium thiocarbamate **2**, which without prior purification 2.53 g (7.39 mmol) was dissolved in a mixture formed by freshly distilled chloroform 60 mL and 1 mL of triethylamine (7.18 mmol) being subjected to agitation at 0 °C for 30 min. Drop by drop, 0.6 mL (7.75 mmol) of ethyl chloroformate was added. Once the addition was finished, the mixture was allowed to reach rt and the agitation was continued for 24 h, after the reaction the mixture was washed with two portions of HCl 1N 25 mL and then with two portions of a solution saturated with sodium bicarbonate, the organic phase was washed with aqueous solution saturated with NaCl. It was dried in anhydrous magnesium sulfate, filtered, and solvent was removed at reduced pressure. The solid obtained was purified using a chromatographic column using a mixture of hexane: ethyl acetate (97:3) as an eluent, to give a white solid with a yield of 1.29 g, 86%, m.p. 55 °C (Lit. 56–58) [32]. IR (cm^−1^): 3100–3070 (CH_arom_), 3000–2910 (CH_ali_), 2100 (NCS), 1710 (C=O), 1600 (C=C), 1280 (C-O). ^1^H NMR (100 MHz, CDCl_3_) δ ppm: 1,38 (t, 3H, CH_3_, *J* = 7 Hz), 4.40 (q, 2H, CH_2_, *J* = 7 Hz), 7.30 (d, 2H, H_2,6_, *J* = 8 Hz), 8.08 (d, 2H, H_3,5_, *J* = 8 Hz). ^13^C NMR (25.8 MHz, CDCl_3_) δ ppm: 11.8, 58.9, 123.1, 126.6, 128.5, 131.3, 134.9, 162.9. Anal. Calcd for C_10_H_9_NO_2_S: C, 57.96; H, 4.38; N, 6.76; S, 15.47. Found: C, 58.01, H, 4.39; N, 6.93; S, 15.32.

### 3.2. General Procedure for the Synthesis of Thiosemicarbazides ***5***–***10***

To a mixture of intermediate **3**, 0.3 g (1.45 mmol) in 25 mL of dry ethanol, 1 equivalent of the hydrazides **4a**–**f** was added, and the mixture was subjected to reflux with constant agitation for 4 h, after the reaction time was taken to rt, the formed solid was filtered, washed with diethyl ether and dried at 40 °C for 24 h.

*Ethyl 4-(2-nicotinoylhydrazine-1-carbothioamido)benzoate* (**5**). Solid beige, yield: 74%, mp: 200–202 °C. IR (cm^−1^): 3361 (NH), 3181 (CH_arom_), 2979 (CH_ali_), 1710 (C=O), 1656 (C=O), 1601 (C=C), 1366 (NCS), 1273 (C-O). ^1^H NMR (100 MHz, DMSO *d*_6_) δ ppm: 1.35 (t, 3H, CH_3_, *J* = 7 Hz), 4.34 (q, 2H, CH_2_O, *J* = 7 Hz), 7.58 (dd, 1H, H_5″_, *J* = 7 Hz), 7.75 (d, 2H, H_2′,6′_, *J* = 8 Hz), 7.97 (d, 2H, H_3′,5′_, *J* = 8 Hz), 8.32 (d, 1H, H_4″_, *J* = 7 Hz), 8.79 (d, 1H, H_6″_, *J* = 5 Hz), 9.15 (s, 1H, H_2″_), 10.07 (brs, 2H, NH), 10.80 (brs, 1H, NH). ^13^C NMR (25.8 MHz, DMSO *d*_6_) δ ppm: 14.7, 61.1, 123.9, 124.8, 126.4, 128.7, 129.7, 136.0, 144.2, 149.4, 152.9, 165.2, 165.9, 181.8. Calcd for C_16_H_16_N_4_O_3_S: C, 55.80; H, 4.68; N, 16.27; S, 9.31. Found: C, 55.82; H, 4.67; N, 16.35; S, 9.43.

*Ethyl 4-[2-(3-chlorobenzoyl)hydrazine-1-carbothioamido]benzoate* (**6**). White solid, yield: 62%, mp: 188–190 °C. IR (cm^−1^): 3317 (NH), 3160 (CH_arom_), 2981 (CH_ali_), 1718 (C=O), 1634 (C=O), 1610 (C=C), 1352 (NCS), 1282 (C-O). ^1^H NMR (100 MHz, DMSO *d*_6_) δ ppm: 1.36 (t, 3H, CH_3_, *J* = 7 Hz), 4.38 (q, 2H, CH_2_O, *J* = 7 Hz), 7.50–8.05 (m, 8H, Ar), 9.95 (brs, 1H, NH), 10.06 (brs, 1H, NH), 10.72 (brs, 1H, NH). ^13^C NMR (25.8 MHz, DMSO *d*_6_) δ ppm: 14.7, 61.0, 124.6, 127.1, 128.2, 129.7, 130.8, 132.2, 133.6, 135.0, 144.3, 165.2, 181.8. Calcd for C_17_H_16_ClN_3_O_3_S: C, 54.04; H, 4.27; N, 11.12; S, 8.49. Found: C, 53.97, H, 4.32; N, 11.29; S, 8.73.

*Ethyl 4-[2-(4-chlorobenzoyl)hydrazine-1-carbothioamido]benzoate* (**7**). White solid, yield: 59%, mp: 185–186 °C. IR (cm^−1^): 3300, 3211 (NH), 3174 (CH_arom_), 2975 (CH_ali_), 1715 (C=O), 1612 (C=C), 1338 (NCS), 1277 (C-O). ^1^H NMR (100 MHz, DMSO *d*_6_) δ ppm: 1.28 (t, 3H, CH_3_, *J* = 7 Hz), 4.27 (q, 2H, CH_2_O, *J* = 7 Hz), 7.57 (d, 2H, H_2′,6′_, *J* = 8 Hz), 7.68 (brs, 2H, H_3″,5″_), 7.89 (d, 2H, H_3′,5′_, *J* = 8 Hz), 7.94 (d, 2H, H_2″,6″_, *J* = 8 Hz), 9.93 (brs, 1H, NH), 10.02 (brs, 1H, NH), 10.67 (brs, 1H, NH). ^13^C NMR(125 MHz, DMSO *d*_6_) δ ppm: 14.7, 61.1, 125.6, 126.6, 128.9, 129.6, 130.3, 131.7, 137.3, 144.2, 165.9, 180.2. Calcd for C_17_H_16_ClN_3_O_3_S: C, 54.04; H, 4.27; N, 11.12; S, 8.49. Found: C, 54.11, H, 4.29; N, 11.35; S, 8.61.

*Ethyl 4-(2-benzoylhydrazine-1-carbothioamido)benzoate* (**8**). White solid, yield: 81%, mp: 176–178 °C. IR (cm^−1^): 3300 (NH), 3211 (NH), 3174 (CH_arom_), 2975 (CH_ali_), 1698 (C=O), 1604 (C=C), 1338 (CNS), 1280 (C-O). ^1^H NMR (500 MHz, DMSO *d*_6_) δ ppm: 1.39 (t, 3H, CH_3_, *J* = 7 Hz), 4.36 (q, 2H, CH_2_O, *J* = 7 Hz), 7.53 (t, 1H, H_4″_, *J* = 8 Hz), 7.62–7.64 (m, 4H, Ar), 7.86 (d, 2H, H_3′,5′_, *J* = 8 Hz), 8.00–8.02 (m, 2H, Ar), 9.21 (brs, 1H, NH), 10.24 (brs, 1H, NH), 10.67 (brs, 1H, NH). ^13^C NMR (125 MHz, DMSO *d*_6_) δ ppm: 14.7, 61.0, 125.5, 126.6, 128.4, 128.8, 129.5, 132.4, 144.3, 165.9, 180.7. Calcd for C_17_H_17_N_3_O_3_S: C, 59.46; H, 4.99; N, 12.24; S, 9.34. Found: C, 59.53, H, 5.10; N, 12.41; S, 9.57.

*Ethyl 4-{2-[(1,1′-biphenyl)-4-carbonyl]hydrazine-1-carbothioamido}benzoate* (**9**). White solid, yield: 78%, mp: 190–192 °C. IR (cm^−1^): 3307 (NH), 3254 (NH), 3152 (CH_arom_), 2981 (CH_ali_), 1693 (C=O), 1659 (C=O), 1603 (C=C), 1284 (C-O). ^1^H NMR (500 MHz, DMSO *d*_6_) δ ppm: 1.25 (t, 3H, CH_3_, *J* = 7 Hz), 4.23 (q, 2H, CH_2_O, *J* = 7 Hz), 7.37 (t, 1H, _4′′′_, *J* = 8 Hz), 7.45 (t, 2H, H_3′′′, 5′′′_, *J* = 8 Hz), 7.66 (d, 2H, H_2′′′_, _6′′′_), 7.74 (d, 4H, H_2″,6″_,_2′,6′_, *J* = 8 Hz), 7.87 (d, 2H, Ar), 7.97 (d, 2H, H_3′_,_5′_, *J* = 8 Hz), 9.84 (brs, 1H, NH), 9.99 (brs, 1H, NH), 10.62 (brs, 1H, NH). ^13^C NMR (125 MHz, DMSO *d*_6_) δ ppm: 14.5, 61.4, 127.0, 128.8, 129.6, 139.3, 144.1, 166.2, 180.1. Calcd for C_23_H_21_N_3_O_3_S: C, 65.85; H, 5.05; N, 10.02; S, 7.64. Found: C, 65.91, H, 5.07; N, 9.87; S, 7.85.

*Ethyl 4-[2-(3,4,5-trimethoxybenzoyl)hydrazine-1-carbothioamido]benzoate* (**10**). White solid, yield: 86%, mp: 196–198 °C. IR (cm^−1^): 3314 (NH), 3277 (NH), 3161 (CH_arom_), 2984 (CH_ali_), 1728 (C=O), 1663 (C=C), 1610 (C=C), 1281 (C-O). ^1^H NMR (100 MHz, DMSO *d*_6_) δ ppm: 1.35 (t, 3H, CH_3_, *J* = 7 Hz), 3.77 (s, 3H, OCH_3_), 3.88 (s, 6H, OCH_3_), 4.33 (c, 2H, CH_2_O, *J* = 7 Hz), 7.33 (s, 2H, H_2″,6″_,), 7.74 (d, 2H, H_2′,6′_, *J* = 8 Hz), 7.95 (d, 2H, H_3′,5′_, *J* = 8 Hz), 10.02 (brs, 2H, NH), 10.54 (brs, 1H, NH). ^13^C NMR (25.8 MHz, DMSO *d*_6_) δ ppm: 14.7, 56.7, 60.8, 61.0, 106.2, 124.5, 127.9, 129.7, 144.3, 153.1, 165.8, 180.7. Calcd for C_20_H_23_N_3_O_6_S: C, 55.42; H, 5.35; N, 9.69; S, 7.40. Found: C, 55.29, H, 5.37; N, 9.93; S, 7.61.

### 3.3. General Procedure for the Synthesis of 4-[(1,3,4-Thiadiazol-2-yl)amino]benzoic Acid Derivatives ***11***–***16***

To one equivalent of thiosemicarbazide **5**–**10** in an ice bath was added drop to drop 5 mL of cold concentrated H_2_SO_4_, after the addition the ice bath was removed, allowing it to react to rt and with constant agitation for 24 h. After the reaction time, ice was added to the mixture, and the solid obtained was filtered by suction and washed with small portions of distilled water and diethyl ether, then dried in a vacuum oven at 40 °C for 24 h. The solid obtained was dissolved in 5 mL of tetrahydrofuran (THF), then 5 mL of a 2N LiOH solution was added, and the mixture was refluxed under constant agitation at 80 °C for 24 h. The solution obtained was washed 3 times with aliquots of 10 mL of CHCl_3_, discarding the organic phase in each case. The aqueous phase was acidified with 20% HCl to a pH of 4–5. The solid obtained corresponding to the acid thiadiazoles was filtered by suction and then dried in a vacuum oven, at 40 °C for 24 h.

*4-{[5-(Pyridin-3-yl)-1,3,4-thiadiazol-2-yl]amino}benzoic Acid* (**11**). White solid, purification by recrystallization in ethanol–water, yield: 87%, mp: >300 °C. IR (cm^−1^): 3252 (NH), 3187 (NH), 3056 (CH_arom_), 2741 (OH), 1681 (C=O), 1601 (C=C), 1557 (C=N), 1418 (OH), 1268 (C-O). ^1^H NMR (500 MHz, DMSO *d*_6_) δ ppm: 7.55 (t, 1H, H_5″_, *J* = 5 Hz), 7.72 (d, 2H, H_3′,5′_, *J* = 8 Hz), 7.92 (d, 2H, H_2′,6′_, *J* = 8 Hz), 8.23 (d, 1H, H_4″_, *J* = 7 Hz), 8.65 (brs, 1H, H_6″_), 9.02 (brs, 1H, H_2″_), 10.99 (brs, 1H, COOH). ^13^C NMR (125 MHz, DMSO *d*_6_) δ ppm: 117.3, 124.9, 131.3, 133.6, 134.8, 139.1, 147.7, 151.5, 156.5, 161.1, 167.4. Calcd for C_14_H_10_N_4_O_2_S: C, 56.37; H, 3.38; N, 18.78; S, 10.75. Found: C, 56.31, H, 3.39; N, 18.95; S, 10.67.

*4-{[5-(3-Chlorophenyl)-1,3,4-thiadiazol-2-yl]amino}benzoic Acid* (**12**). Light yellow solid, purification by recrystallization in ethanol–water, yield: 75%, mp: 232–234 °C. IR (cm^−1^): 3255 (NH), 3187 (NH), 3056 (CH_arom_), 2981 (OH), 1674 (C=O), 1605 (C=C), 1553 (C=N), 1422 (OH), 1293 (C-O). ^1^H NMR (500 MHz, DMSO *d*_6_) δ ppm: 7.50–755 (m, 2H, H_4″_,_6″_), 7.75 (d, 2H, H_3′,5′_, *J* = 8 Hz), 7.79 (t, 1H, H_5″_, *J* = 8 Hz), 7.88 (s, 1H, H_2″_), 7.93 (d, 2H, H_2′,6′_, *J* = 8 Hz), 11.11 (brs, 1H, COOH). ^13^C NMR (125 MHz, DMSO *d*_6_) δ ppm: 117.3, 124.4, 126.2, 126.5, 130.6, 131.3, 131.7, 132.5, 134.4, 144.5, 157.6, 164.4, 167.4. Calcd for C_15_H_10_ClN_3_O_2_S: C, 54.30; H, 3.04; N, 12.67; S, 9.66. Found: C, 54.38, H, 2.98; N, 12.63; S, 9.78.

*4-{[5-(4-Chlorophenyl)-1,3,4-thiadiazol-2-yl]amino}benzoic Acid* (**13**). White solid, purification by recrystallization in ethanol–water, yield: 71%, mp: > 300 °C. IR (cm^−1^): 3248 (NH), 3187 (NH), 3174 (CH_arom_), 2920 (OH), 1640 (C=O), 1560 (C=N), 1480 (C=C), 1240 (C-O). ^1^H NMR (270 MHz, DMSO *d*_6_) δ ppm: 7.59 (d, 2H, Ar), 7.76 (d, 2H, H_3′,5′_, *J* = 8 Hz), 7.90 (d, 2H, Ar, *J* = 8 Hz), 7.97 (d, 2H, H_2′,6′_, *J* = 8 Hz), 10.98 (brs, 1H, NH), 12.70 (brs, 1H, COOH). ^13^C NMR (67.9 MHz, DMSO *d*_6_) δ ppm: 117.3, 124.4, 129.1, 129.5, 129.9, 131.4, 135.5, 144.6, 158.2, 164.2, 167.5. Calcd for C_15_H_10_ClN_3_O_2_S: C, 54.30; H, 3.04; N, 12.67; S, 9.66. Found: C, 54.27, H, 3.09; N, 12.81; S, 9.93.

*4-[(5-Phenyl-1,3,4-thiadiazol-2-yl)amino]benzoic Acid* (**14**). White crystalline solid, purification by recrystallization in ethanol–water, yield: 95%, mp: >300 °C. IR (cm^−1^): 3281 (NH), 3207 (NH), 3089 (CH_arom_), 2981 (OH), 1689 (C=O), 1611 (C=C), 1501 (OH), 1249 (C-O). ^1^H NMR (270 MHz, DMSO *d*_6_) δ ppm: 7.50–7.52 (m, 3H, Ar), 7.78 (d, 2H, H_2′,6′_, *J* = 8 Hz), 7.88–7.89 (m, 2H, Ar), 7.96 (d, 2H, H_3′,5′_, *J* = 8 Hz), 10.98 (brs, 1H, NH), 12.29 (brs, 1H, COOH). ^13^C NMR (67.9 MHz, DMSO *d*_6_) δ ppm: 117.3, 124.5, 127.5, 129.9, 130.7, 131.0, 131.4, 144.8, 159.2, 163.9, 167.5. Calcd for C_15_H_11_N_3_O_2_S: C, 60.59; H, 3.73; N, 14.13; S, 10.78. Found: C, 60.64, H, 3.73; N, 14.47; S, 10.87.

*4-{[5-((1,1’-Biphenyl)-4-yl)-1,3,4-thiadiazol-2-yl]amino}benzoic Acid* (**15**). White crystalline solid, purification by recrystallization in ethanol–water, yield: 84%, mp: >300 °C. IR (cm^−1^): 3281 (NH), 3207 (NH), 3089 (CH_arom_), 2981 (OH), 1689 (C=O), 1611 (C=C), 1501 (OH), 1249 (C-O). ^1^H NMR (500 MHz, DMSO *d*_6_) δ ppm: 7.68–7.72 (m, 5H, Ar), 7.75 (d, 2H, H_3′,5′_, *J* = 8 Hz), 7.82 (d, 2H, H_3″,5″_, *J* = 8 Hz), 7.93–7.95 (m, 4H, Ar), 10.90 (brs, 1H, COOH). ^13^C NMR (125 MHz, DMSO *d*_6_) δ ppm: 117.2, 126.5, 126.8, 127.9, 128.0, 129.7, 131.3, 139.4, 141.9, 148.5, 158.7, 163.9, 167.6. Calcd for C_21_H_15_N_3_O_2_S: C, 67.54; H, 4.05; N, 11.25; S, 8.59. Found: C, 67.43, H, 3.97; N, 11.43; S, 8.71.

*4-{[5-(3,4,5-Trimethoxyphenyl)-1,3,4-thiadiazol-2-yl]amino}benzoic Acid* (**16**). Brown solid, purification by recrystallization in ethanol–water, yield: 91%, mp: >300 °C. IR (cm^−1^): 3249 (NH), 3199 (NH), 3048 (CH_arom_), 2982 (OH), 1687 (C=O), 1601 (C=C), 1415 (OH), 1286 (C-O). ^1^H NMR (500 MHz, DMSO *d*_6_) δ ppm: 3.67 (s, 3H, OCH_3_), 3.79 (s, 3H, OCH_3_), 3.81 (s, 3H, OCH_3_), 6.09–7.06 (m, 3H, Ar), 7.75 (d, 2H, H_2′,6′_, *J* = 8 Hz), 11.54 (brs, 1H, COOH). ^13^C NMR (125 MHz, DMSO *d*_6_) δ ppm: 56.5, 56.6, 60.9, 104.8, 117.1, 120.8, 123.8, 126.1, 131.2, 138.4, 144.8, 148.6, 153.7, 159.7, 163.7, 167.6. Calcd for C_18_H_17_N_3_O_5_S, C, 55.81; H, 4.42; N, 10.85; S, 8.28. Found: C, 55.78, H, 4.49; N, 11.09; S, 8.53.

### 3.4. General Procedure for the Synthesis of 2-(2-Methyl-5-nitro-1H-imidazol-1-yl)ethyl 4-[(5-phenyl-1,3,4-thiadiazol-2-yl)amino]benzoate Derivatives ***18***–***23***

A mixture of 1.0 equivalent of the corresponding acid **11**–**16** in 5 mL of dry dimethylformamide (DMF) was stirred to rt for 10 min, then 1-Ethyl-3-(3-dimethylaminopropyl)carbodiimide hydrochloride (EDCI) (1.5 equivalents) was added. The resulting mixture was stirred at 0 °C for 30 min, then 4-dimethylamine pyridine (DMAP) 5% and metronidazole **17** (1 equivalent) dissolved in 2 mL of dry DMF were added. The reaction mixture was left under an inert atmosphere at rt and constant agitation for 24 h. After this time, the mixture was washed with three portions of 20 mL of 10% NaHCO_3_, then with water and aqueous solution saturated with NaCl, dried on anhydrous sodium sulfate, filtered, the organic phase was eliminated at reduced pressure. The solid formed was washed with diethyl ether, and drying was carried out at reduced pressure at 40 °C for 24 h.

*2-(2-Methyl-5-nitro-1H-imidazol-1-yl)ethyl-4-[(5-(pyridin-3-yl)-1,3,4-thiadiazol-2-yl]amino)benzoate* (**18**). Beige solid, recrystallization from ethanol–water, yield: 55%, mp: 232–234 °C. IR (cm^−1^): 3273 (NH), 3199 (NH), 3057 (CH_arom_), 3000–2800 (CH_ali_), 1715 (C=O), 1610 (C=C), 1491 (C=C), 1270 (C-O). ^1^H NMR (500 MHz, DMSO *d*_6_) δ ppm: 2.45 (s, 3H, CH_3_), 4.59 (t, 2H, CH_2_O, *J* = 5 Hz), 4.70 (t, 2H, CH_2_N, *J* = 5 Hz), 7.54 (m, 1H, H_5”_), 7.74 (d, 2H, H_3′,5′_, *J* = 9 Hz), 7.84 (d, 2H, H_2′,6′_, *J* = 9 Hz), 8.03 (s, 1H, H_3_), 8.25 (m, 1H, H_4”_), 8.67 (s, 1H, H_2”_), 9.05 (m, 1H, H_6″_), 11.06 (brs, 1H, NH). ^13^C NMR (125 MHz, DMSO *d*_6_) δ ppm: 14.4, 45.3, 62.9, 117.4, 122.4, 131.1, 133.6, 134.7, 145.1, 147.9, 151.6, 156.5, 164.3, 165.3. Calcd for C_20_H_17_N_7_O_4_S: C, 53.21; H, 3.80; N, 21.72; S, 7.10. Found: C, 53.32, H, 3.86; N, 21.84; S, 6.91.

*2-(2-Methyl-5-nitro-1H-imidazol-1-yl)ethyl-4-{[5-(3-chlorophenyl)-1,3,4-thiadiazol-2-yl]amino}benzoate* (**19**). White solid, recrystallization from ethanol–water, yield: 40%, mp: 202–204 °C. IR (cm^−1^): 3327 (NH), 3199 (NH), 3140–3070 (CH_arom_), 3000–2800 (CH_ali_), 1690 (C=O), 1604 (C=C), 1496 (C=C), 1276 (C-O). ^1^H NMR (500 MHz, DMSO *d*_6_) δ ppm: 2.48 (s, 3H, CH_3_), 4.62 (t, 2H, CH_2_O, *J* = 5 Hz), 4.73 (t, 2H, CH_2_N, *J* = 5 Hz), 7.43 (m, 1H, H_5”_), 7.47 (d, 2H, H_3′,5′_, *J* = 8 Hz), 7.48 (m, 1H, H_4″_), 7.80 (d, 2H, H_2′,6′_, *J* = 8 Hz), 7.91 (m, 2H, H4, H_6″_), 7.95 (s, 1H, H_2″_), 11.01 (brs, 1H, NH). ^13^C NMR (125 MHz, DMSO *d*_6_) δ ppm: 14.7, 43.7, 62.3, 116.7, 120.1, 134.9, 129.0, 131.7, 132.9, 134.7, 134.8, 138.4, 144.0, 151.7, 152.7, 165.9, 174.1. Calcd for C_21_H_17_ClN_6_O_4_S: C, 52.02; H, 3.53; N, 17.33; S, 6.61. Found: C, 51.93, H, 3.61; N, 17.62; S, 6.87.

*2-(2-Methyl-5-nitro-1H-imidazol-1-yl)ethyl-4-{[5-(4-chlorophenyl)-1,3,4-thiadiazol-yl]amino}benzoate* (**20**). Beige solid, recrystallization from ethanol–water, yield: 54%, mp: 242–244 °C. IR (cm^−1^): 3273 (NH), 3199 (NH), 3114–3070 (CH_arom_), 3000–2800 (CH_ali_), 1711 (C=O), 1613 (C=C), 1480 (C=C), 1271 (C-O). ^1^H NMR (500 MHz, DMSO *d*_6_) δ ppm: 2.45 (s, 3H, CH_3_), 4.58 (t, 2H, CH_2_O, *J* = 5 Hz), 4.69 (t, 2H, CH_2_N, *J* = 5 Hz), 7.54 (d, 2H, H_3”,5”_, *J* = 8,5 Hz), 7.72 (d, 2H, H_3′,5′_, *J* = 8.5 Hz), 7.80 (d, 2H, H_2′,6′_, *J* = 8.5 Hz), 7.85 (d, 2H, H_2”,6”_, *J* = 8.5 Hz), 8.02 (s, 1H, H_4_), 11.1 (brs, 1H, NH). ^13^C NMR (125 MHz, DMSO *d*_6_) δ ppm: 14.4, 45.3, 62.9, 117.3, 122.3, 128.9, 129.4, 129.8, 131.1, 133.6, 135.5, 139.1, 145.1, 151.9, 158.2, 163.9, 165.3. Calcd for C_21_H_17_ClN_6_O_4_S: C, 52.02; H, 3.53; N, 17.33; S, 6.61. Found: C, 52.17, H, 3.54; N, 17.29; S, 6.94.

*2-(2-Methyl-5-nitro-1H-imidazol-1-yl)ethyl-4-[(5-phenyl-1,3,4-thiadiazol-2-yl)amino]benzoate* (**21**). Beige solid, recrystallization from ethanol–water, yield: 60%, mp: 240–242 °C. IR (cm^−1^): 3277 (NH), 3200 (NH), 3120–3072 (CH_arom_), 2970 (CH_ali_), 1702 (C=O), 1611 (C=C), 1492 (C=C), 1286 (C-O). ^1^H NMR (500 MHz, DMSO *d*_6_) δ ppm: 2.46 (s, 3H, CH_3_), 4.60 (t, 2H, CH_2_O, *J* = 5 Hz), 4.71 (t, 2H, CH_2_N, *J* = 5 Hz), 7.49–7.58 (m, 2H, Ar), 7.74 (d, 2H, H_3′,5′_, *J* = 8.5 Hz), 7.83 (d, 2H, H_2′,6′_, *J* = 8.5 Hz), 7.85–7.87 (m, 3H, Ar), 10.92 (brs, 1H, NH). ^13^C NMR (125 MHz, DMSO *d*_6_) δ ppm: 14.4, 45.3, 62.9, 117.3, 122.3, 127.4, 129.8, 129.9, 130.6, 130.9, 131.1, 133.6, 145.3, 159.4, 163.8, 165.4. Calcd for C_21_H_18_N_6_O_4_S: C, 55.99; H, 4.03; N, 18.66; S, 7.12. Found: C, 56.075, H, 4.01; N, 18.83; S, 7.37.

*2-(2-Methyl-5-nitro-1H-imidazol-1-yl)ethyl-4-{[5-((1,1’-biphenyl)-4-yl)-1,3,4-thiadiazol-2-yl]amino}benzoate* (**22**). Light yellow solid, recrystallization from ethanol–water, yield: 60%, mp: >300 °C. IR (cm^−1^): 3327 (NH), 3199 (NH), 3140 (CH_arom_), 3000–2800 (CH_ali_), 1690 (C=O), 1604 (C=C), 1496 (C=C), 1276 (C-O). ^1^H NMR (500 MHz, DMSO *d*_6_) δ ppm: 2.49 (s, 3H, CH_3_), 4.61 (t, 2H, CH_2_O, *J* = 4.5 Hz), 4.74 (t, 2H, CH_2_N, *J* = 4.5 Hz), 7.70 (m, 5H, H Ar), 7.84 (d, 2H, H_3′,5′_, *J* = 8.5 Hz), 7.88 (2d, 4H, H Ar), 7.93 (d, 2H, H_2’,6’_, *J* = 8.5 Hz), 8.05 (s, 1H, H_4_), 11.04 (brs, 1H, NH). ^13^C NMR (125 MHz, DMSO *d*_6_) δ ppm: 14.3, 44.7, 62.4, 117.5, 121.8, 127.6, 127.9, 129.2, 129.9, 130.7, 132.9, 138.4, 140.6, 145.3, 151.4, 152.1, 163.4, 165.8. Calcd for C_27_H_22_N_6_O_4_S: C, 61.59; H, 4.21; N, 15.96; S, 6.09. Found: C, 61.75, H, 3.97; N, 15.83; S, 7.28.

*2-(2-Methyl-5-nitro-1H-imidazol-1-yl)ethyl-4-{[5-(3,4,5-trimethoxyphenyl)-1,3,4-thiadiazol-2-yl]amino}benzoate* (**23**). Yellow solid, recrystallization from ethanol–water, yield: 66%, mp: 252 °C. IR (cm^−1^): 3278 (NH), 3192 (NH), 3061 (CH_arom_), 3000–2820 (CH_ali_), 1731 (C=O), 1604 (C=C), 1496 (C=C), 1267 (C-O). ^1^H NMR (500 MHz, DMSO *d*_6_) δ ppm: 2.48 (s, 3H, CH_3_), 3.70 (s, 3H, OCH_3_), 3.84 (s, 6H, OCH_3_), 4.59 (t, 2H, CH_2_O, *J* = 5 Hz), 4.70 (t, 2H, CH_2_N, *J* = 5 Hz), 7.12 (s, 2H, H_6”_), 7.39 (d, 2H, H_3’,5’_, *J* = 8 Hz), 7.74 (d, 2H, H_2’,6’_, *J* = 8 Hz), 8.11 (s, 1H, H_4_), 11.06 (brs, 1H, NH). ^13^C NMR (125 MHz, DMSO *d*_6_) δ ppm: 14.3, 45.2, 56.1, 60.8, 62.3, 105.3, 117.8, 122.1, 127.3, 130.7, 132.2, 138.4, 139.2, 144.8, 147.4, 152.7, 153.4, 164.5, 165.7. Calcd for C_24_H_24_N_6_O_7_S: C, 53.33; H, 4.48; N, 15.55; S, 5.93. Found: C, 53.37, H, 4.52; N, 15.71; S, 6.25.

### 3.5. Biology

#### 3.5.1. Biological Material

*Trypanosoma cruzi* epimastigotes (Y strain (TCII)) were used [36], with culture in axenic conditions in liver infusion tryptose medium (LIT) supplemented with 10% inactive fetal bovine serum, by weekly ringing at 28 °C. Parasites were collected in the logarithmic phase of growth before the experiments.

*Leishmania donovani* promastigotes (LD51 strain) were grown in liver infusion tryptose medium (LIT), with 100% inactive fetal bovine serum. Parasites were collected in the logarithmic phase of growth before the experiments.

#### 3.5.2. In Vitro Evaluation of the Effect of Derivatives **17**–**22** on the Proliferation of Epimastigotes of *Trypanosoma cruzi*: MTT Test

It was arranged in plates of 96 wells, 2 × 10^5^ epimastigotes per well and incubated at 28 °C for 24 h. Subsequently, the compounds to be evaluated, dissolved in DMSO (0.1% final), were added at a single concentration of 50 μM. A drug-free control (negative control) was included, and benznidazole and nifurtimox were included as positive controls. To select those compounds active on *T. cruzi* epimastigotes, it was incubated for 72 h after the compounds to be evaluated were added and then MTT (5 mg/mL), dissolved in Phosphate buffered saline (PBS), was added and incubated for 4 h in the dark. Finally, the cells were lysed with DMSO and the plate was read in a spectrophotometer at 570 nm [37,38].

#### 3.5.3. In Vitro Evaluation of the Effect of Derivatives **17**–**22** on the Proliferation of Promastigotes of *Leishmania donovani*: MTT Test

It was arranged in plates of 96 wells, 4 × 10^5^ promastigotes per well and incubated at room temperature °C for 24 h. Subsequently, the compounds to be evaluated, dissolved in DMSO (0.1% final), were added at a single concentration of 50 μM, a drug-free control (negative control) was included, and amphotericin B was used as leishmanicidal control. To select those compounds active on promastigotes, it was incubated for 72 h after and then MTT (5 mg/mL), dissolved in PBS, was added and incubated for 4 h in the dark. Finally, the cells were lysed with DMSO and the plate was read in a spectrophotometer at 570 nm [37].

#### 3.5.4. Estimation of Half Maximal Inhibitory Concentration (IC_50_) of Derivatives **19**, **20**, and **21** on Epimastigotes of *T. cruzi* and Promastigotes of *L. donovani*: MTT Test

The test compounds, as well as the reference drugs, metronidazole, nifurtimox and amphotericin B, were previously dissolved in DMSO and then in LIT medium and added to the cultures at the required concentrations; the final DMSO concentration in cultures was 1% (*v*/*v*). Eight concentrations of each compound in the range 5–500 µM were tested and assayed in triplicate. Growth inhibition was assessed after 72 h incubation in the presence of the compounds by MTT (5 mg/mL), dissolved in PBS, and incubated for 4 h in the dark. Finally, the parasites were lysed with DMSO and the plate was read in a spectrophotometer at 570 nm.

#### 3.5.5. Host Cell Toxicity Assay

To determine the possible toxic effects of the compounds on the host cells, uninfected Vero cells, maintained in DMEM, BSF 10%, incubated at 37 °C in humidified 5% CO_2_, were counted in suspension in a Neubauer chamber and seeded at 2 × 10^4^ cells/well in a 96-well plate. After 24 h compounds were added. The viability of the cells was measured at 48 h using MTT [3-(4,5-Dimethyl-2-thiazolyl)-2,5-diphenyl-2*H*-tetrazolium bromide] colorimetric assay. A total of 5 mg/mL of MTT was added and incubated in darkness for 4 h. After this time, DMSO was added and the plate was read at 540 nm in a spectrophotometer Synergy HT (Biotek, Winooski, VT, USA). The test was carried out in triplicate in different concentrations: 5, 15, 25, 50, 100, 200 and 300 μM, including untreated cells and reference drug controls.

## 4. Conclusions

In summary, we present the synthesis of six molecules in which the nuclei of 5-nitroimidazole and thiadiazole were integrated, making use of the hybridization concept widely used in medicinal chemistry. Each final compound was obtained with moderate yields through a synthesis strategy that was very useful and feasible. Different spectroscopic tools were used for its identification and characterization. As trypanocidal, only three compounds showed activity as inhibitors of the proliferation of *T. cruzi* epimastigotes, compounds **19**–**21** with an activity of 53.10 ± 3.68, 49.37 ± 4.45, and 58.35 ± 2.05%, respectively, compared to the activity shown by the reference compound Bnz 45.39 ± 6.21 and Mtz 12.18 ± 5.30; however, they are less potent than Nfx 83.00 ± 1.60%. Regarding leishmanicidal activity, only compound 21 showed a more moderate activity than Aph with an IC_50_ of 10.07 compared to 0.33 ± 0.02 presented by Aph. The compounds appear to have a little cytotoxic effect on Vero cells when compared to the value presented by Bnz and Nfx against these mammalian cells. From the antiproliferative activities observed, it can be inferred that the hybridization process increased the potency of Mtz against *T. cruzi* and *L. donovani*, particularly when position 5 of 1,3,4-thiadiazole is occupied by aromatic rings containing low-polarity substituent groups. More detailed studies are required to confirm the quality of derivatives as a new class of antiparasitic agents.

## Figures and Tables

**Table 1:** Antiproliferative activity (%) of compounds **18**–**23**, Mtz, Aph, Nfx, and Bnz (50μM) after 72 h of incubation with *T. cruzi* epimastigotes and *L. donovani* promastigotes, and IC_50_ μM for compounds more actives.

	*T. cruzi*	*L. donovani*	IC_50_ μM *T. cruzi*	IC_50_ μM *L. donovani*
No	% Inhibition ± SD			
**18**	17.44 ± 14.42	10.08 ± 2.92	nd	nd
**19**	53.10 ± 3.68	31.77 ± 1.95	56.34 ± 6.83	59.72 ± 8.82
**20**	49.37 ± 4.45	40.38 ± 4.18	51.70 ± 6.59	54.48 ± 8.51
**21**	58.35 ± 2.05	71.42 ± 5.28	55.48 ± 7.20	10.07 ± 2.21
**22**	7.00 ± 2.28	12.95 ± 2.08	nd	nd
**23**	20.30 ± 6.73	12.74 ± 2.31	nd	nd
**Mtz**	12.18 ± 5.30	--	180.20 ± 18.9	50.31 ± 11.9
**Bnz**	45.39 ± 6.21	--	nd	--
**Nfx**	83.00 ± 1.60	--	1.81 ± 0.45	--
**Aph**	--	62.44 ± 6.60	--	0.33 ± 0.02

SD: Standard deviation. nd: Not determined.

**Table 2:** Cytotoxicity on Vero cells of the hybrid molecules with imidazole-1,3,4-thiadiazole core.

No	CC_50_ (μM)	*T. cruzi* (SI)	*L. donovani* (SI)
**19**	>500	>8.87	>8.37
**20**	>500	>9.67	>9.17
**21**	>500	>9.01	>49.65
**Nfx**	>90	48.65	--
**Aph**	>250	--	757.58

SI: Selectivity index.

## Data Availability

Data is contained within the article and Supplementary Materials.

## Supplementary Materials

The following supporting information can be downloaded at: https://www.mdpi.com/article/10.3390/molecules29174125/s1, The following are available: ^1^H/^13^C NMR, DEPT 135°, COSY, HMQC, and HMBC for compounds **3**, **7**–**9**, **11**, **15**, **18**, **20**–**22**.

## References

[1] World Health Organization (2020). Ending the Neglect to Attain the Sustainable Development Goals: A Road Map for Neglected Tropical Diseases 2021–2030.

[2] World Health Organization http://www.who.int/chagas/en/.

[3] Castillo-Riquelme M. (2017). Chagas disease in non-endemic countries. Lancet Glob. Health.

[4] (2021). World Health Organization|Epidemiology, WHO. http://www.who.int/chagas/epidemiology/en/.

[5] Coura J.R., Borges-Pereira J. (2010). Chagas disease: 100 years after its discovery. A systemic review. Acta Tropica..

[6] Burza S., Croft S.L., Boelaert M. (2018). Leishmaniasis. Lancet.

[7] Santos-Cruz L.F., Ramírez-Cruz B.G., García-Salomé M., Olvera-Romero Z.Y., Hernández-Luis F., Hernández-Portilla L.B., Durán-Díaz Á., Dueñas-García I.E., Castañeda-Partida L., Piedra-Ibarra E. (2020). Genotoxicity assessment of four novel quinazoline-derived trypanocidal agents in the Drosophila wing somatic mutation and recombination test. Mutagenesis.

[8] (2020). Symptoms, Transmission, and Current Treatments for Chagas Disease DNDi. https://dndi.org/diseases/chagas/facts/.

[9] Ribeiro V., Dias N., Paiva T., Hagström-Bex L., Nitz N., Pratesi R., Hecht M. (2020). Current trends in the pharmacological management of Chagas disease. Int. J. Parasitol. Drugs Drug Resi..

[10] (2020). Fexinidazole for Chagas DNDi. https://dndi.org/research-development/portfolio/fexinidazole-chagas/.

[11] Torrico F., Gascón J., Ortiz L., Pinto J., Rojas G., Palacios A., Barreira F., Blum B., Schijman A.G., Vaillant M. (2023). A Phase 2, randomized, multicenter, placebo-controlled, proof-of-concept trial of oral fexinidazole in adults with chronic indeterminate Chagas Disease. Clin. Infect. Dis..

[12] Do Vale Chaves e Mello F., Castro Salomão Quaresma B.M., Resende Pitombeira M.C., Araújo de Brito M., Farias P.P., Lisboa de Castro S., Salomão K., Silva de Carvalho A., Oliveira de Paula J.I., de Brito Nascimento S. (2020). Novel nitroimidazole derivatives evaluated for their trypanocidal, cytotoxic, and genotoxic activities. Eur. J. Med. Chem..

[13] Patterson S., Wyllie S. (2014). Nitro drugs for the treatment of trypanosomatid diseases: Past, present, and future prospects. Trends Parasitol..

[14] Boechat N., Carvalho A.S., Salomão K., de Castro S.L., Araujo-Lima C.F., Mello F.V., Felzenszwalb I., Aiub C.A., Conde T.R., Zamith H.P. (2015). Studies of genotoxicity and mutagenicity of nitroimidazoles: Demystifying this critical relationship with the nitro group. Mem. Inst. Oswaldo Cruz..

[15] Mello F.V.C., Carvalho A.S., Bastos M.M., Boechat N., Aiub C.A.F., Felzenszwalb I. (2013). Evaluation of genotoxic effects of new molecules with possible trypanocidal activity for Chagas disease treatment. Sci. World J..

[16] De Carvalho A.S., Salomão K., de Castro S.L., Conde T.R., da Silva Zamith H.P., Caffarena E.R., Hall B.S., Wilkinson S.R., Boechat N. (2014). Megazol and its bioisostere 4H-1,2,4-triazole: Comparing the trypanocidal, cytotoxic and genotoxic activities and their in vitro and in silico interactions with the *Trypanosoma brucei* nitroreductase enzyme. Mem. Inst. Oswaldo Cruz.

[17] Barreiro E.J. (2009). A Química Medicinal e o paradigma do composto-protótipo. Rev. Virtual Quim..

[18] Zauli-Nascimento R.C., Miguel D.C., Yokoyama-Yasunaka J.K., Pereira L.I., Pelli de Oliveira M.A., Ribeiro-Dias F., Dorta M.L., Uliana S.R. (2010). In vitro sensitivity of Leishmania (Viannia) braziliensis and Leishmania (Leishmania) amazonensis Brazilian isolates to meglumine antimoniate and amphotericin B. Trop. Med. Int. Health.

[19] Chakravarty J., Sundar S. (2010). Drug resistance in leishmaniasis. J. Glob. Infect. dis..

[20] Savoia D. (2015). Recent updates and perspectives on leishmaniasis. J. Infect. Dev. Ctries.

[21] Fortin S., Bérubé G. (2013). Advances in the development of hybrid anticancer drugs. Expert Opin. Drug Discov..

[22] Morphy R., Rankovic Z. (2005). Designed multiple ligands. An emerging drug discovery paradigm. J. Med. Chem..

[23] Pawełczyk A., Sowa-Kasprzak K., Olender D., Zaprutko L. (2018). Molecular consortia—Various structural and synthetic concepts for more effective therapeutics synthesis. Int. J. Mol. Sci..

[24] Sampath H.M., Herrmann L., Tsogoeva S.B. (2020). Structural hybridization as a facile approach to new drug candidates. Bioorg. Med. Chem. Lett..

[25] Soltan O.M., Shoman M.F., Abdel-Aziz S.A., Narumi A., Konno H., Abdel-Aziz M. (2021). Molecular hybrids: A five-year survey on structures of multiple targeted hybrids of protein kinase inhibitors for cancer therapy. Eur. J. Med. Chem..

[26] Leitsch D. (2019). A review on metronidazole: An old warhorse in antimicrobial chemotherapy. Parasitology.

[27] Kumar D., Kumar H., Kumar V., Deep A., Sharma A., Marwaha M.G., Marwaha R.K. (2023). Mechanism-based approaches of 1,3,4-thiadiazole scaffolds as potent enzyme inhibitors for cytotoxicity and antiviral activity. Med. Drug Dis..

[28] Rossi R., Ciofalo M. (2020). An updated review on the synthesis and antibacterial activity of molecular hybrids and conjugates bearing imidazole moiety. Molecules.

[29] Hu Y., Li C.-Y., Wang X.-M., Yang Y.-H., Zhu H.-L. (2014). 1,3,4-Thiadiazole: Synthesis, reactions, and applications in medicinal, agricultural, and materials chemistry. Chem. Rev..

[30] Atmaram U.A., Roopan S.M. (2022). Biological activity of oxadiazole and thiadiazole derivatives. Appl. Microbiol. Biotechnol..

[31] Segawa J., Kitano M., Kazuno K., Matsuoka M., Shirahase I., Ozaki M., Matsuda M., Tomii Y., Kise M. (1992). Studies on pyridonecarboxylic acids. 1. Synthesis and antibacterial evaluation of 7-substituted-6-halo-4-oxo-4H[1,3]thiazeto[3,2-a]quinoline-3-carboxylic acids. J. Med. Chem..

[32] Vinkler E., Klinényi F., Stájer G., Ferenczy L. (1967). Contibutions to the synthesis of mustard oil carbonic acid esters of antimicronial action. Acta Pharm. Hung..

[33] Rollas S., Karakuş S., Barlas–Durgun B., Kiraz M., Erdeniz H. (1996). Synthesis and antimicrobial activity of some 1,4-disubstituted thiosemicarbazide and 2,5-disubstituted-1,3,4-thiadiazole derivatives. Farmaco.

[34] Hoggarth E. (1949). Compounds related to thiosemicarbazide. Part II. 1-Benzoylthiosemicarbazides. J. Chem. Soc..

[35] Neises B., Steglich W. (1978). Simple method for the esterification of carboxylic acids. Angew. Chem. Int. Engl..

[36] Zingales B., Andrade S.G., Briones M.R.S., Campbell D.A., Chiari E., Fernandes O., Guhl F., Lages-Silva E., Macedo A.M., Machado C.R. (2009). A new consensus for Trypanosoma cruzi intraspecific nomenclature: Second revision meeting recommends TcI to TcVI. Mem. Inst. Oswaldo Cruz.

[37] Mosmann T. (1983). Rapid colorimetric assay for cellular growth and survival: Application to proliferation and cytotoxicity assays. J. Immunol. Methods.

[38] Mijoba A., Fernandez-Moreira E., Parra-Giménez N., Espinosa-Tapia S., Blanco Z., Ramírez H., Charris J.E. (2023). Synthesis of Benzocycloalkanone-based Michael Acceptors and Biological Activities as Antimalarial and Antitrypanosomal Agents. Molecules.

