# Supplementary material for: Synthesis of Hybrid Molecules with Imidazole-1,3,4-thiadiazole Core and Evaluation of Biological Activity on Trypanosoma cruzi and Leishmania donovani
Source: Molecules. 2024 Aug 30;29(17):4125. doi: 10.3390/molecules29174125 (PMC11396972; doi:10.3390/molecules29174125)
Supplement: Supplementary file 1 [file molecules-29-04125-s001.zip › molecules-3152701-supplementary.pdf]

Supporting Information for

Synthesis of Hybrid Molecules with Imidazole-1,3,4-thiadiazole Core and Evaluation of Biological Activity on *Trypanosoma cruzi* and L*eishmania donovani*

Ali Mijoba [1,2], Nereida Parra-Giménez [2], Esteban Fernandez-Moreira [3], Hegira Ramírez [4], Xenón Serrano [5], Zuleima Blanco [1], Sandra Espinosa [6,*] and Jaime E. Charris [1,*]

1 Laboratorio de Síntesis Orgánica, Facultad de Farmacia, Universidad Central de Venezuela, Apartado 47206, Los Chaguaramos, Caracas 1041-A, Venezuela

2 Laboratorio de Fisiología de Parásitos, Centro de Biofísica y Bioquímica, Instituto Venezolano de Investigaciones Científicas (IVIC), Altos de Pipe, Caracas 1020-A, Venezuela

3 Escuela de Medicina, Universidad Espíritu Santo, Guayaquil 092301, Ecuador

4 Dirección de Investigación, Universidad ECOTEC, Km. 13.5 Vía Samborondón, Guayaquil 092302, Ecuador

5 Centro de Química Orgánica, Facultad de Ciencias, Universidad Central de Venezuela (UCV), Caracas 1058-A, Venezuela

6 Departamento de Química, Universidad Técnica Particular de Loja, Loja 1101608, Ecuador

Table of Contents

		Page
**S1**	The ^{1}H NMR spectrum of **3**	4
**S2**	The ^{13}C NMR spectrum of **3**	5
**S3**	The DEPT 135º spectrum **3**	5
**S4**	The ^{1}H NMR spectrum of **11**	6
**S5**	The DEPT 135º spectrum of **11**	7
**S6**	The COSY spectrum of **11**	7
**S7**	The HMQC spectrum of **11**	8
**S8**	The ^{1}H NMR spectrum of **18**	9
**S9**	The ^{13}C NMR spectrum of **18**	10
**S10**	The DEPT 135º spectrum of **18**	10
**S11**	The HMQC spectrum of **18**	11
**S12**	The HMBC spectrum of **18**	11
**S13**	The ^{1}H NMR spectrum of **7**	12
**S14**	The ^{13}C NMR spectrum of **7**	13
**S15**	The DEPT 135º spectrum of **7**	13
**S16**	The COSY spectrum of **7**	14
**S17**	The HMQC spectrum of **7**	14
**S18**	The HMBC spectrum of **7**	15
**S19**	The ^{1}H NMR spectrum of **20**	16
**S20**	The ^{13}C NMR spectrum of **20**	17
**S21**	The DEPT 135º spectrum of **20**	17
**S22**	The COSY spectrum of **20**	18
**S23**	The HMQC spectrum of **20**	18
**S24**	The HMBC spectrum of **20**	19
**S25**	The ^{1}H NMR spectrum of **8**	20
**S26**	The ^{13}C NMR spectrum of **8**	21
**S27**	The DEPT 135º spectrum of **8**	21
**S28**	The COSY spectrum of **8**	22
**S29**	The HMQC spectrum of **8**	22
**S30**	The HMBC spectrum of **8**	23
**S31**	The ^{1}H NMR spectrum of **21**	24
**S32**	The ^{13}C NMR spectrum of **21**	25
**S33**	The DEPT 135º spectrum of **21**	25
**S34**	The COSY spectrum of **21**	26
**S35**	The HMQC spectrum of **21**	26
**S36**	The HMBC spectrum of **21**	27
**S37**	The ^{1}H NMR spectrum of **9**	28
**S38**	The ^{13}C NMR spectrum of **9**	29
**S39**	The DEPT 135º spectrum of **9**	29
**S40**	The COSY spectrum of **9**	30
**S41**	The HMQC spectrum of **9**	30
**S42**	The HMBC spectrum of **9**	31
**S43**	The ^{1}H NMR spectrum of **15**	32
**S44**	The ^{13}C NMR spectrum of **15**	33
**S45**	The DEPT 135º spectrum of **15**	33

**S46**	The COSY spectrum of **15**	34
**S47**	The HMQC spectrum of **15**	34
**S48**	The HMBC spectrum of **15**	35
**S49**	The ^{1}H NMR spectrum of **22**	36
**S50**	The ^{13}C NMR spectrum of **22**	37
**S51**	The DEPT 135º spectrum of **22**	37
**S52**	The COSY spectrum of **22**	38
**S53**	The HMQC spectrum of **22**	38
**S54**	The HMBC spectrum of **22**	39

S1

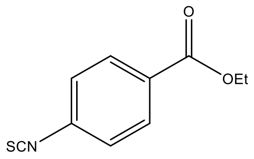
O
OEt
SCN

3

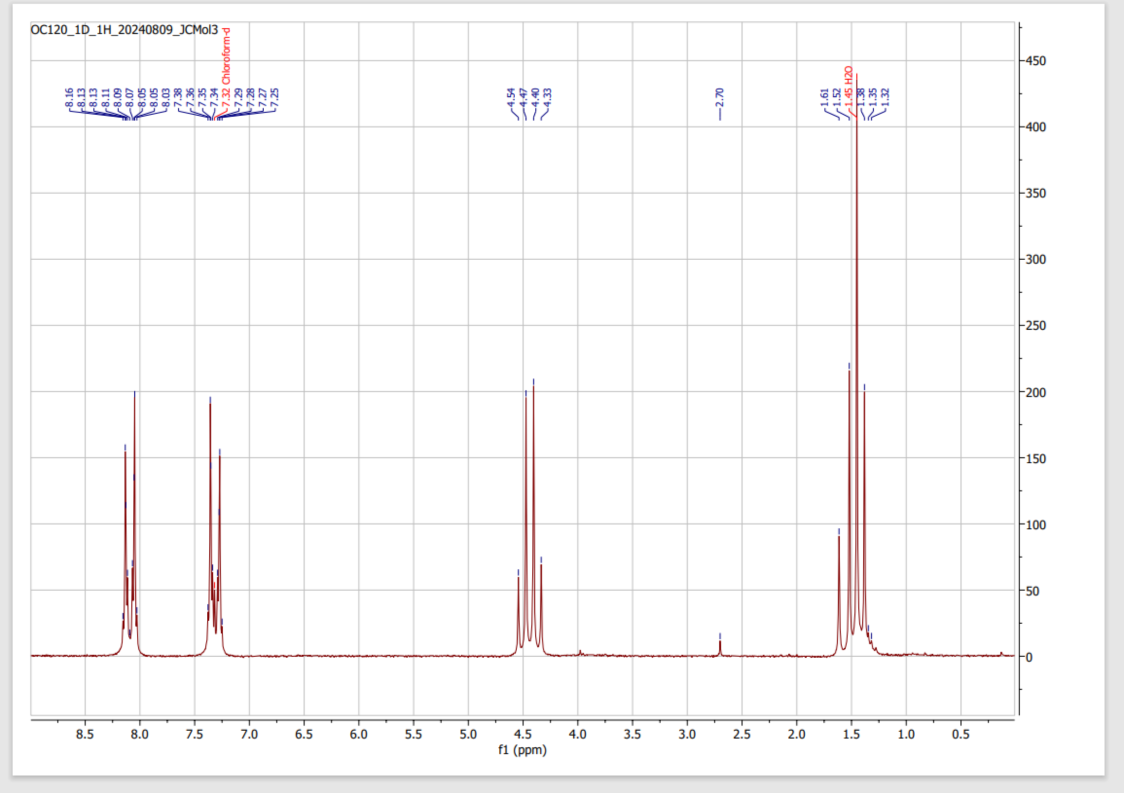
OC120_1D_1H_20240809_JCMol3
8.16
8.13
8.13
8.11
8.09
8.07
8.05
8.05
8.03
7.38
7.36
7.35
7.34
7.32 Chloroform-d
7.29
7.28
7.27
7.25
4.54
4.47
4.40
4.33
2.70
1.61
1.52
1.45 H2O
1.38
1.35
1.32
450
400
350
300
250
200
150
100
50
0
8.5
8.0
7.5
7.0
6.5
6.0
5.5
5.0
4.5
4.0
3.5
3.0
2.5
2.0
1.5
1.0
0.5
f1 (ppm)

S2

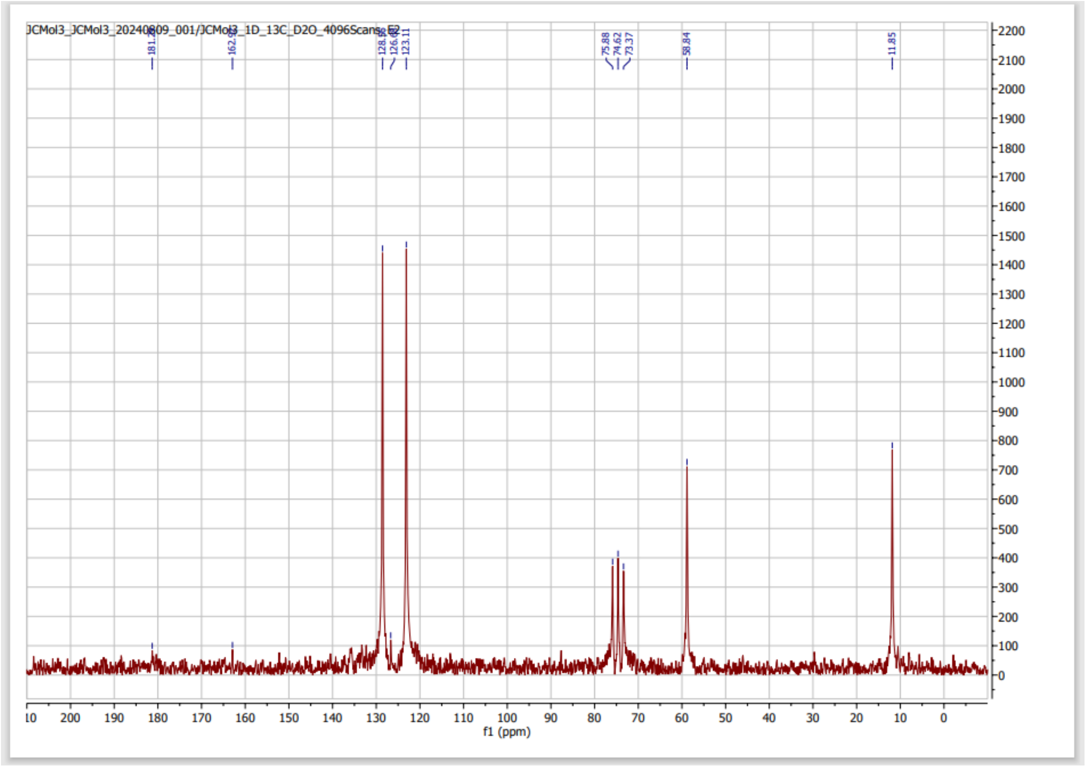
JCMol3_JCMol3_20240809_001/JCMol3_1D_13C_D2O_4096Scans_E2
123.11
75.88
74.62
73.37
58.84
11.85
f1 (ppm)

S3

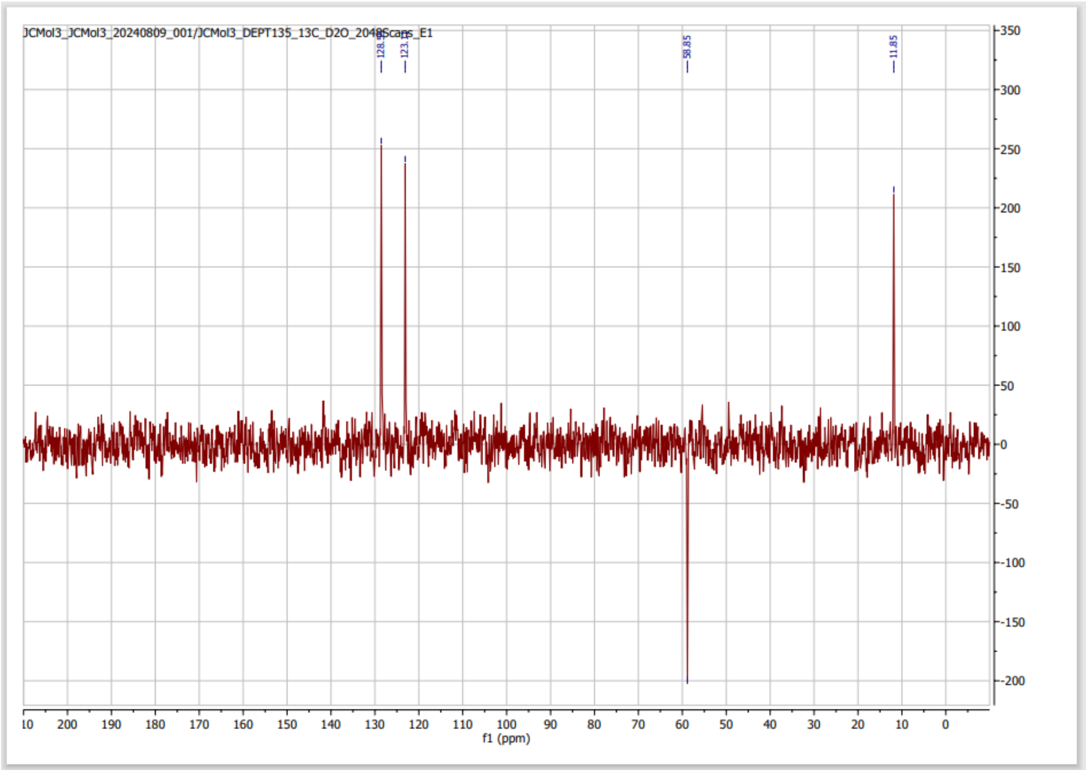
JCMol3_JCMol3_20240809_001/JCMol3_DEPT135_13C_D2O_2048Scans_E1
58.85
11.85
f1 (ppm)

S4

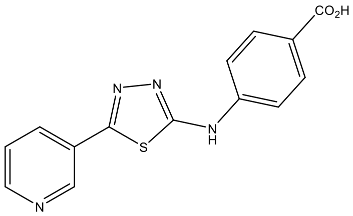

11

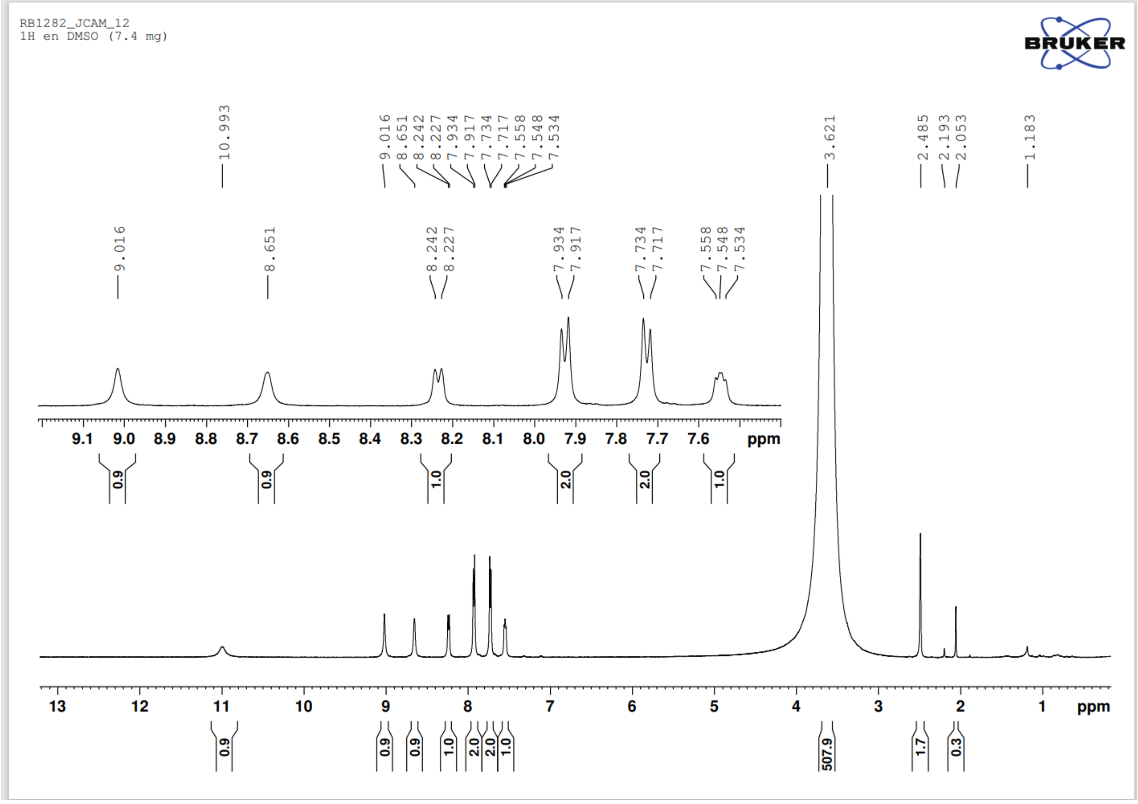

S5

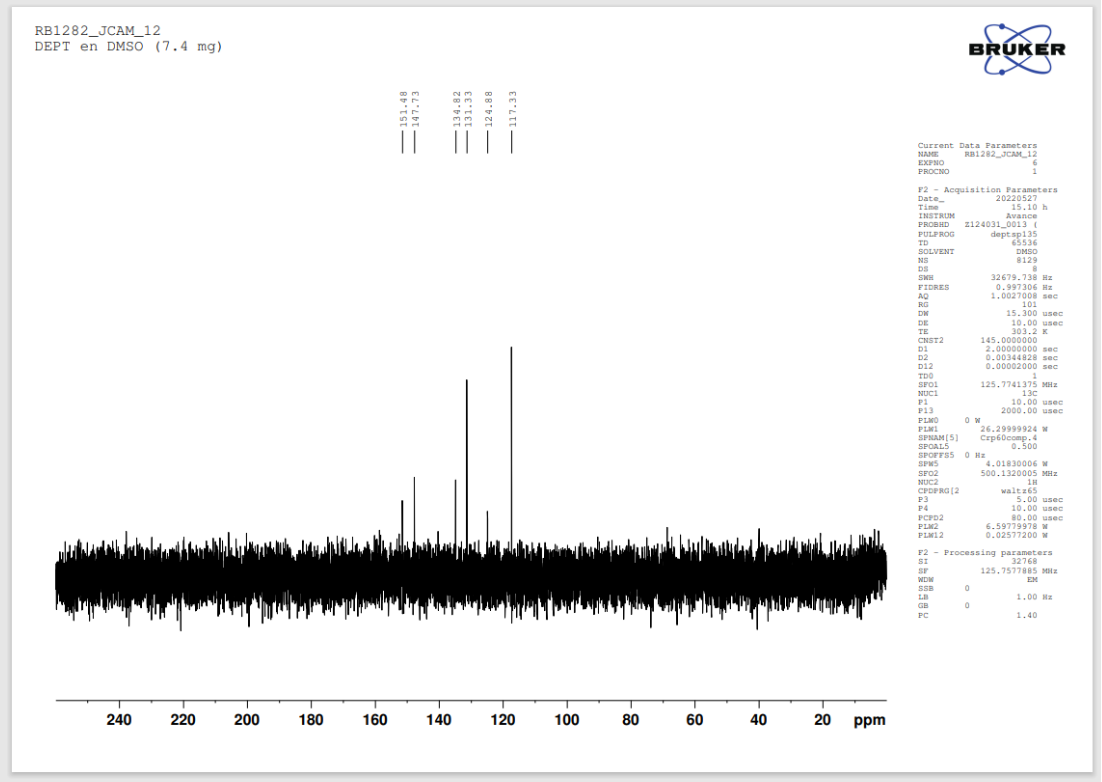

RB1282_JCAM_12
DEPT en DMSO (7.4 mg)
151.48
147.73
134.82
131.33
124.88
117.33
BRUKER
Current Data Parameters
240 220 200 180 160 140 120 100 80 60 40 20 ppm

S6

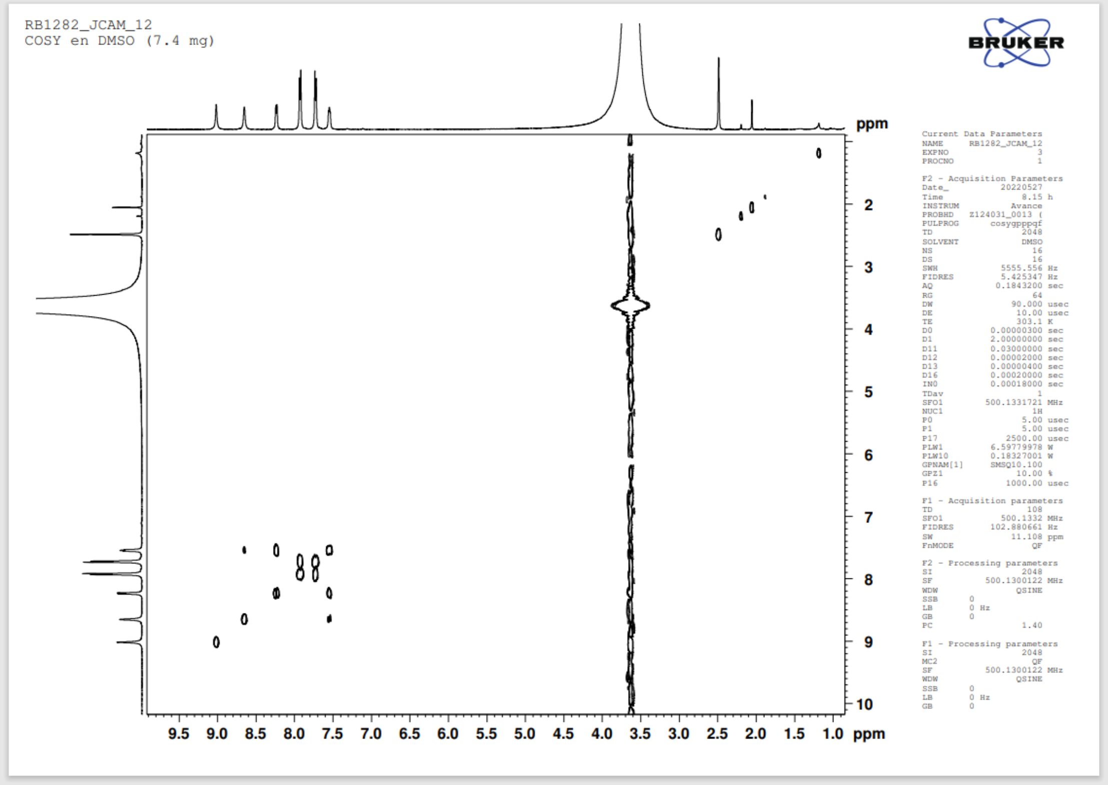

RB1282_JCAM_12
COSY en DMSO (7.4 mg)
BRUKER
Current Data Parameters
ppm
9.5 9.0 8.5 8.0 7.5 7.0 6.5 6.0 5.5 5.0 4.5 4.0 3.5 3.0 2.5 2.0 1.5 1.0 ppm
2 3 4 5 6 7 8 9 10

S7

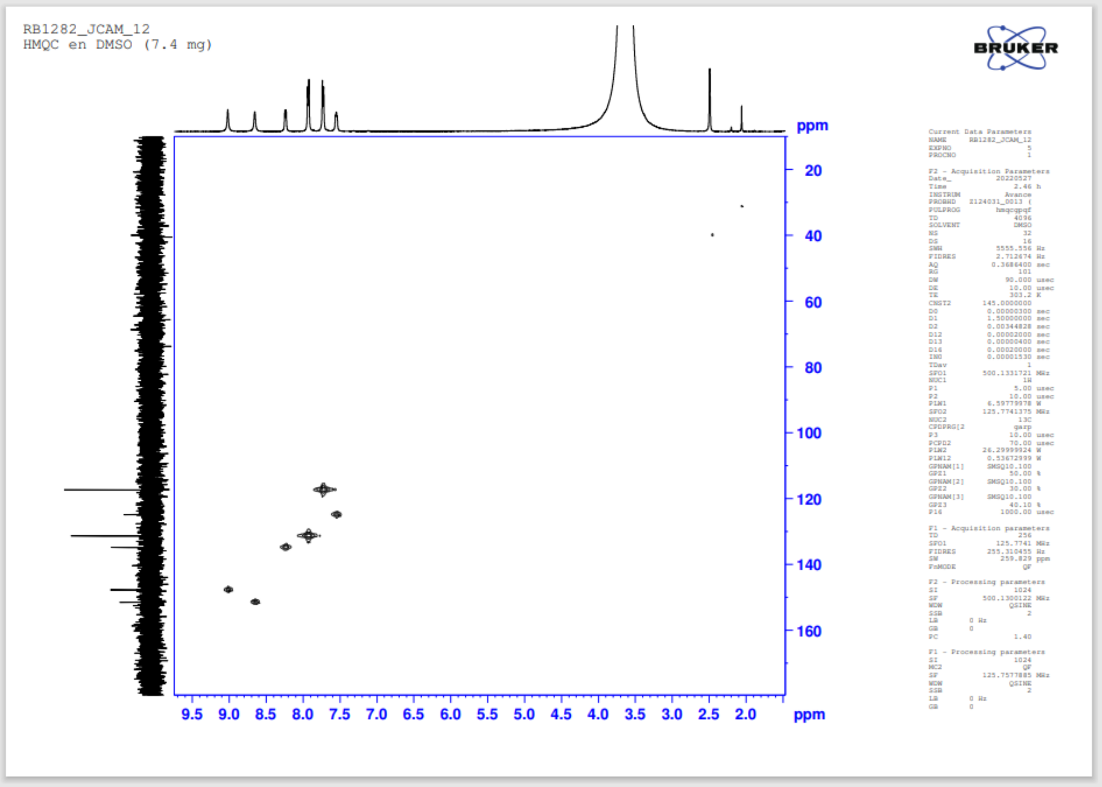
RB1282_JCAM_12
HMQC en DMSO (7.4 mg)
ppm
20
40
60
80
100
120
140
160
9.5 9.0 8.5 8.0 7.5 7.0 6.5 6.0 5.5 5.0 4.5 4.0 3.5 3.0 2.5 2.0
ppm

S8

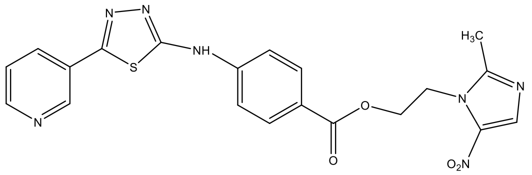

13

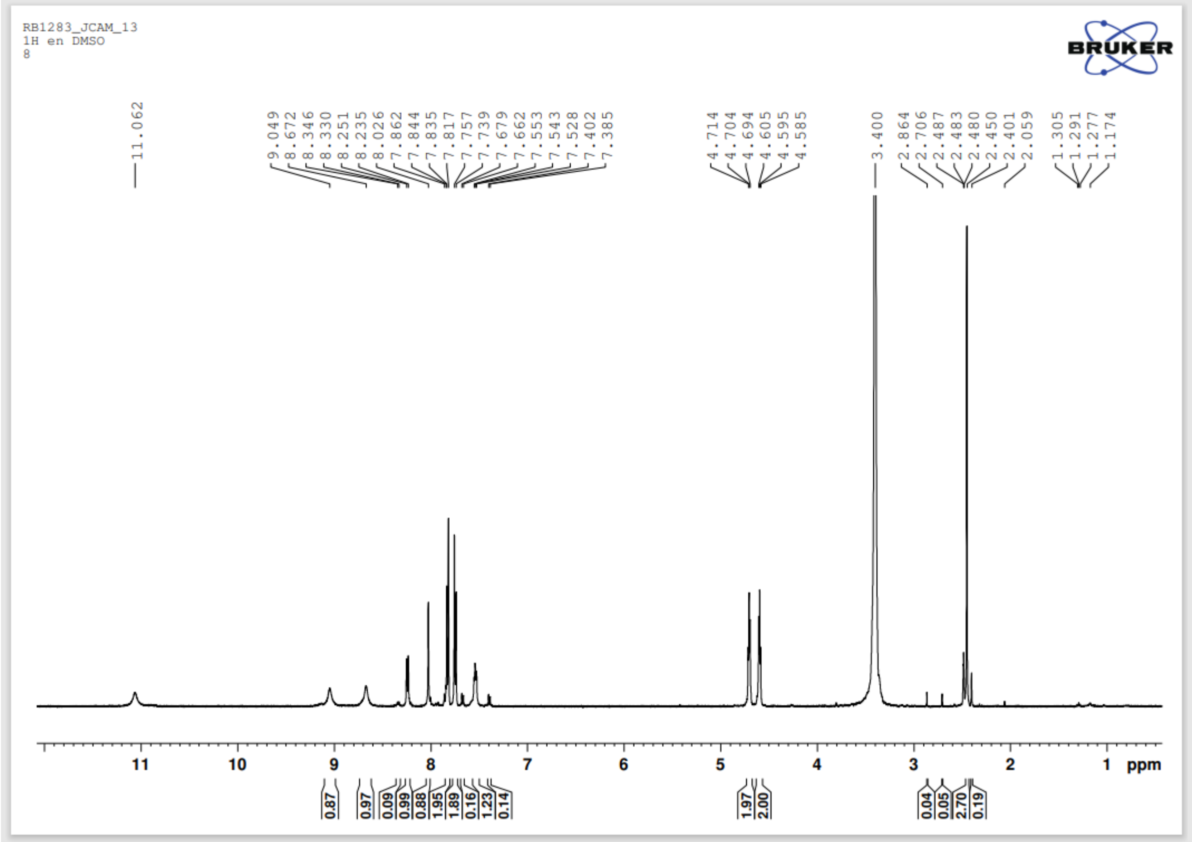

S9

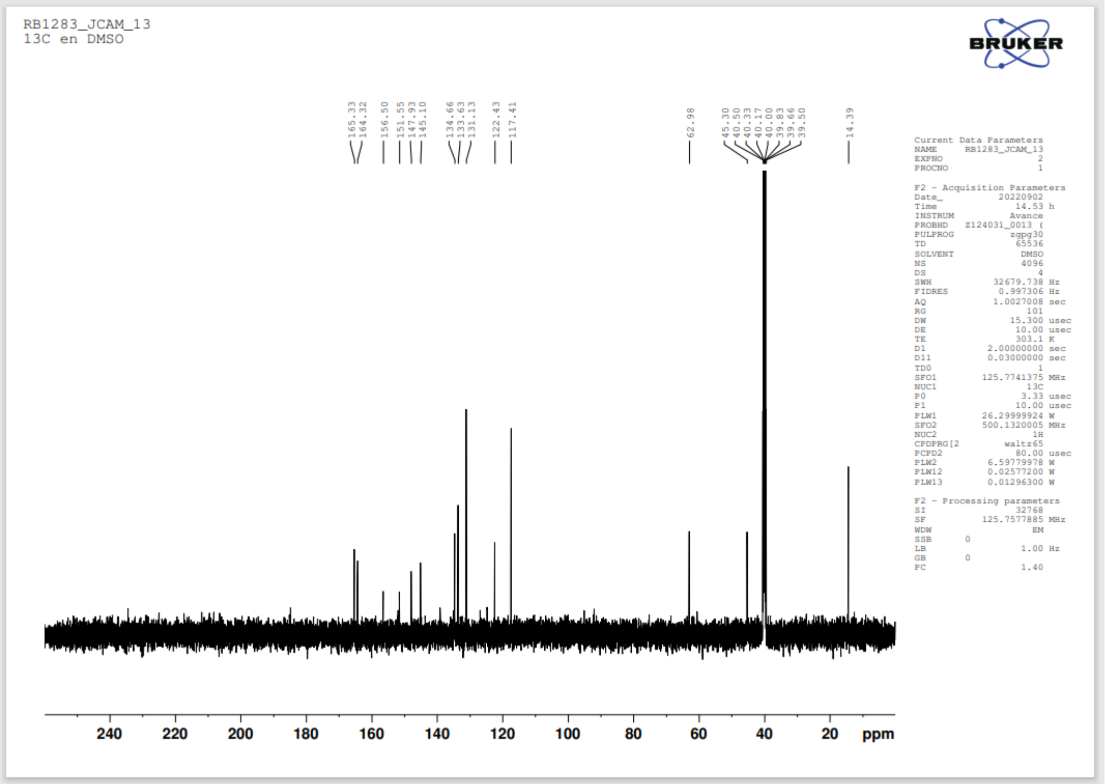
RB1283_JCAM_13
13C en DMSO
BRUKER

S10

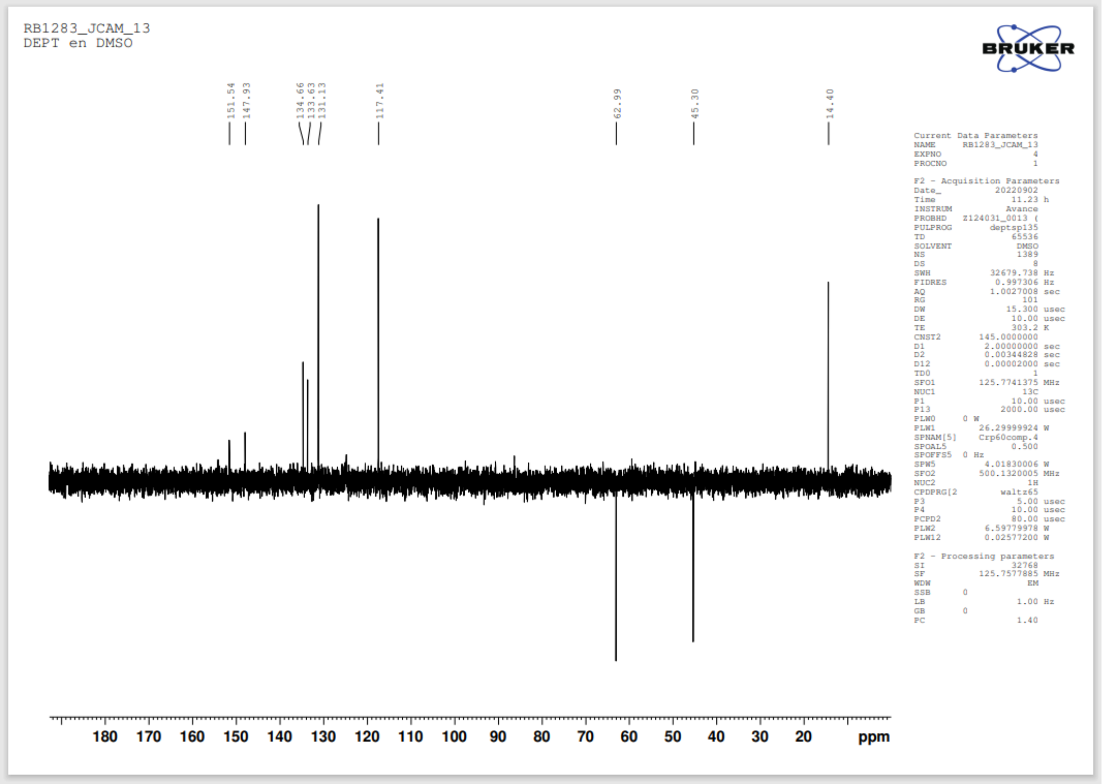
RB1283_JCAM_13
DEPT en DMSO
BRUKER

S11

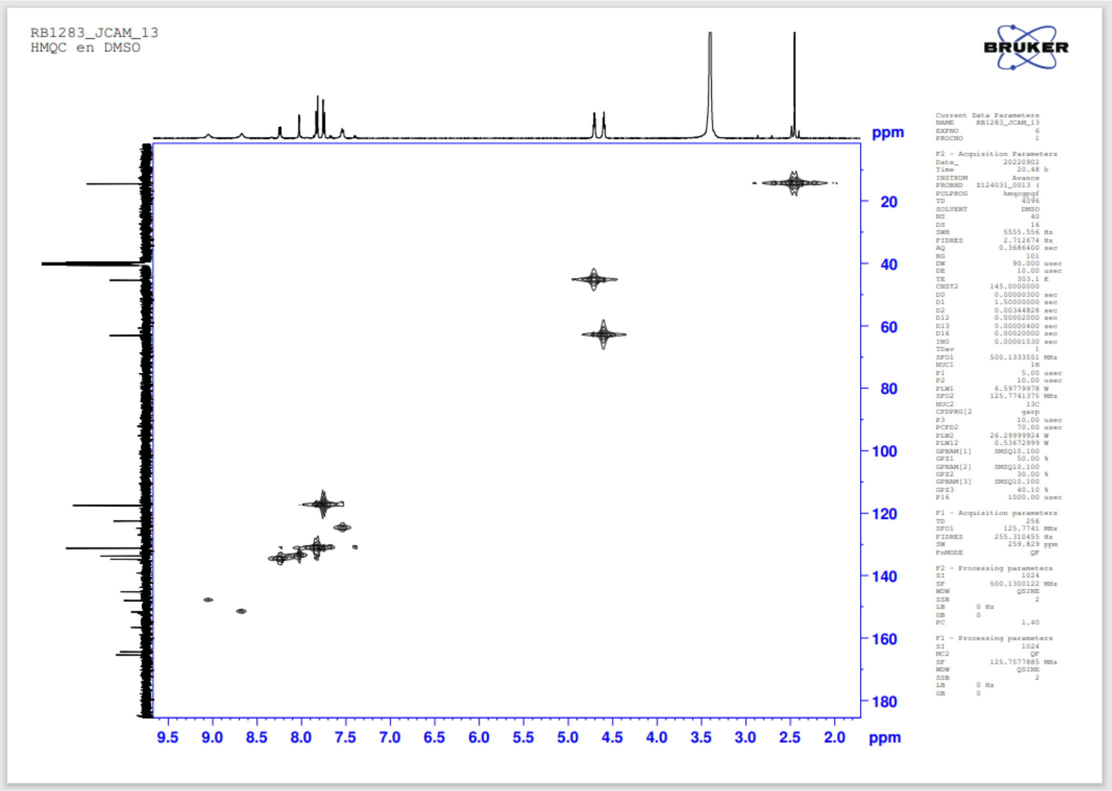

RB1283_JCAM_13
HMQC en DMSO
BRUKER
ppm

S12

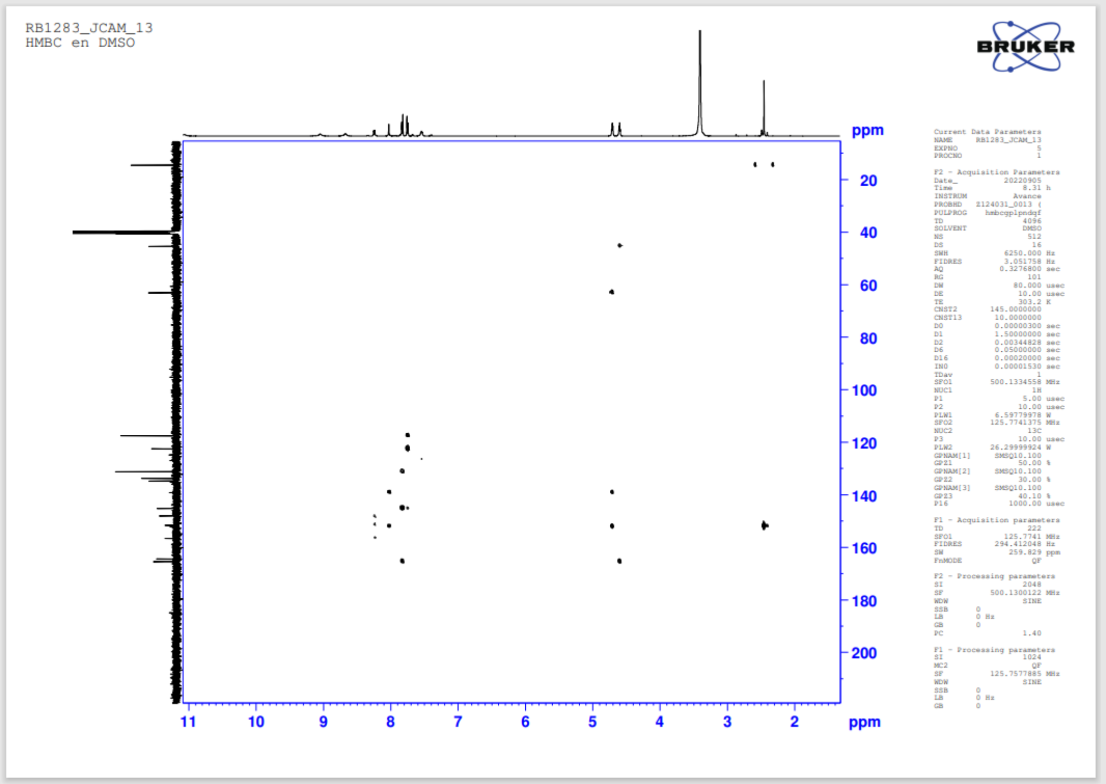

RB1283_JCAM_13
HMBC en DMSO
BRUKER
ppm

S13

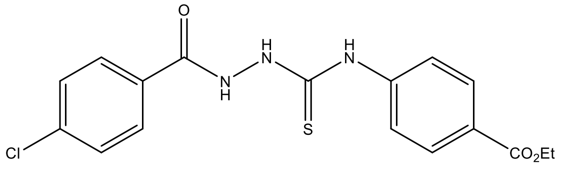
O
H
N
N
H
H
N
S
Cl
CO2Et

7

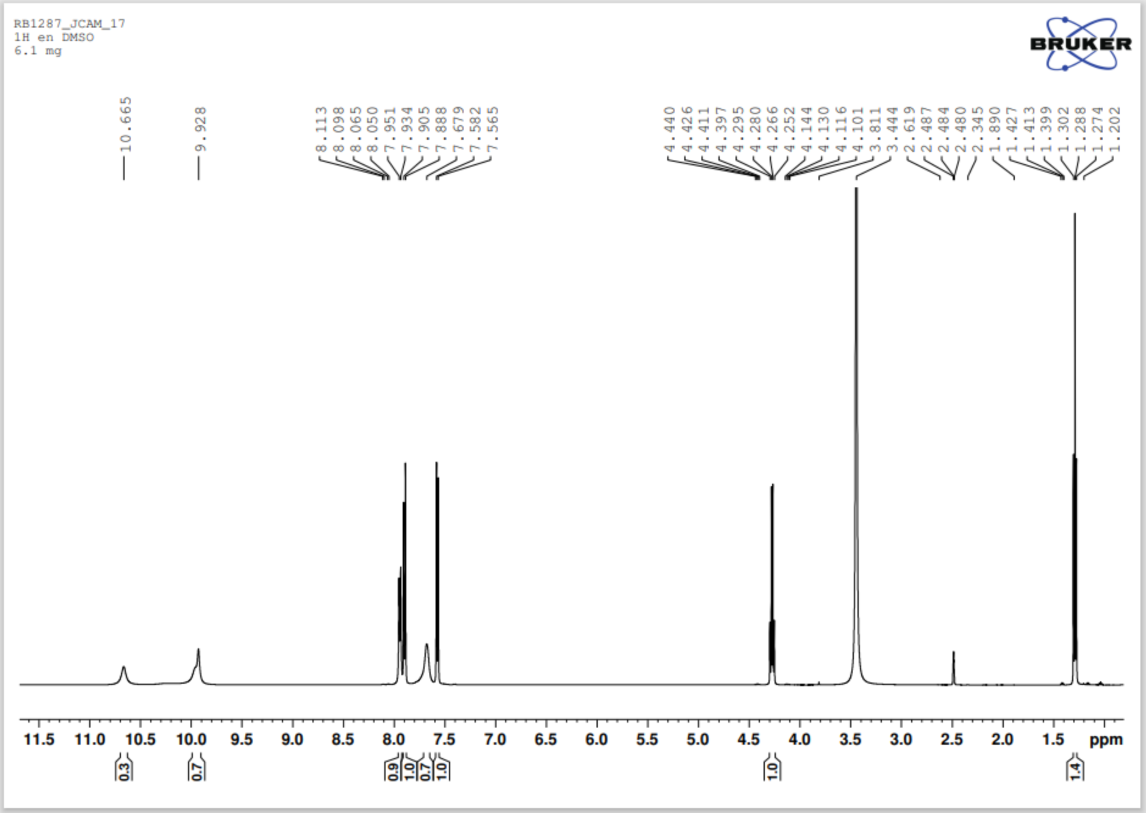
RB1287_JCAM_17
1H en DMSO
6.1 mg
BRUKER
10.665
9.928
8.113
8.098
8.065
8.050
7.951
7.934
7.905
7.888
7.679
7.582
7.565
4.440
4.426
4.411
4.397
4.295
4.280
4.266
4.252
4.144
4.130
4.116
4.101
3.811
3.444
2.619
2.487
2.484
2.480
2.345
1.890
1.427
1.413
1.399
1.302
1.288
1.274
1.202
11.5 11.0 10.5 10.0 9.5 9.0 8.5 8.0 7.5 7.0 6.5 6.0 5.5 5.0 4.5 4.0 3.5 3.0 2.5 2.0 1.5 ppm
0.3
0.7
0.9
1.0
0.7
1.0
1.0
1.4

S14

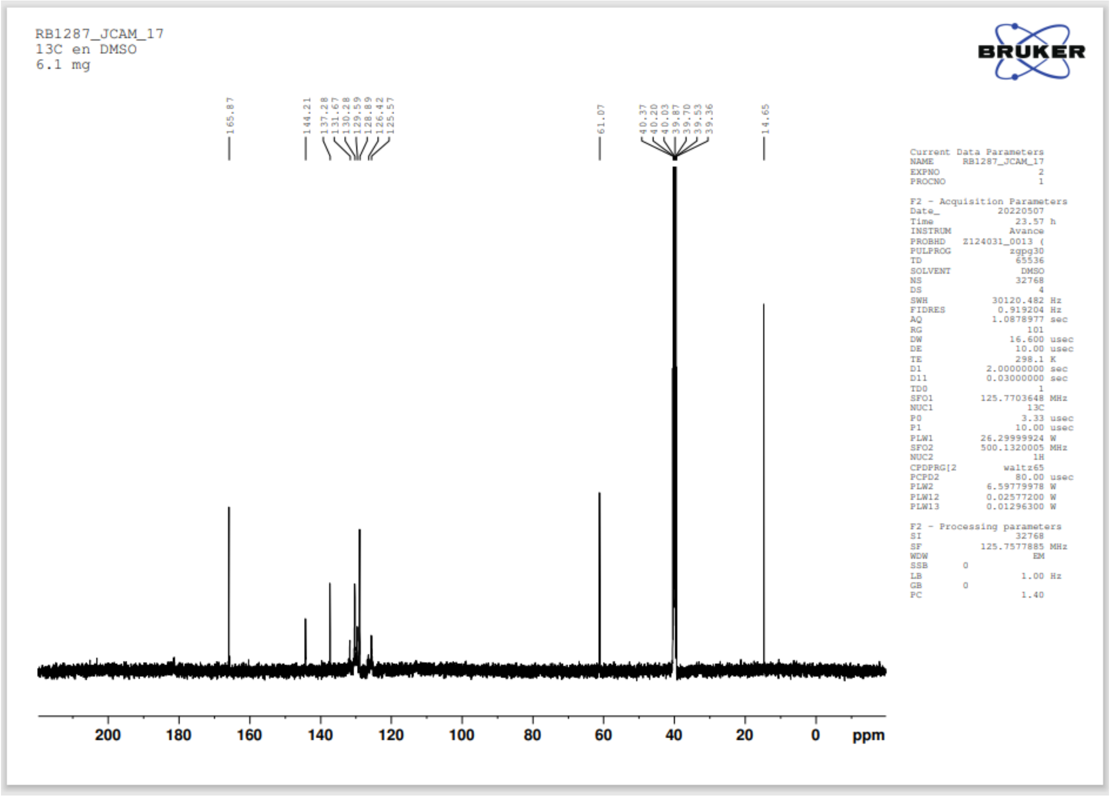
RB1287_JCAM_17
13C en DMSO
6.1 mg
BRUKER
165.87
144.21
137.28
131.67
130.28
129.59
128.89
126.42
125.57
61.07
40.37
40.20
40.03
39.87
39.70
39.53
39.36
14.65
200 180 160 140 120 100 80 60 40 20 0 ppm

S15

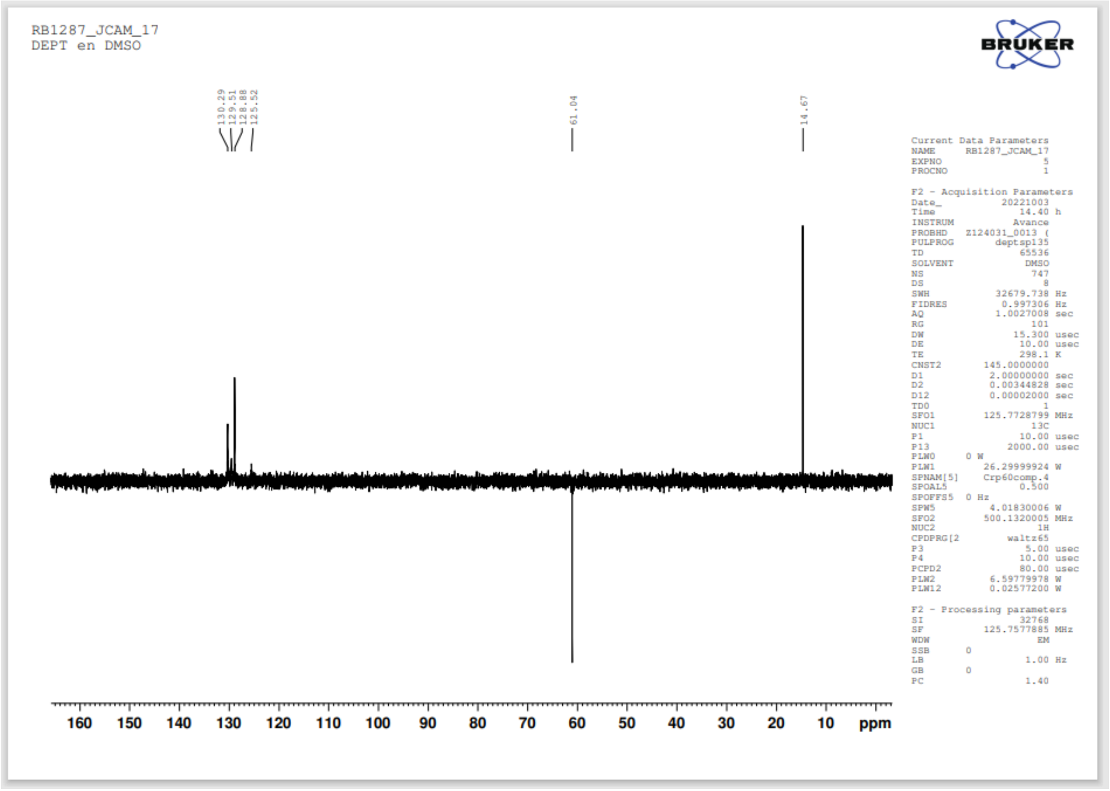
RB1287_JCAM_17
DEPT en DMSO
BRUKER
130.29
129.51
128.88
125.52
61.04
14.67
160 150 140 130 120 110 100 90 80 70 60 50 40 30 20 10 ppm

S16

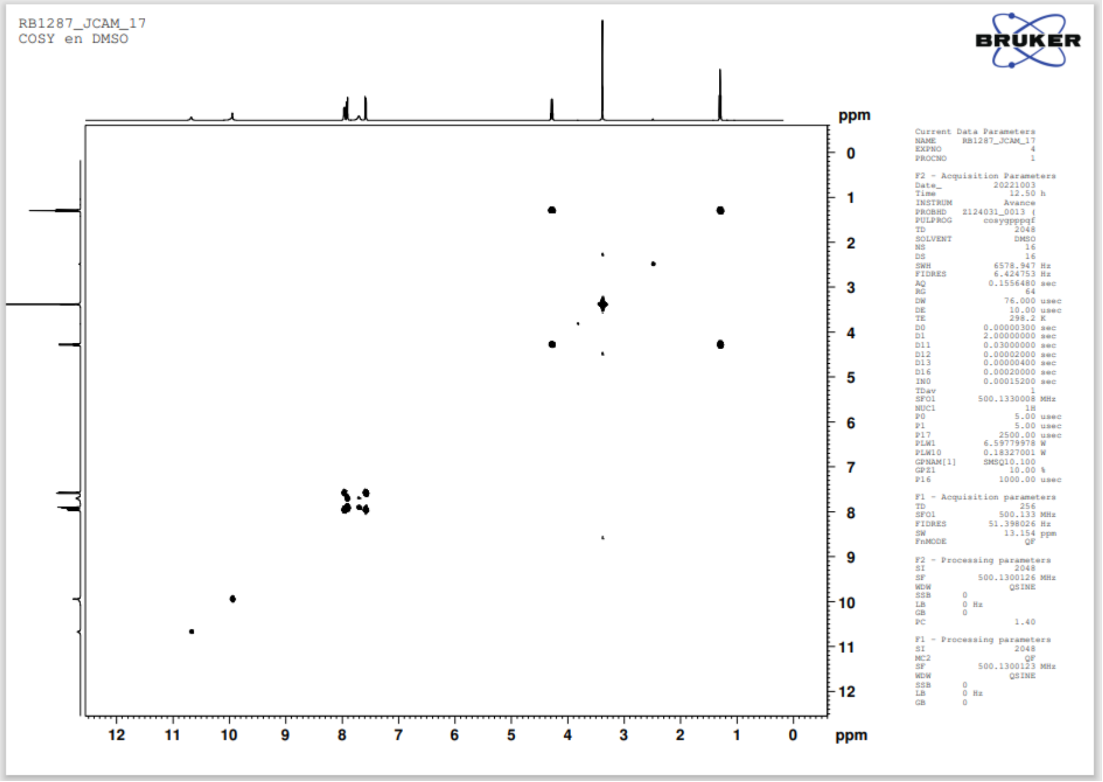
RB1287_JCAM_17
COSY en DMSO
BRUKER
ppm
0
1
2
3
4
5
6
7
8
9
10
11
12
12 11 10 9 8 7 6 5 4 3 2 1 0 ppm
Current Data Parameters
NAME RB1287_JCAM_17
EXPNO 4
PROCNO 1
F2 - Acquisition Parameters
Date_ 20221003
Time 12.50 h
INSTRUM Avance
PULPROG cosygpppqf
TD 2048
SOLVENT DMSO
NS 16
DS 16
SWH 6578.947 Hz
FIDRES 6.424753 Hz
AQ 0.1556480 sec
RG 64
DW 76.000 usec
DE 10.00 usec
TE 298.2 K
SFO1 500.1330008 MHz
NUC1 1H
F1 - Acquisition parameters
TD 256
SFO1 500.133 MHz
FIDRES 51.398026 Hz
SW 13.154 ppm
FnMODE QF
F2 - Processing parameters
SI 2048
SF 500.1300126 MHz
WDW QSINE
PC 1.40
F1 - Processing parameters
SI 2048
MC2 QF
SF 500.1300123 MHz
WDW QSINE

S17

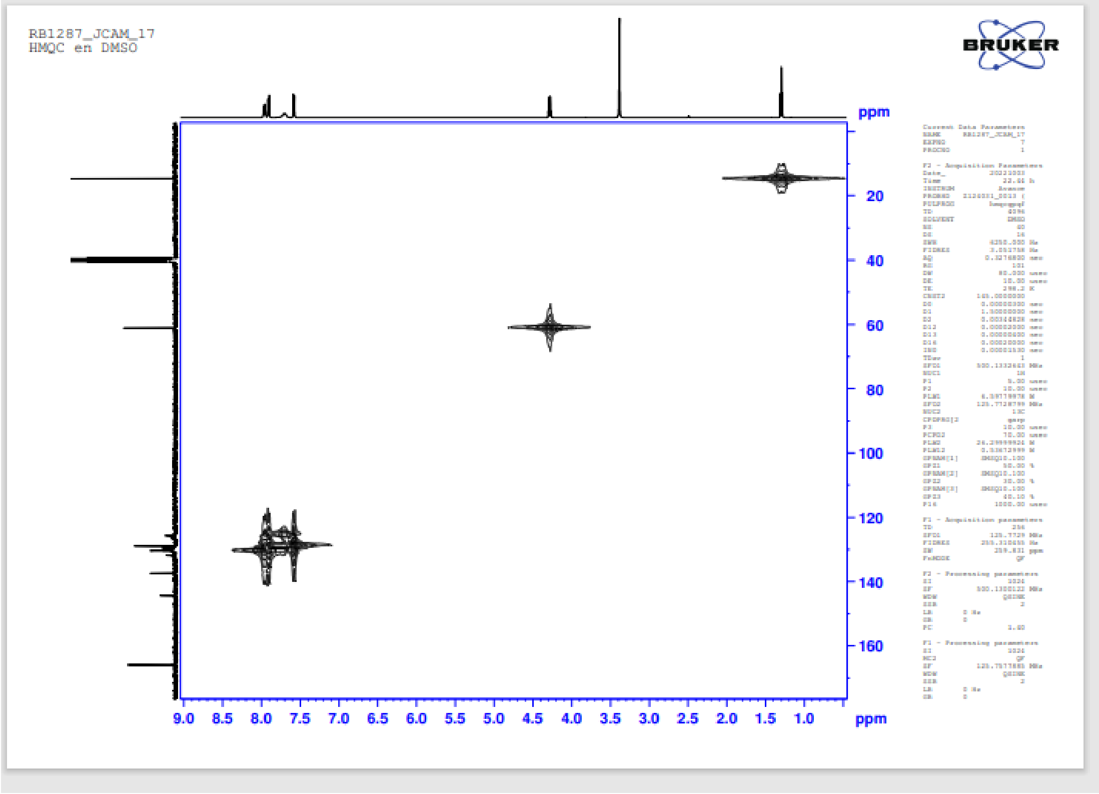
RB1287_JCAM_17
HMQC en DMSO
BRUKER
ppm
20
40
60
80
100
120
140
160
9.0 8.5 8.0 7.5 7.0 6.5 6.0 5.5 5.0 4.5 4.0 3.5 3.0 2.5 2.0 1.5 1.0 ppm

S18

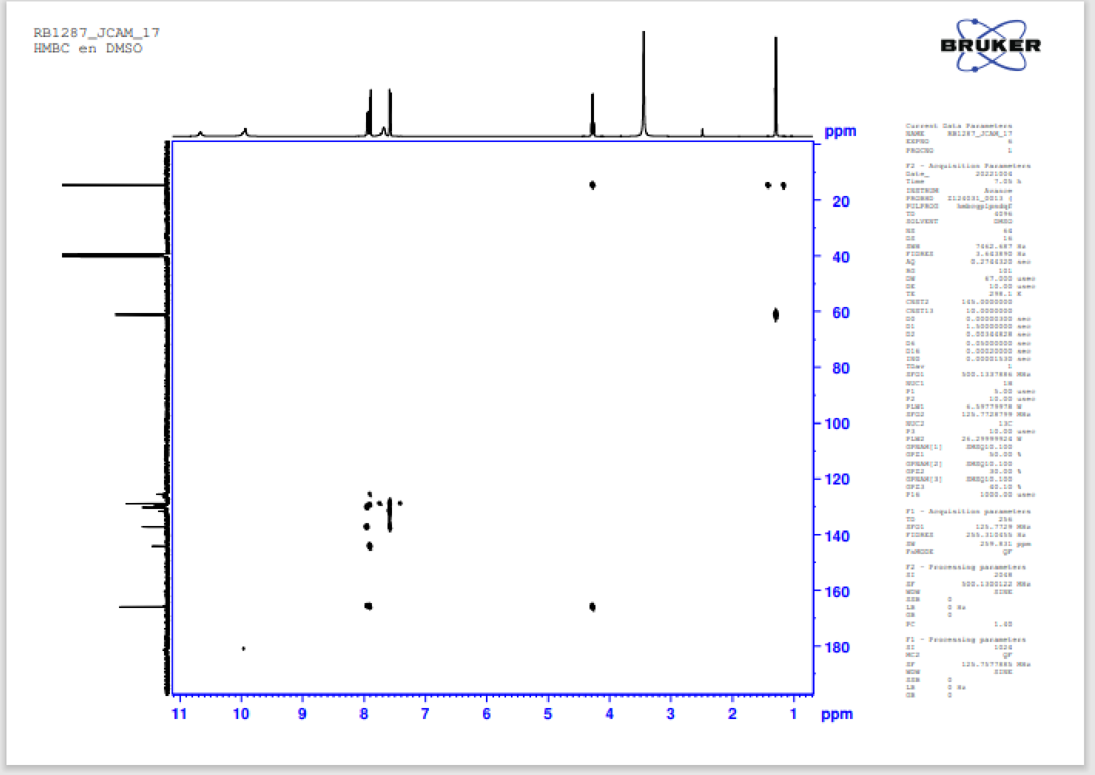

RB1287_JCAM_17
HMBC en DMSO
BRUKER
ppm
20
40
60
80
100
120
140
160
180
11
10
9
8
7
6
5
4
3
2
1
ppm

S19

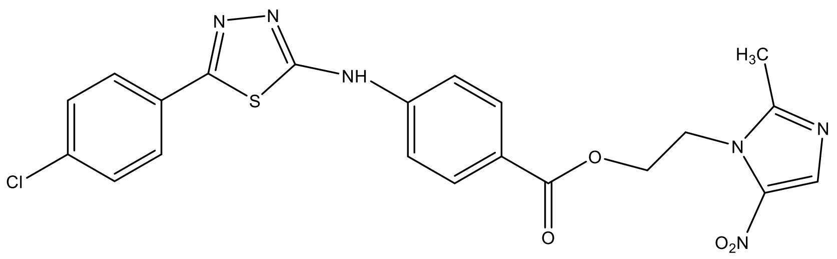

20

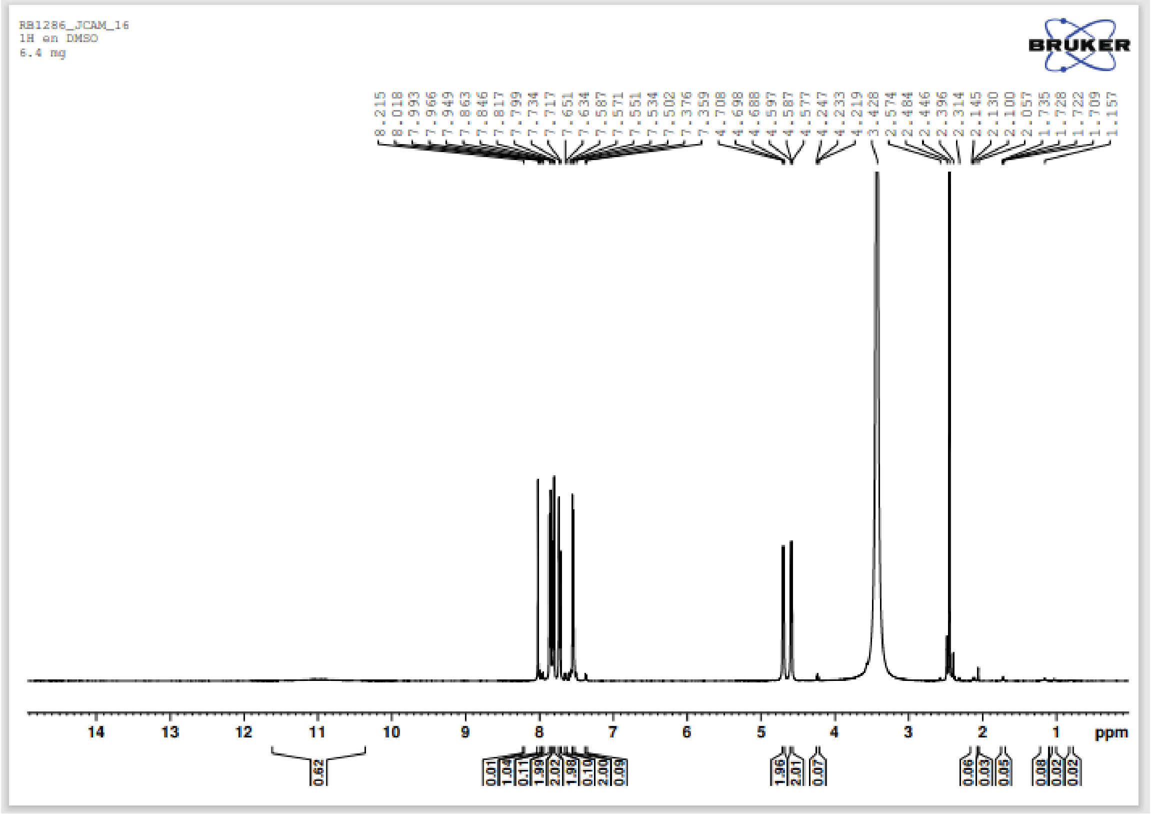

S20

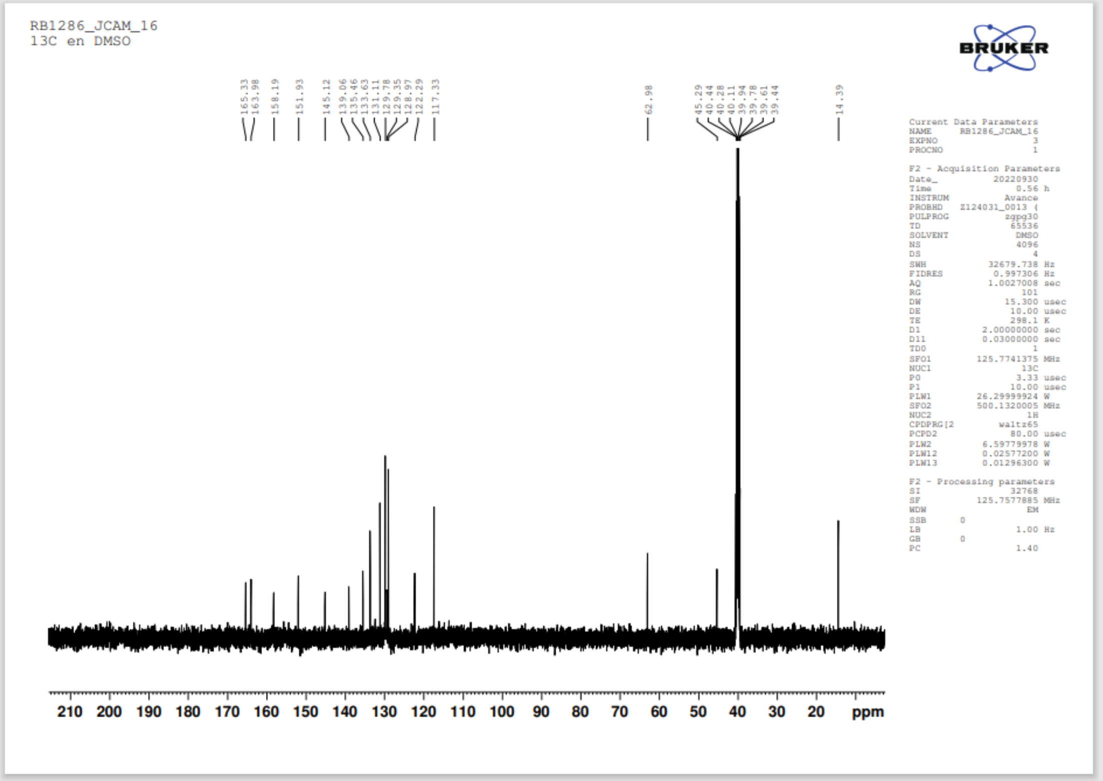
RB1286_JCAM_16
13C en DMSO
165.33
163.98
158.19
151.93
145.12
139.06
135.46
133.63
131.11
129.78
129.35
128.97
122.29
117.33
62.98
45.29
40.44
40.28
40.11
39.94
39.78
39.61
39.44
14.39
BRUKER
210 200 190 180 170 160 150 140 130 120 110 100 90 80 70 60 50 40 30 20 ppm

S21

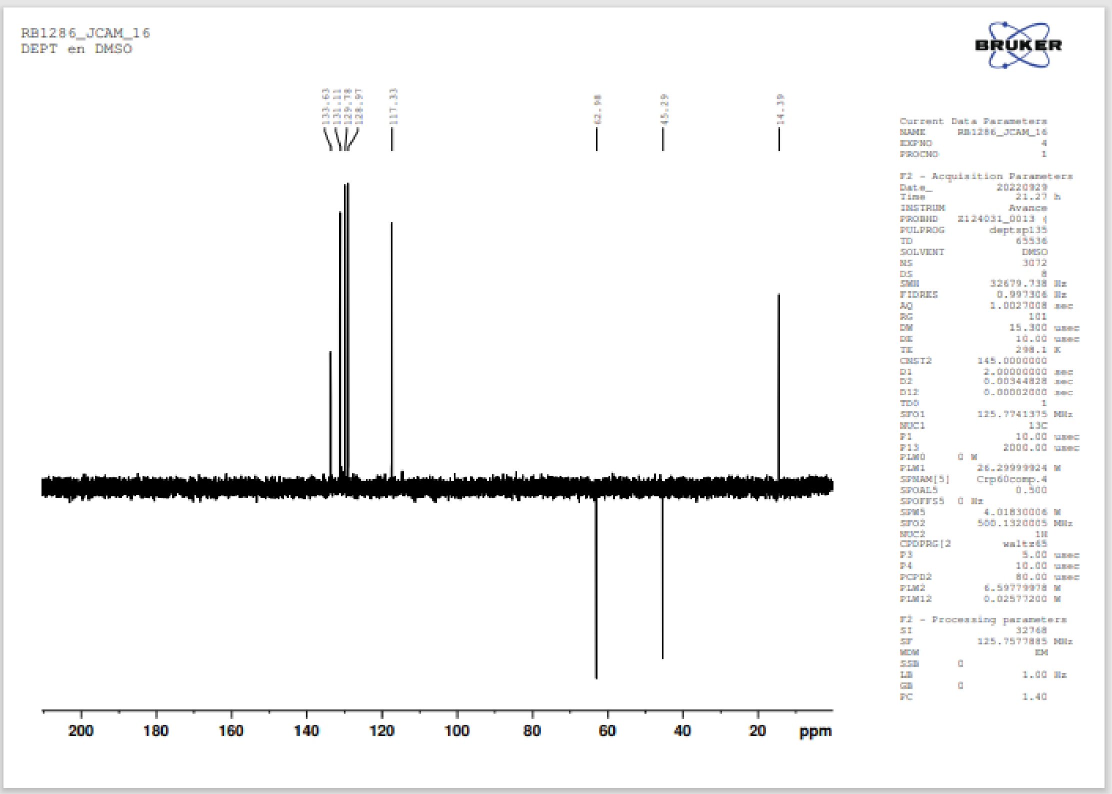
RB1286_JCAM_16
DEPT en DMSO
133.63
131.11
129.78
128.97
117.33
62.98
45.29
14.39
BRUKER
200 180 160 140 120 100 80 60 40 20 ppm

S22

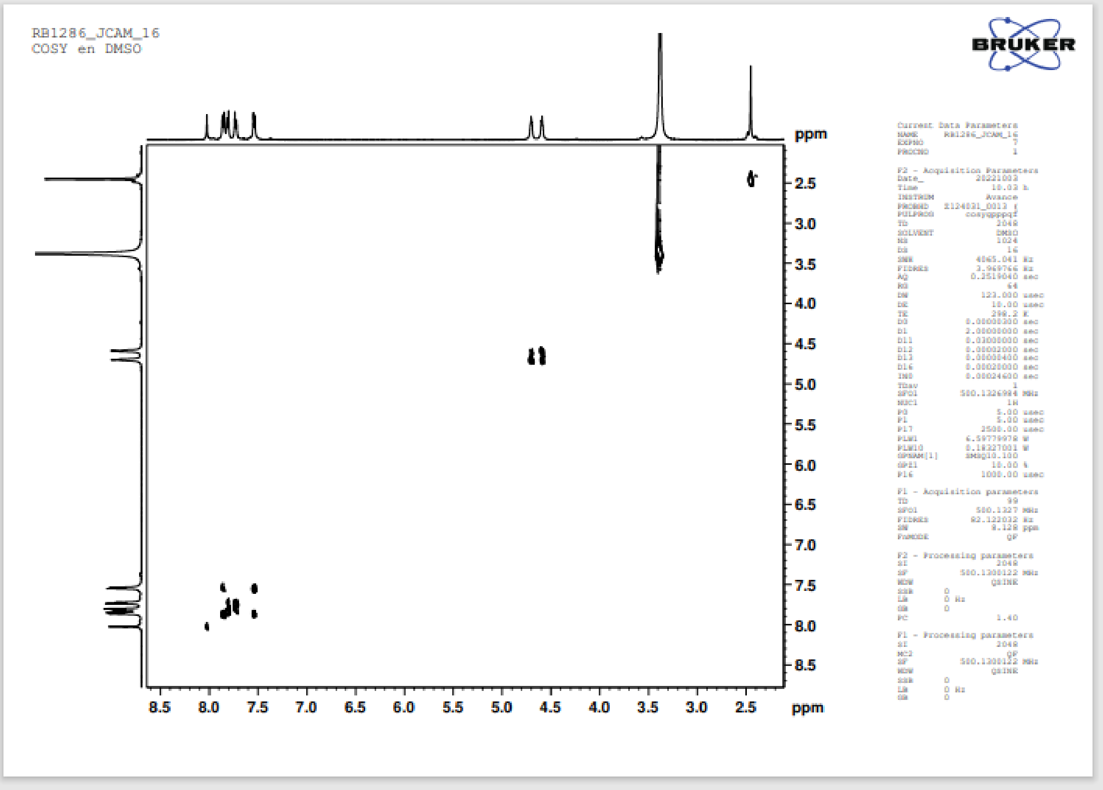
RB1286_JCAM_16
COSY en DMSO
BRUKER
ppm
2.5
3.0
3.5
4.0
4.5
5.0
5.5
6.0
6.5
7.0
7.5
8.0
8.5
8.5 8.0 7.5 7.0 6.5 6.0 5.5 5.0 4.5 4.0 3.5 3.0 2.5 ppm

S23

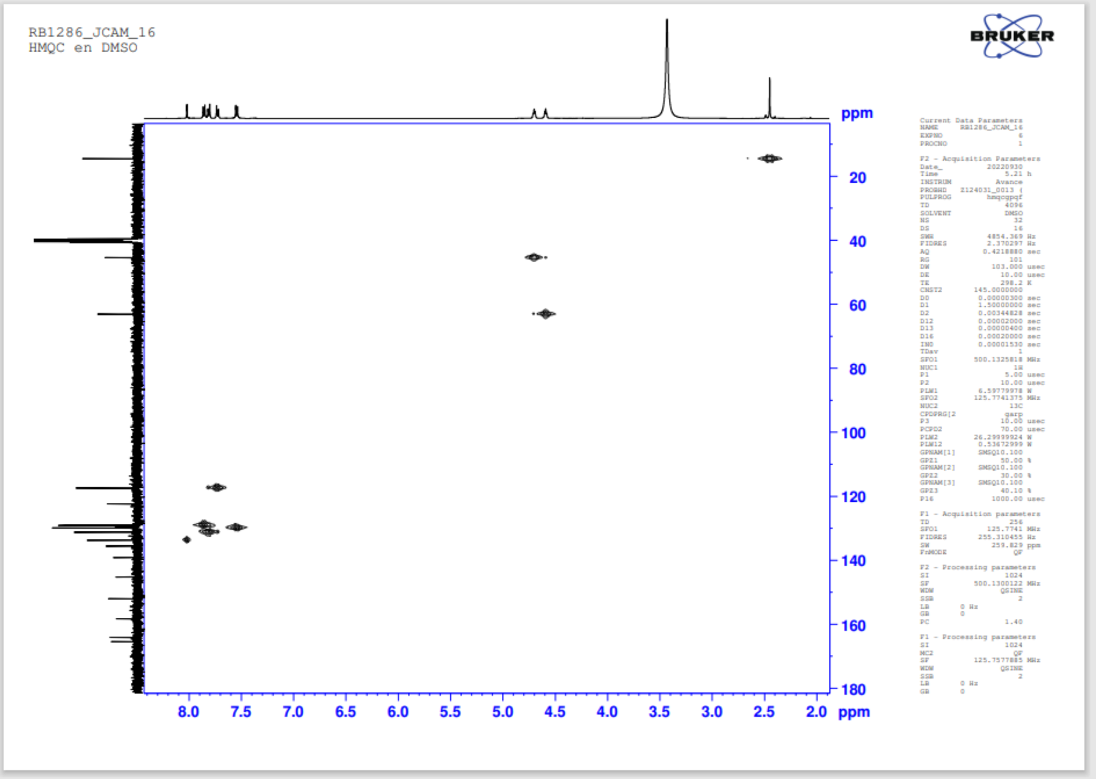
RB1286_JCAM_16
HMQC en DMSO
BRUKER
ppm
20
40
60
80
100
120
140
160
180
8.0 7.5 7.0 6.5 6.0 5.5 5.0 4.5 4.0 3.5 3.0 2.5 2.0 ppm

S24

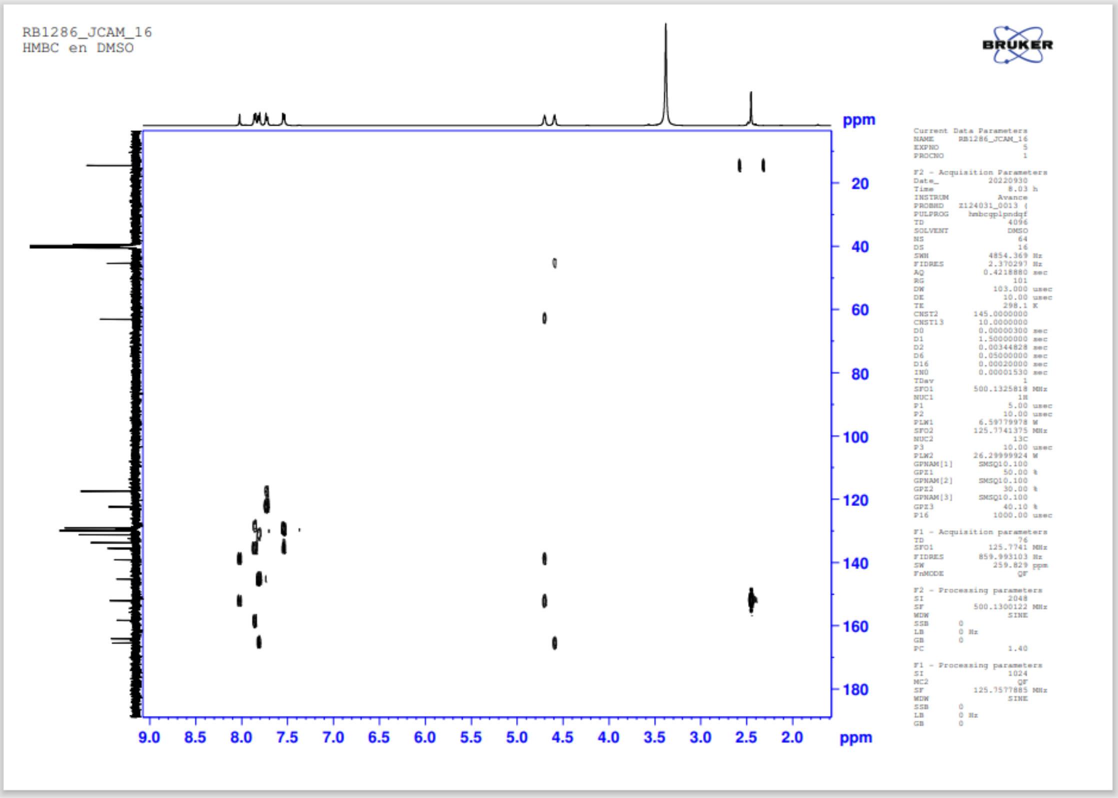
RB1286_JCAM_16
HMBC en DMSO
ppm
20
40
60
80
100
120
140
160
180
9.0
8.5
8.0
7.5
7.0
6.5
6.0
5.5
5.0
4.5
4.0
3.5
3.0
2.5
2.0
ppm
Current Data Parameters
NAME RB1286_JCAM_16
EXPNO 5
PROCNO 1
F2 - Acquisition Parameters
Date_ 20220930
Time 8.03 h
INSTRUM Avance
PROBHD Z124031_0013 (
PULPROG hmbcgplpndqf
TD 4096
SOLVENT DMSO
NS 64
DS 16
SWH 4854.369 Hz
FIDRES 2.370297 Hz
AQ 0.4218880 sec
RG 101
DW 103.000 usec
DE 10.00 usec
TE 298.1 K
CNST2 145.0000000
CNST13 10.0000000
D0 0.00000300 sec
D1 1.50000000 sec
D2 0.00344828 sec
D6 0.05000000 sec
D16 0.00020000 sec
IN0 0.00001530 sec
TDav 1
SFO1 500.1325818 MHz
NUC1 1H
P1 5.00 usec
P2 10.00 usec
PLW1 6.59779978 W
SFO2 125.7741375 MHz
NUC2 13C
P3 10.00 usec
PLW2 26.29999924 W
GPNAM[1] SMSQ10.100
GPZ1 50.00 %
GPNAM[2] SMSQ10.100
GPZ2 30.00 %
GPNAM[3] SMSQ10.100
GPZ3 40.10 %
P16 1000.00 usec
F1 - Acquisition parameters
TD 76
SFO1 125.7741 MHz
FIDRES 859.993103 Hz
SW 259.829 ppm
FnMODE QF
F2 - Processing parameters
SI 2048
SF 500.1300122 MHz
WDW SINE
SSB 0
LB 0 Hz
GB 0
PC 1.40
F1 - Processing parameters
SI 1024
MC2 QF
SF 125.7577885 MHz
WDW SINE
SSB 0
LB 0 Hz
GB 0

S25

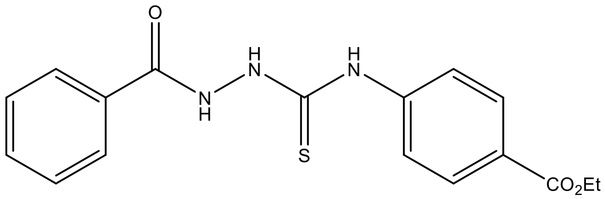

O
H
N
N
H
H
N
S
CO_2Et

8

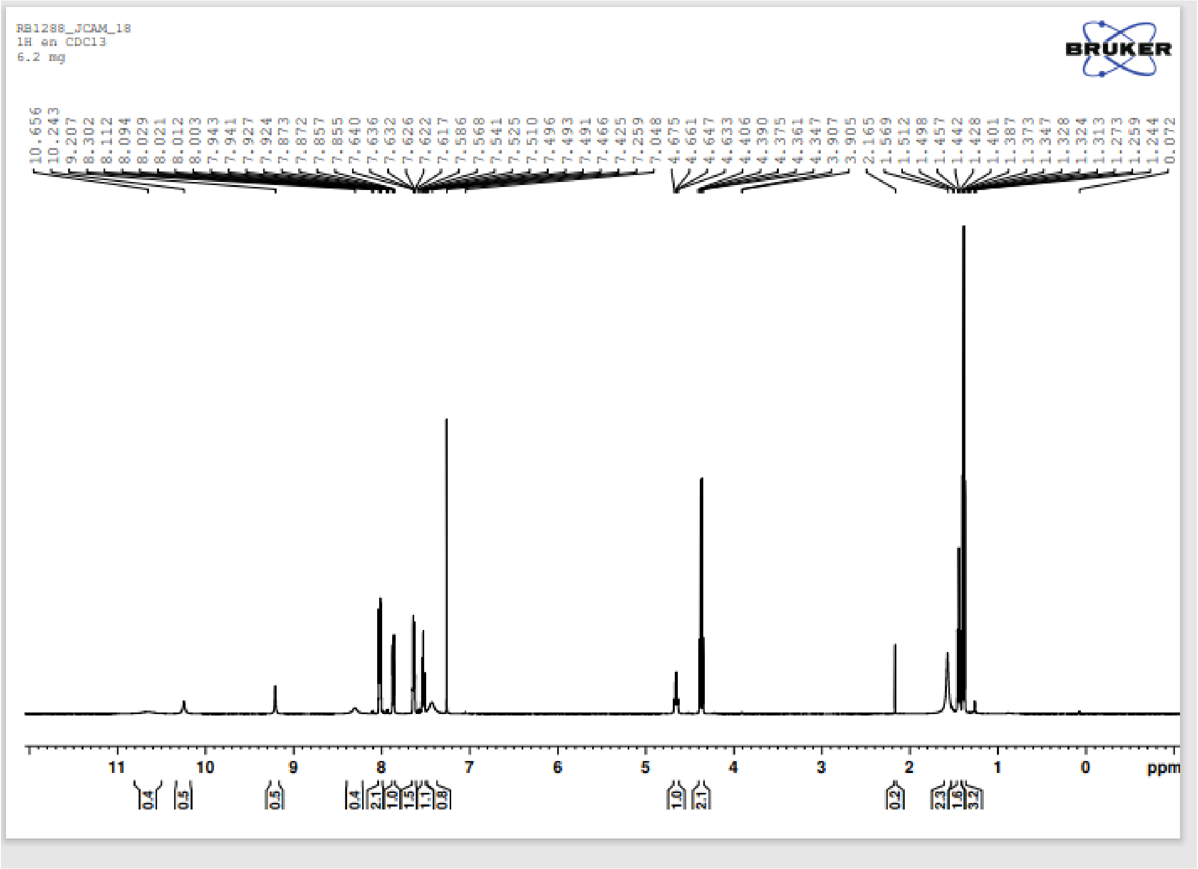

RB1288_JCAM_18
1H en CDCl3
6.2 mg
BRUKER
10.656 10.243 9.207 8.302 8.112 8.094 8.029 8.021 8.012 8.003 7.943 7.941 7.927 7.924 7.873 7.872 7.857 7.855 7.640 7.636 7.632 7.626 7.622 7.617 7.586 7.568 7.541 7.525 7.510 7.496 7.493 7.491 7.466 7.425 7.259 7.048 4.675 4.661 4.647 4.633 4.406 4.390 4.375 4.361 4.347 3.907 3.905 2.165 1.569 1.512 1.498 1.457 1.442 1.428 1.401 1.387 1.373 1.347 1.328 1.324 1.313 1.273 1.259 1.244 0.072
11 10 9 8 7 6 5 4 3 2 1 0 ppm
0.4 0.5 0.5 0.4 2.1 1.0 1.5 1.1 0.8 1.0 2.1 0.2 2.3 1.6 3.2

S26

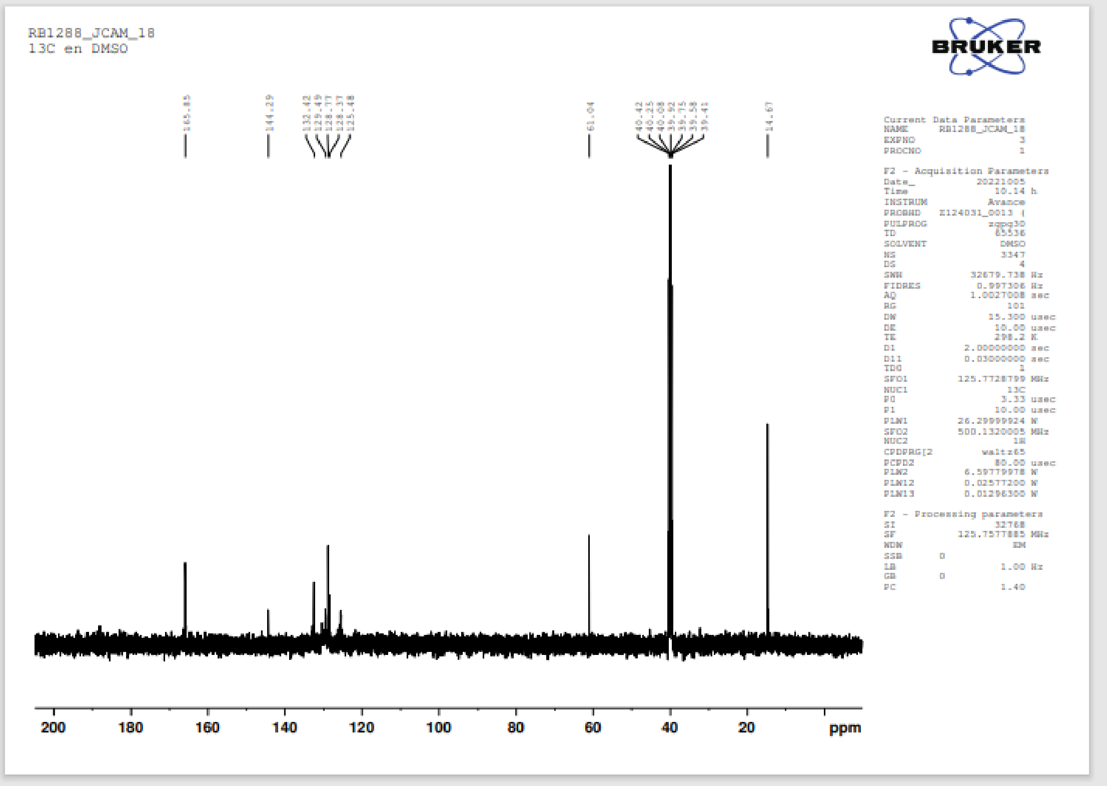

RB1288_JCAM_18
13C en DMSO
BRUKER
200 180 160 140 120 100 80 60 40 20 ppm

S27

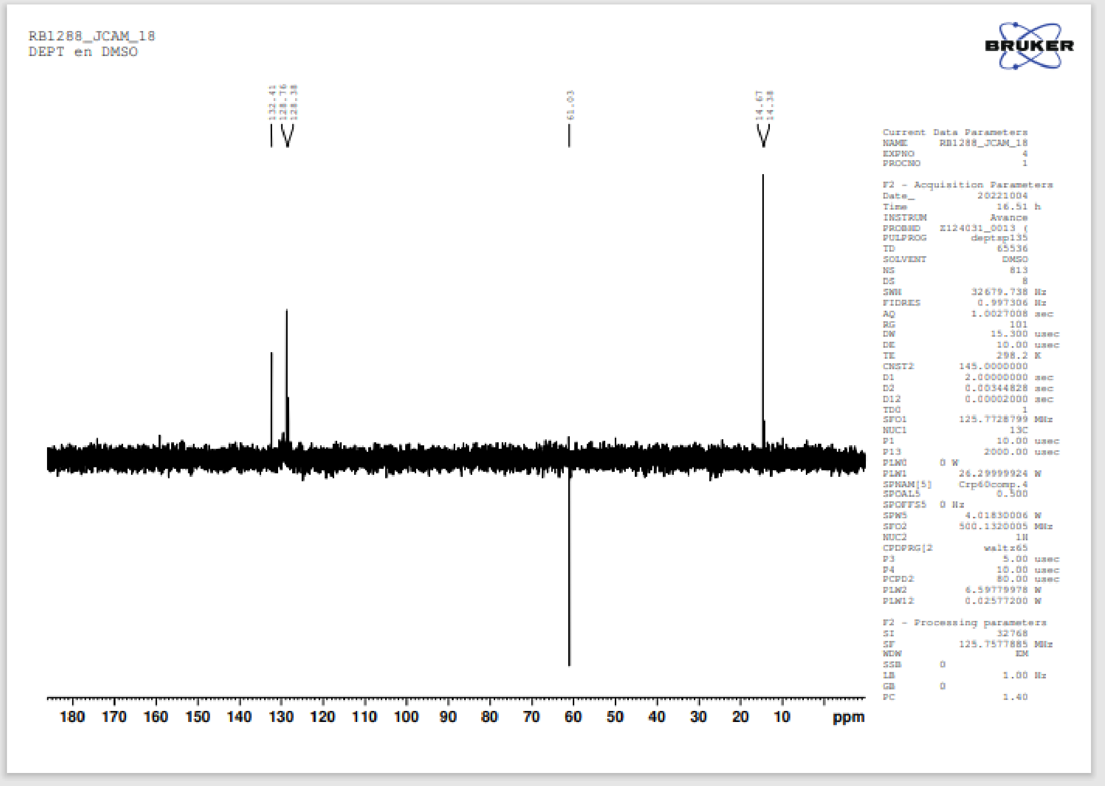

RB1288_JCAM_18
DEPT en DMSO
BRUKER
180 170 160 150 140 130 120 110 100 90 80 70 60 50 40 30 20 10 ppm

S28

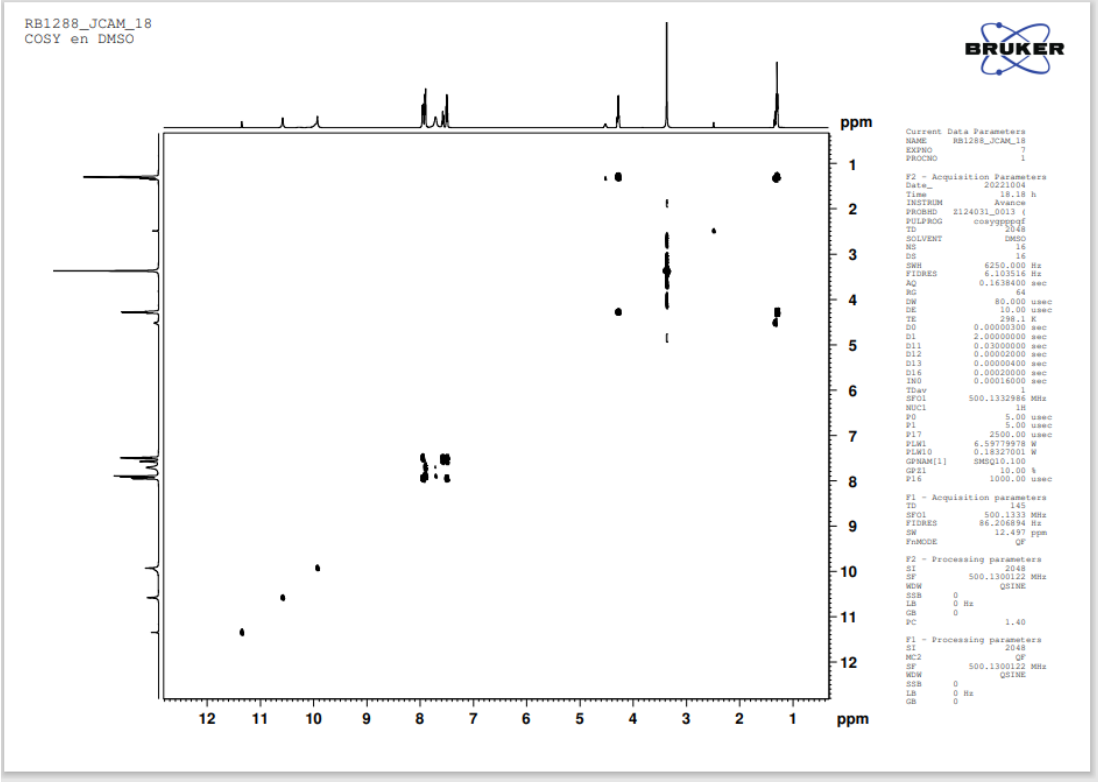
RB1288_JCAM_18
COSY en DMSO
ppm

S29

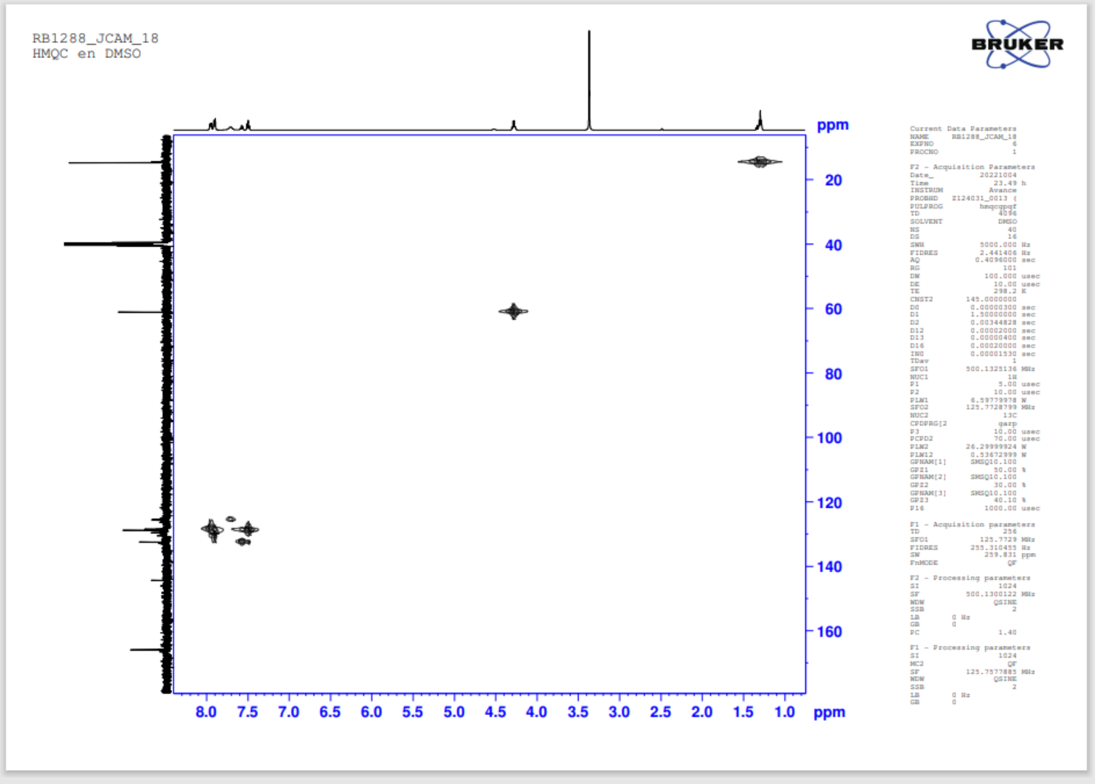
RB1288_JCAM_18
HMQC en DMSO
ppm

S30

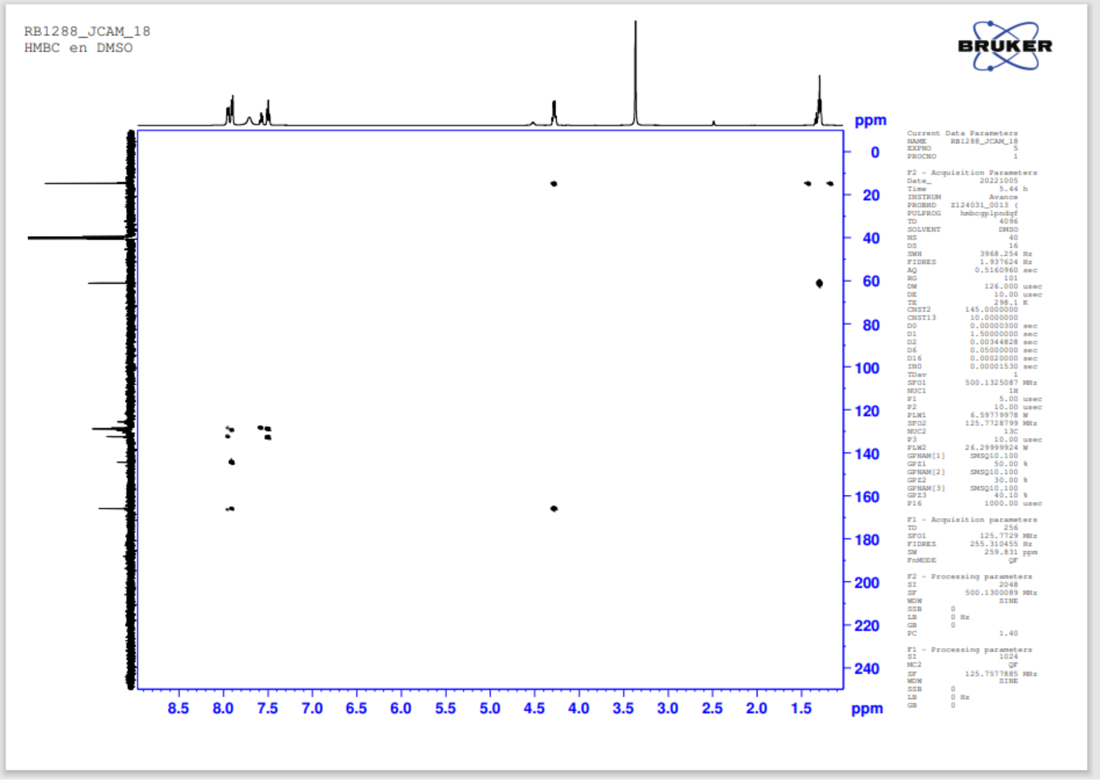
RB1288_JCAM_18
HMBC en DMSO
BRUKER
ppm
0
20
40
60
80
100
120
140
160
180
200
220
240
8.5
8.0
7.5
7.0
6.5
6.0
5.5
5.0
4.5
4.0
3.5
3.0
2.5
2.0
1.5
ppm

S31

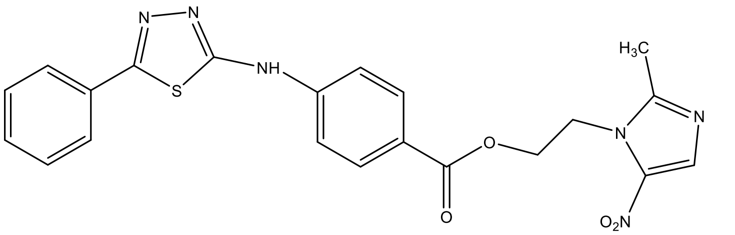
N
N
S
NH
O
O
H3C
N
N
O2N

21

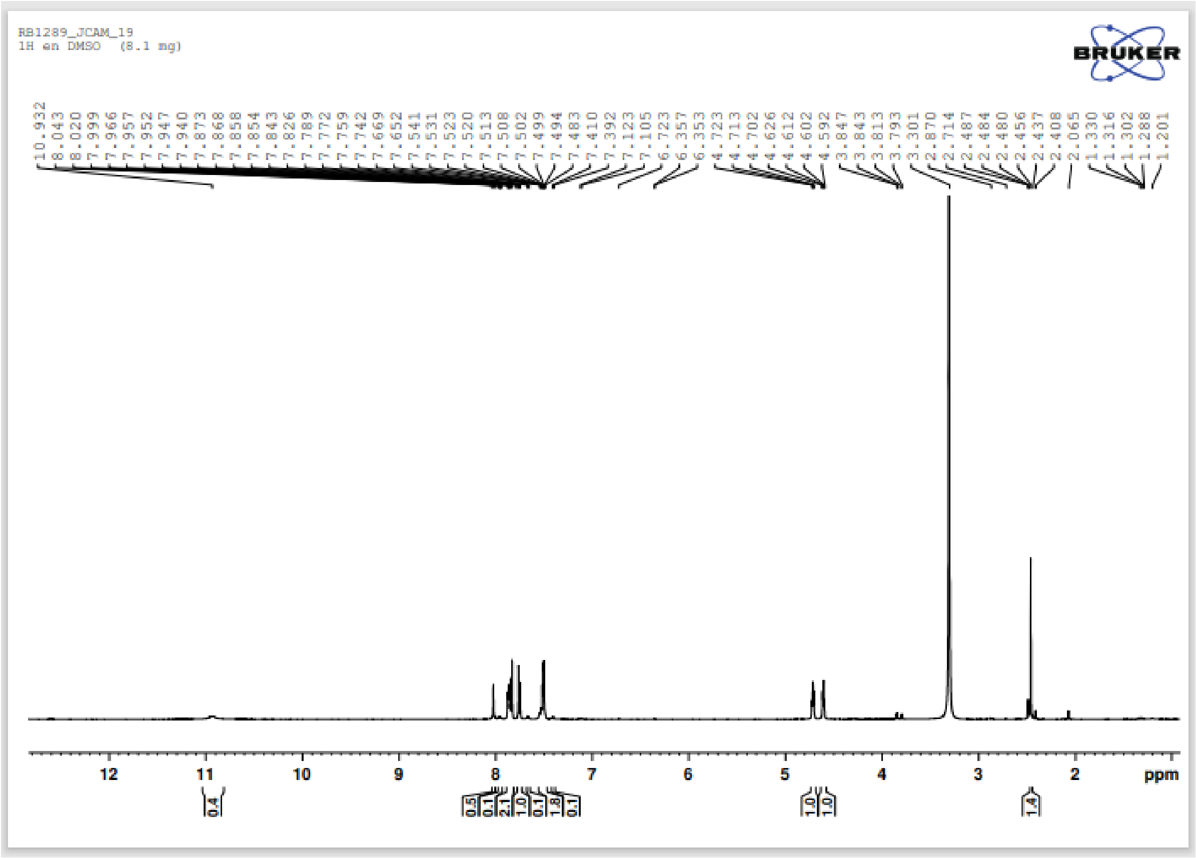
RB1289_JCAM_19
1H en DMSO (8.1 mg)
BRUKER
12
11
10
9
8
7
6
5
4
3
2
ppm

S32

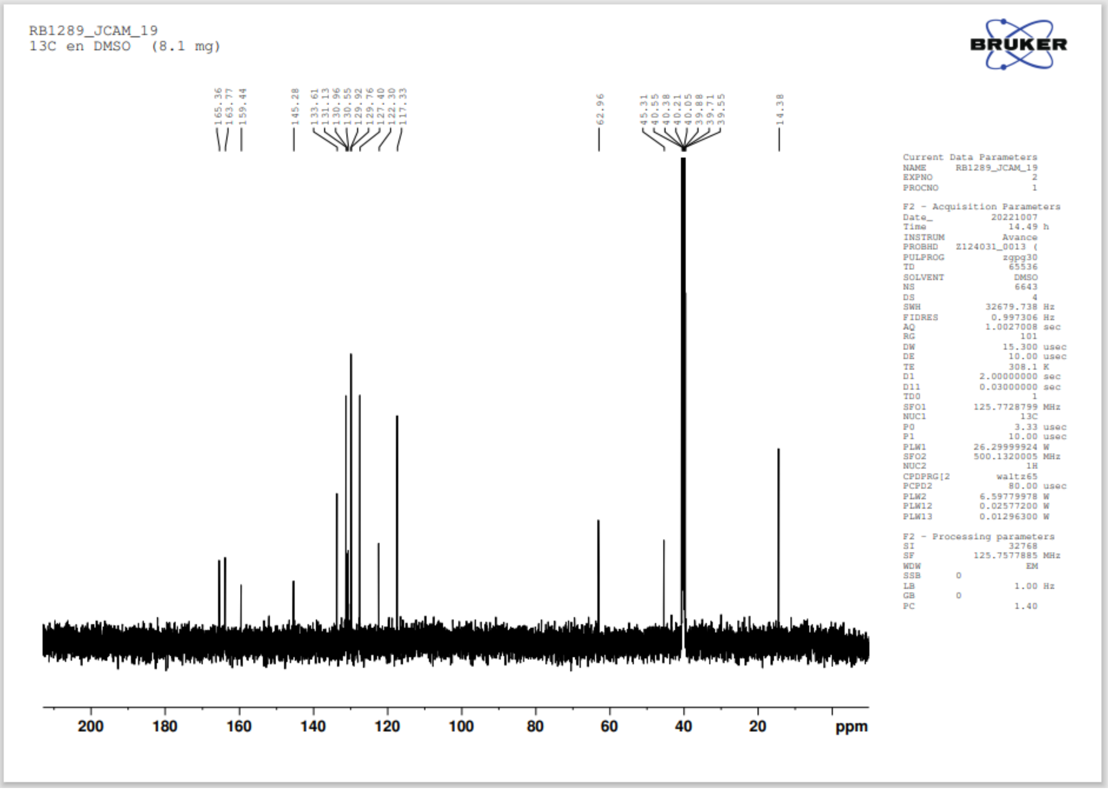
RB1289_JCAM_19
13C en DMSO (8.1 mg)
165.36
163.77
159.44
145.28
133.61
131.13
130.96
130.55
129.92
129.76
127.40
122.30
117.33
62.96
45.31
40.55
40.38
40.21
40.05
39.88
39.71
39.55
14.38
Current Data Parameters
NAME RB1289_JCAM_19
EXPNO 2
PROCNO 1
F2 - Acquisition Parameters
Date_ 20221007
Time 14.49 h
INSTRUM Avance
PROBHD Z124031_0013 (
PULPROG zgpg30
TD 65536
SOLVENT DMSO
NS 6643
DS 4
SWH 32679.738 Hz
FIDRES 0.997306 Hz
AQ 1.0027008 sec
RG 101
DW 15.300 usec
DE 10.00 usec
TE 308.1 K
D1 2.00000000 sec
D11 0.03000000 sec
TD0 1
SFO1 125.7728799 MHz
NUC1 13C
P0 3.33 usec
P1 10.00 usec
PLW1 26.29999924 W
SFO2 500.1320005 MHz
NUC2 1H
CPDPRG[2 waltz65
PCPD2 80.00 usec
PLW2 6.59779978 W
PLW12 0.02577200 W
PLW13 0.01296300 W
F2 - Processing parameters
SI 32768
SF 125.7577885 MHz
WDW EM
SSB 0
LB 1.00 Hz
GB 0
PC 1.40
200
180
160
140
120
100
80
60
40
20
ppm

S33

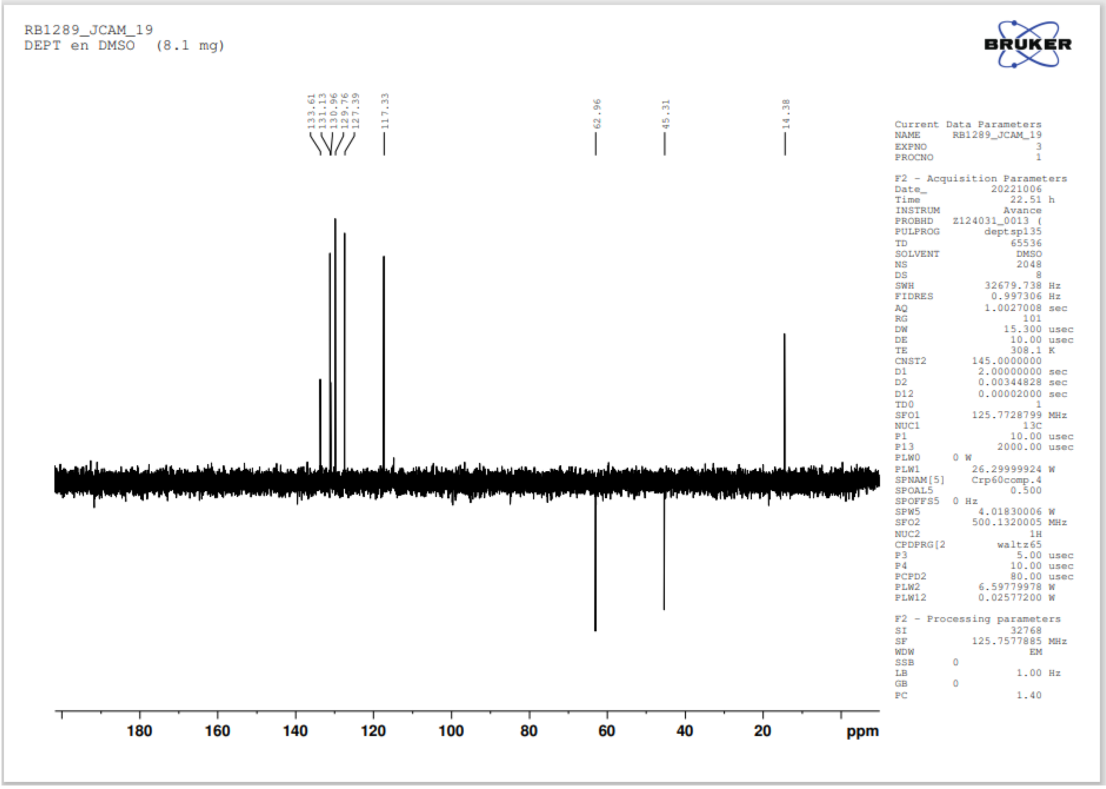
RB1289_JCAM_19
DEPT en DMSO (8.1 mg)
133.61
131.13
130.96
129.76
127.39
117.33
62.96
45.31
14.38
Current Data Parameters
NAME RB1289_JCAM_19
EXPNO 3
PROCNO 1
F2 - Acquisition Parameters
Date_ 20221006
Time 22.51 h
INSTRUM Avance
PROBHD Z124031_0013 (
PULPROG deptsp135
TD 65536
SOLVENT DMSO
NS 2048
DS 8
SWH 32679.738 Hz
FIDRES 0.997306 Hz
AQ 1.0027008 sec
RG 101
DW 15.300 usec
DE 10.00 usec
TE 308.1 K
CNST2 145.0000000
D1 2.00000000 sec
D2 0.00344828 sec
D12 0.00002000 sec
TD0 1
SFO1 125.7728799 MHz
NUC1 13C
P1 10.00 usec
P13 2000.00 usec
PLW0 0 W
PLW1 26.29999924 W
SPNAM[5] Crp60comp.4
SPOAL5 0.500
SPOFFS5 0 Hz
SPW5 4.01830006 W
SFO2 500.1320005 MHz
NUC2 1H
CPDPRG[2 waltz65
P3 5.00 usec
P4 10.00 usec
PCPD2 80.00 usec
PLW2 6.59779978 W
PLW12 0.02577200 W
F2 - Processing parameters
SI 32768
SF 125.7577885 MHz
WDW EM
SSB 0
LB 1.00 Hz
GB 0
PC 1.40
180
160
140
120
100
80
60
40
20
ppm

S34

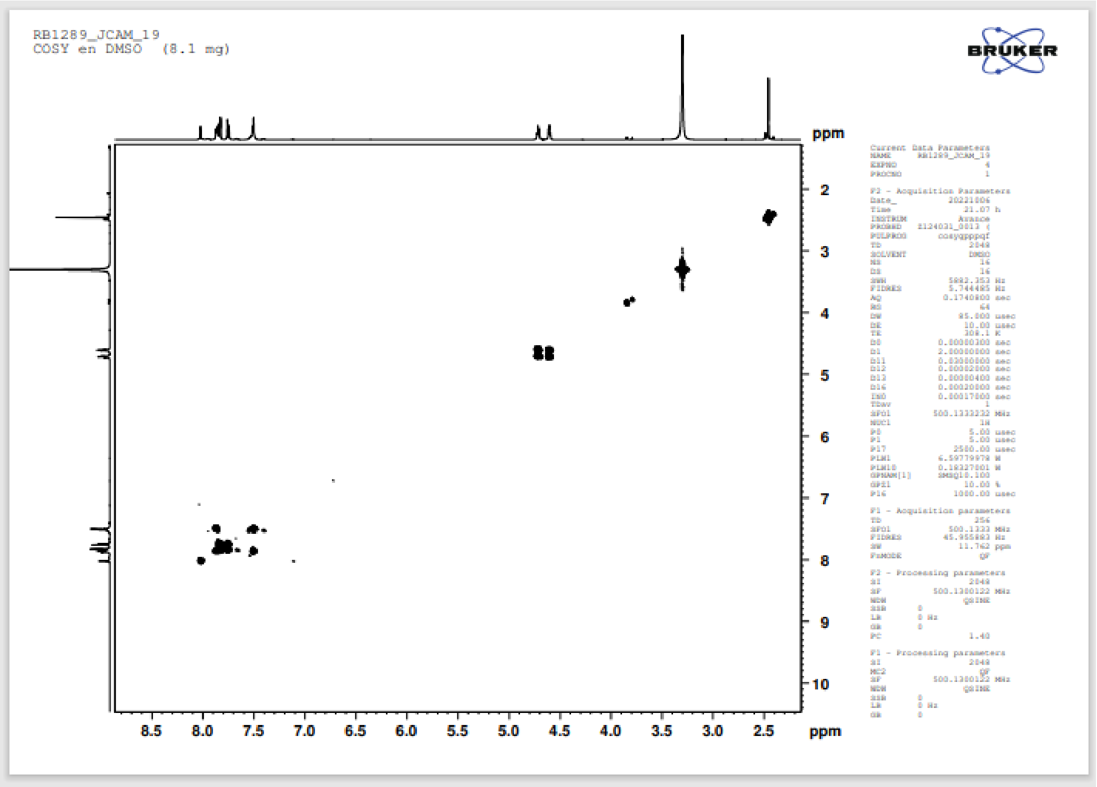
RB1289_JCAM_19
COSY en DMSO (8.1 mg)
BRUKER
ppm
ppm

S35

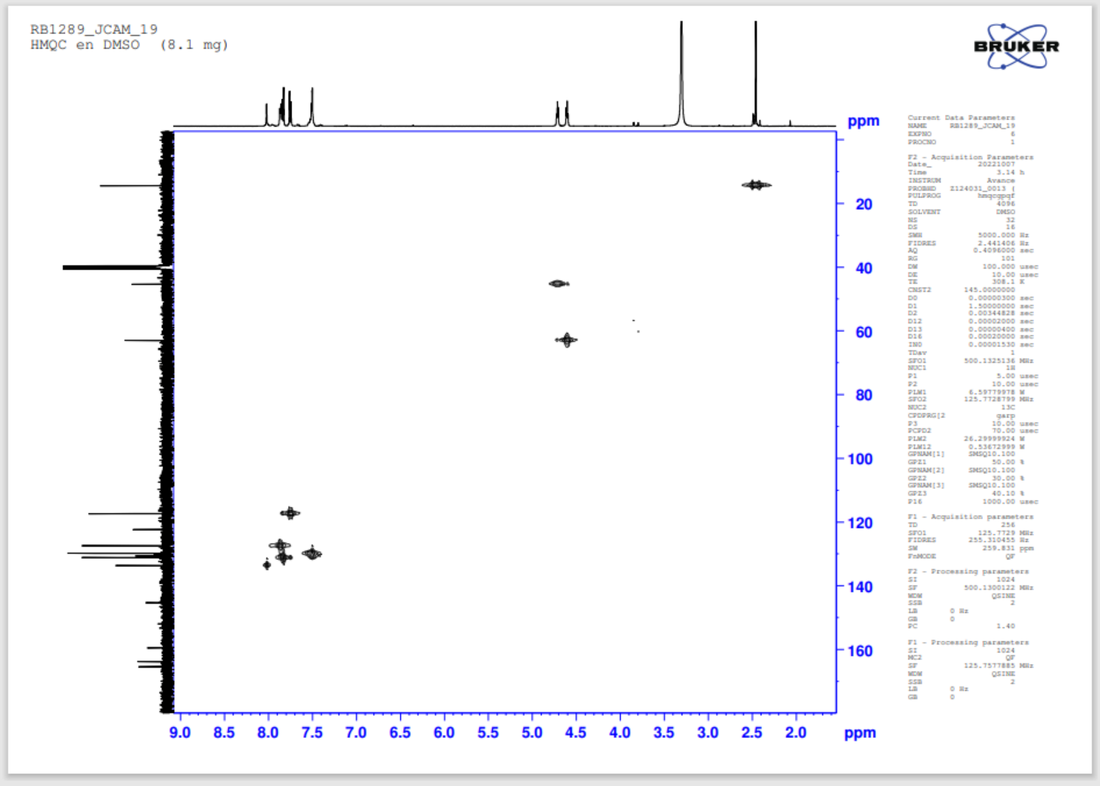
RB1289_JCAM_19
HMQC en DMSO (8.1 mg)
BRUKER
ppm
ppm

S36

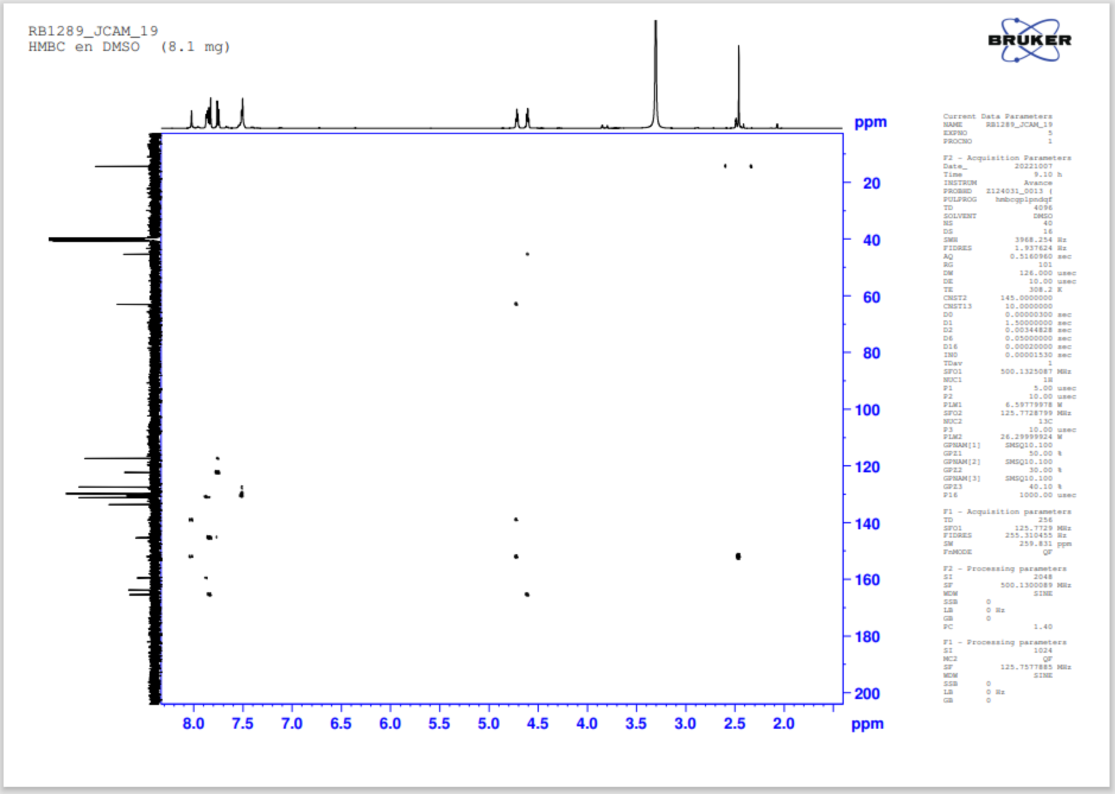
RB1289_JCAM_19
HMBC en DMSO (8.1 mg)
BRUKER
ppm
20
40
60
80
100
120
140
160
180
200
8.0
7.5
7.0
6.5
6.0
5.5
5.0
4.5
4.0
3.5
3.0
2.5
2.0
ppm

S37

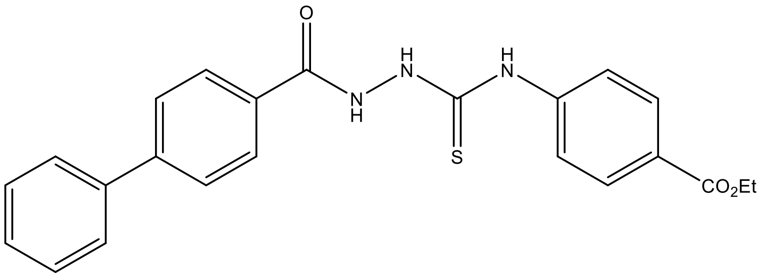
O
H
N
N
H
S
H
N
CO2Et

9

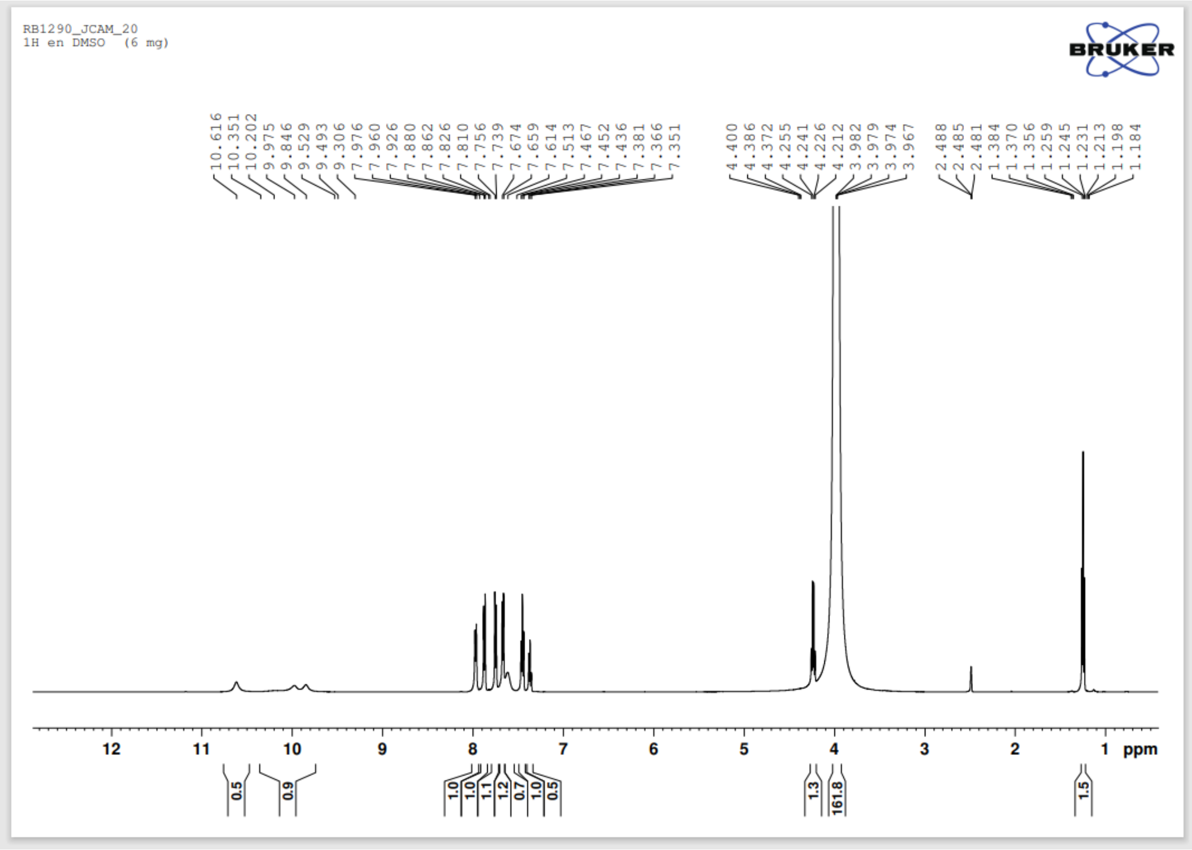
RB1290_JCAM_20
1H en DMSO (6 mg)
BRUKER
10.616
10.351
10.202
9.975
9.846
9.529
9.493
9.306
7.976
7.960
7.926
7.880
7.862
7.826
7.810
7.756
7.739
7.674
7.659
7.614
7.513
7.467
7.452
7.436
7.381
7.366
7.351
4.400
4.386
4.372
4.255
4.241
4.226
4.212
3.982
3.979
3.974
3.967
2.488
2.485
2.481
1.384
1.370
1.356
1.259
1.245
1.231
1.213
1.198
1.184
12
11
10
9
8
7
6
5
4
3
2
1
ppm
0.5
0.9
1.0
1.0
1.1
1.2
0.7
1.0
0.5
1.3
161.8
1.5

S38

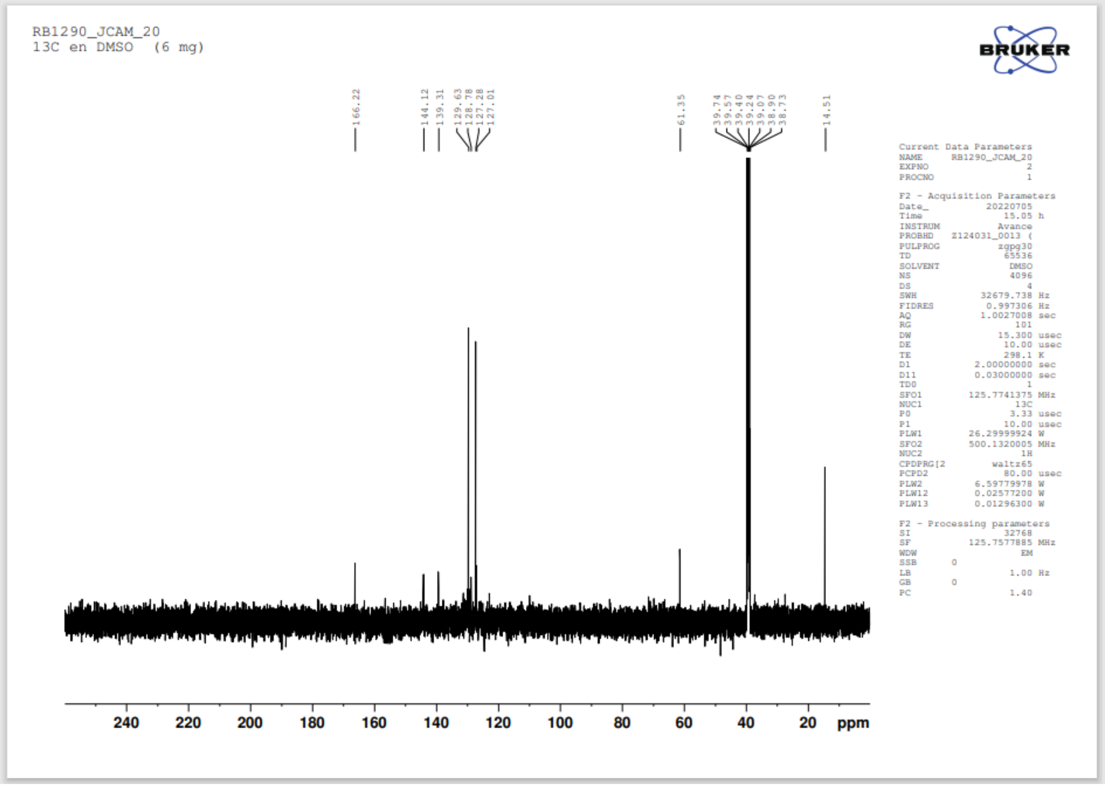

S39

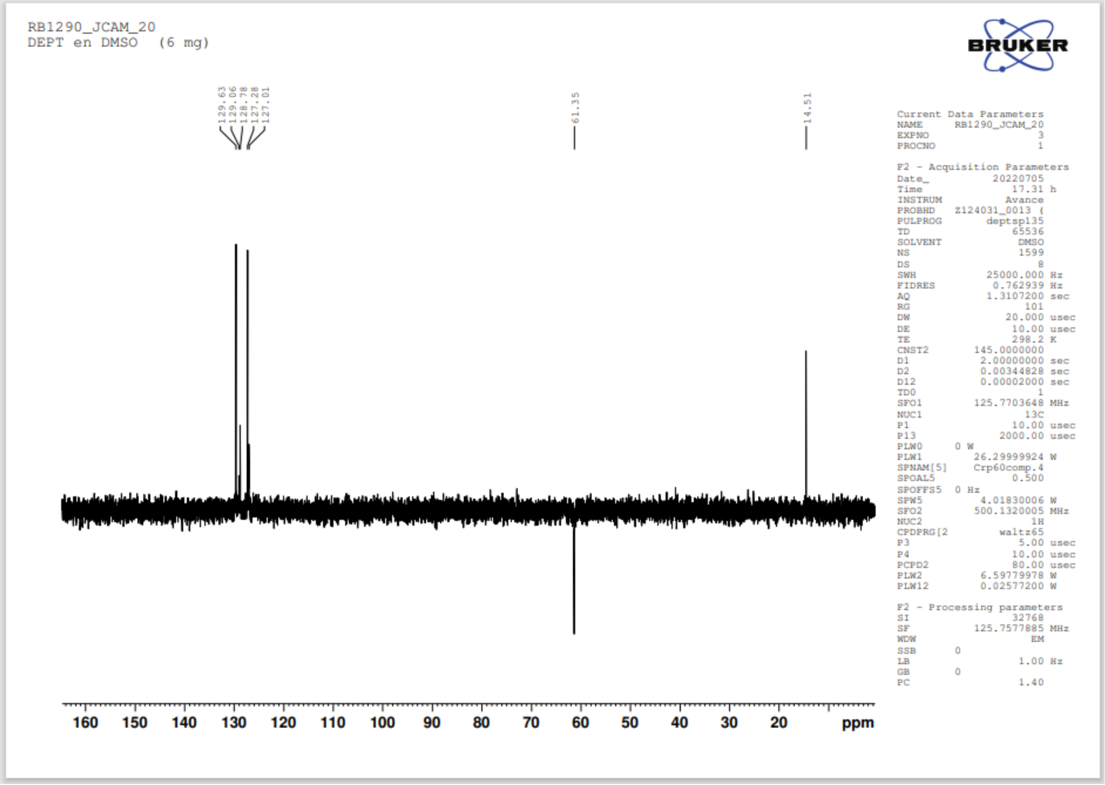

S40

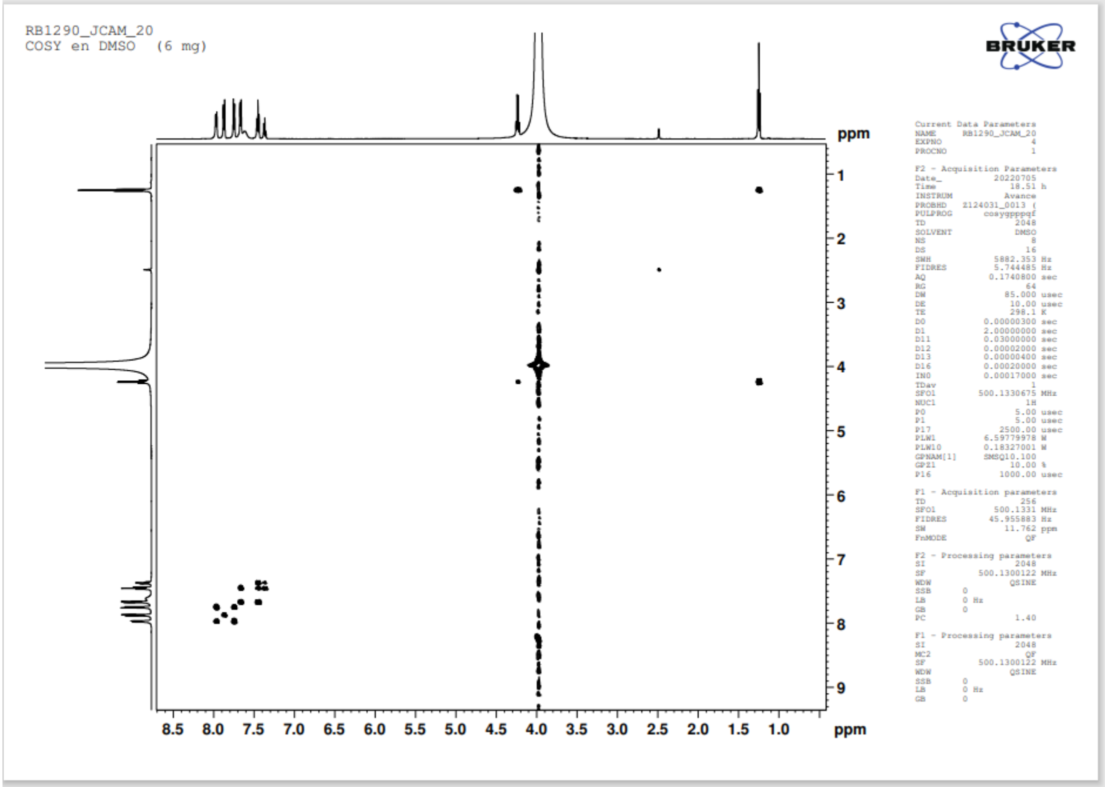
RB1290_JCAM_20
COSY en DMSO (6 mg)
BRUKER
ppm

S41

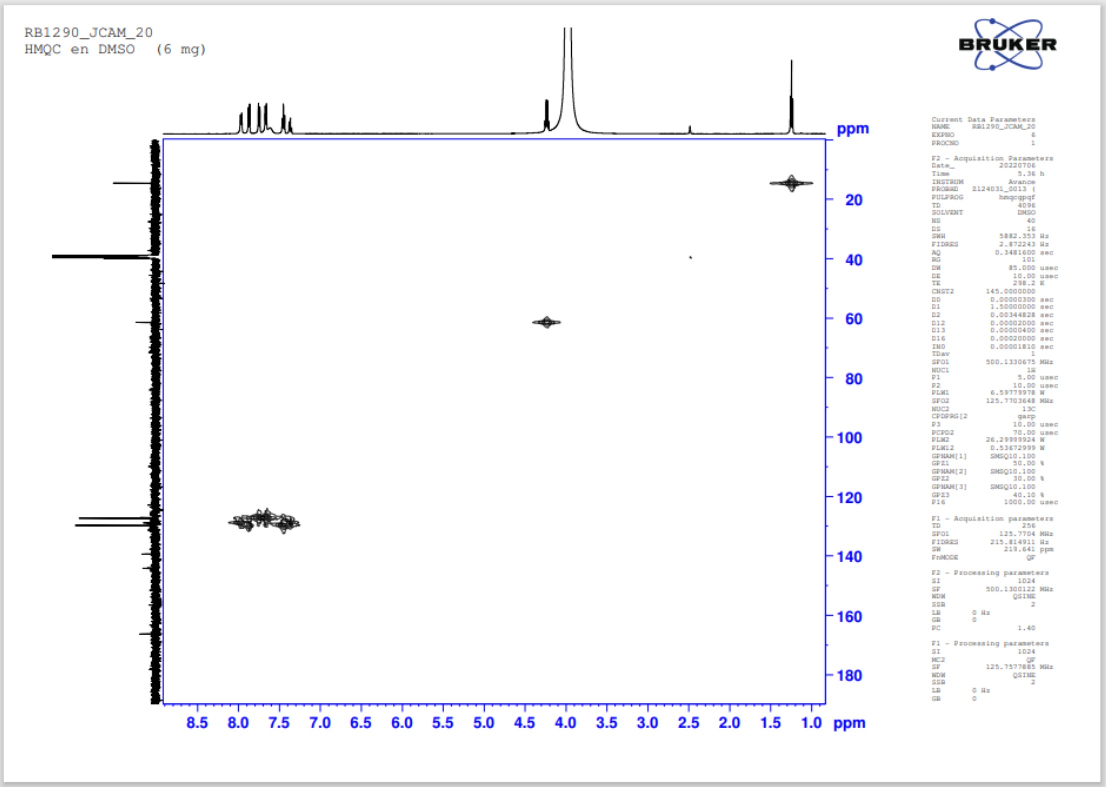
RB1290_JCAM_20
HMQC en DMSO (6 mg)
BRUKER
ppm

S42

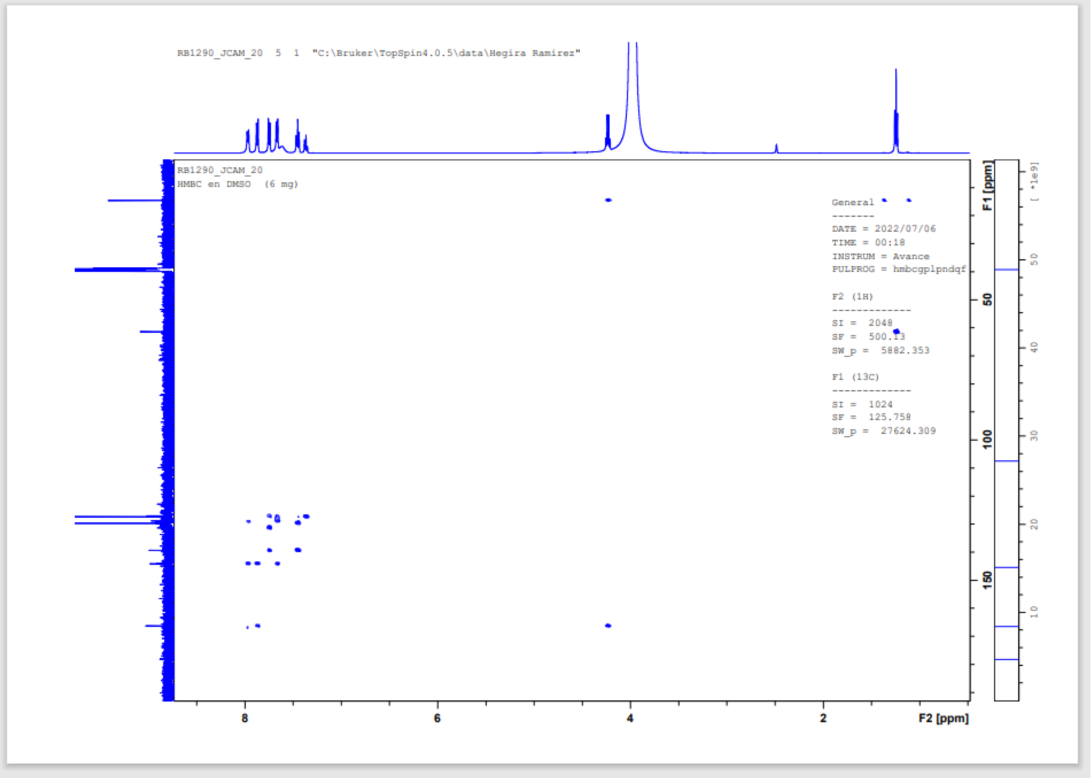

RB1290_JCAM_20 5 1 "C:\Bruker\TopSpin4.0.5\data\Hegira Ramirez"
RB1290_JCAM_20
HMBC en DMSO (6 mg)
General

DATE = 2022/07/06
TIME = 00:18
INSTRUM = Avance
PULPROG = hmbcgplpndqf
F2 (1H)

SI = 2048
SF = 500.13
SW_p = 5882.353
F1 (13C)

SI = 1024
SF = 125.758
SW_p = 27624.309
F1 [ppm]
F2 [ppm]

S43

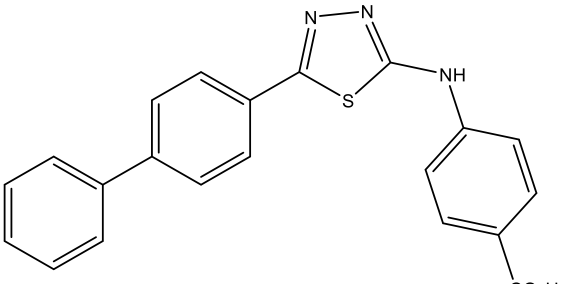
N
N
S
NH
CO2H

15

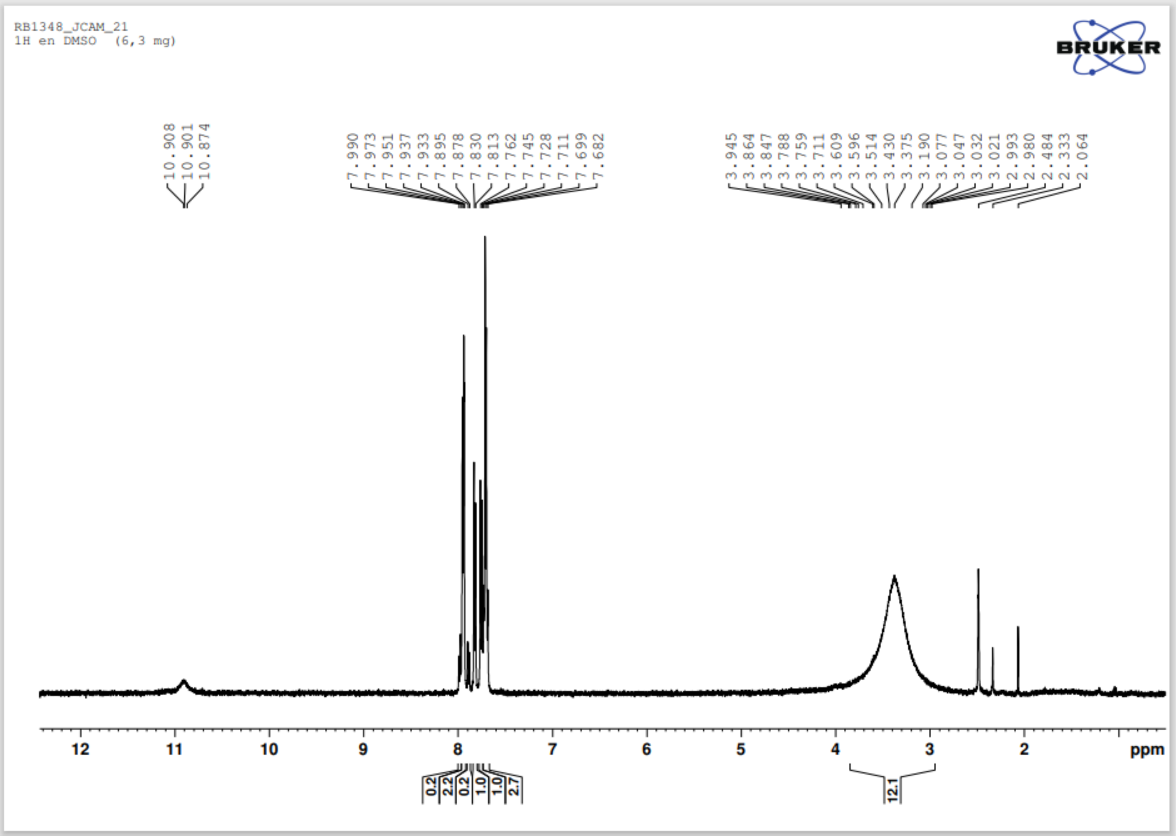
RB1348_JCAM_21
1H en DMSO (6,3 mg)
BRUKER
10.908
10.901
10.874
7.990
7.973
7.951
7.937
7.933
7.895
7.878
7.830
7.813
7.762
7.745
7.728
7.711
7.699
7.682
3.945
3.864
3.847
3.788
3.759
3.711
3.609
3.596
3.514
3.430
3.375
3.190
3.077
3.047
3.032
3.021
2.993
2.980
2.484
2.333
2.064
12
11
10
9
8
7
6
5
4
3
2
ppm
0.2
2.2
0.2
1.0
1.0
2.7
12.1

S44

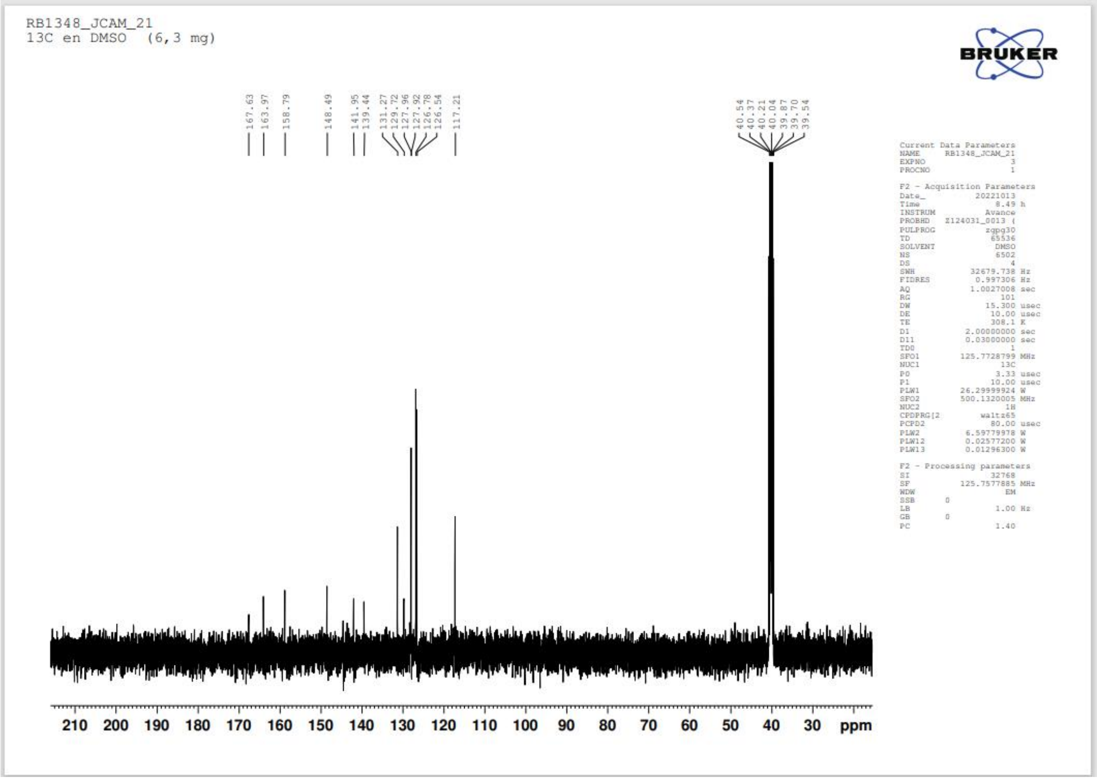
RB1348_JCAM_21
13C en DMSO (6,3 mg)
167.63
163.97
158.79
148.49
141.95
139.44
131.27
129.72
127.96
127.92
126.78
126.54
117.21
40.54
40.37
40.21
40.04
39.87
39.70
39.54
Current Data Parameters
NAME RB1348_JCAM_21
EXPNO 3
PROCNO 1
F2 - Acquisition Parameters
Date_ 20221013
Time 8.49 h
INSTRUM Avance
PROBHD Z124031_0013 (
PULPROG zgpg30
TD 65536
SOLVENT DMSO
NS 6502
DS 4
SWH 32679.738 Hz
FIDRES 0.997306 Hz
AQ 1.0027008 sec
RG 101
DW 15.300 usec
DE 10.00 usec
TE 308.1 K
D1 2.00000000 sec
D11 0.03000000 sec
TD0 1
SFO1 125.7728799 MHz
NUC1 13C
P0 3.33 usec
P1 10.00 usec
PLW1 26.29999924 W
SFO2 500.1320005 MHz
NUC2 1H
CPDPRG[2 waltz65
PCPD2 80.00 usec
PLW2 6.59779978 W
PLW12 0.02577200 W
PLW13 0.01296300 W
F2 - Processing parameters
SI 32768
SF 125.7577885 MHz
WDW EM
SSB 0
LB 1.00 Hz
GB 0
PC 1.40
210 200 190 180 170 160 150 140 130 120 110 100 90 80 70 60 50 40 30 ppm

S45

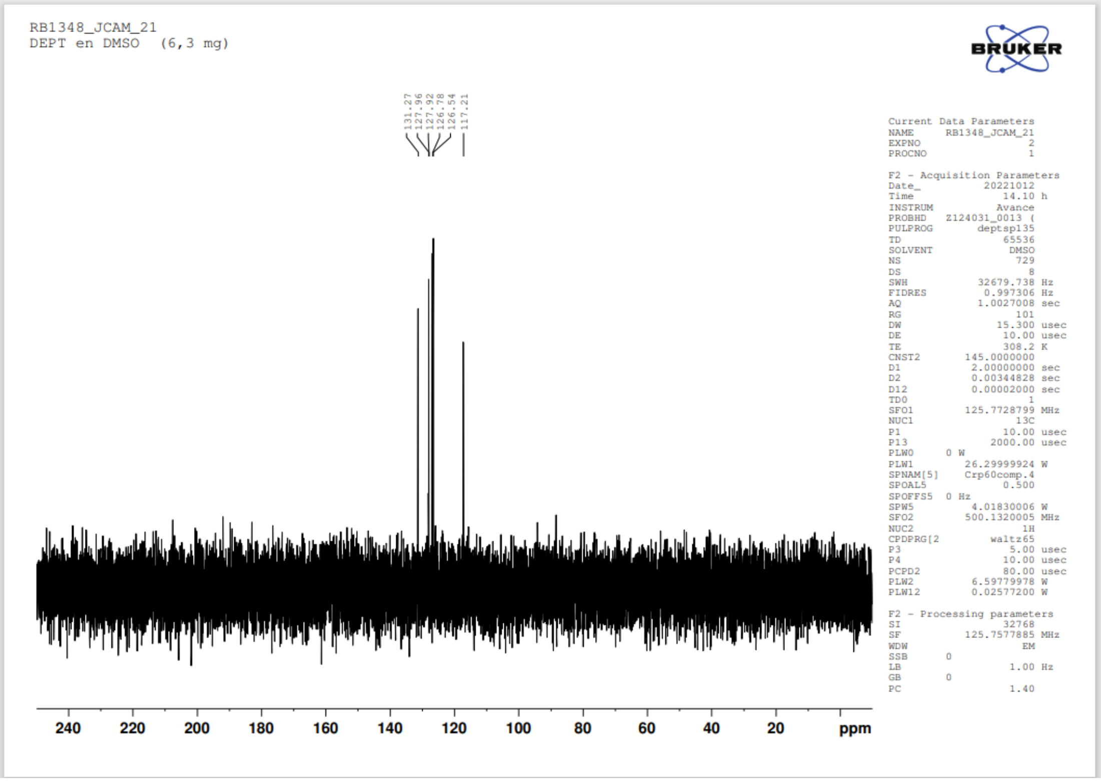
RB1348_JCAM_21
DEPT en DMSO (6,3 mg)
131.27
127.96
127.92
126.78
126.54
117.21
Current Data Parameters
NAME RB1348_JCAM_21
EXPNO 2
PROCNO 1
F2 - Acquisition Parameters
Date_ 20221012
Time 14.10 h
INSTRUM Avance
PROBHD Z124031_0013 (
PULPROG deptsp135
TD 65536
SOLVENT DMSO
NS 729
DS 8
SWH 32679.738 Hz
FIDRES 0.997306 Hz
AQ 1.0027008 sec
RG 101
DW 15.300 usec
DE 10.00 usec
TE 308.2 K
CNST2 145.0000000
D1 2.00000000 sec
D2 0.00344828 sec
D12 0.00002000 sec
TD0 1
SFO1 125.7728799 MHz
NUC1 13C
P1 10.00 usec
P13 2000.00 usec
PLW0 0 W
PLW1 26.29999924 W
SPNAM[5] Crp60comp.4
SPOAL5 0.500
SPOFFS5 0 Hz
SPW5 4.01830006 W
SFO2 500.1320005 MHz
NUC2 1H
CPDPRG[2 waltz65
P3 5.00 usec
P4 10.00 usec
PCPD2 80.00 usec
PLW2 6.59779978 W
PLW12 0.02577200 W
F2 - Processing parameters
SI 32768
SF 125.7577885 MHz
WDW EM
SSB 0
LB 1.00 Hz
GB 0
PC 1.40
240 220 200 180 160 140 120 100 80 60 40 20 ppm

S46

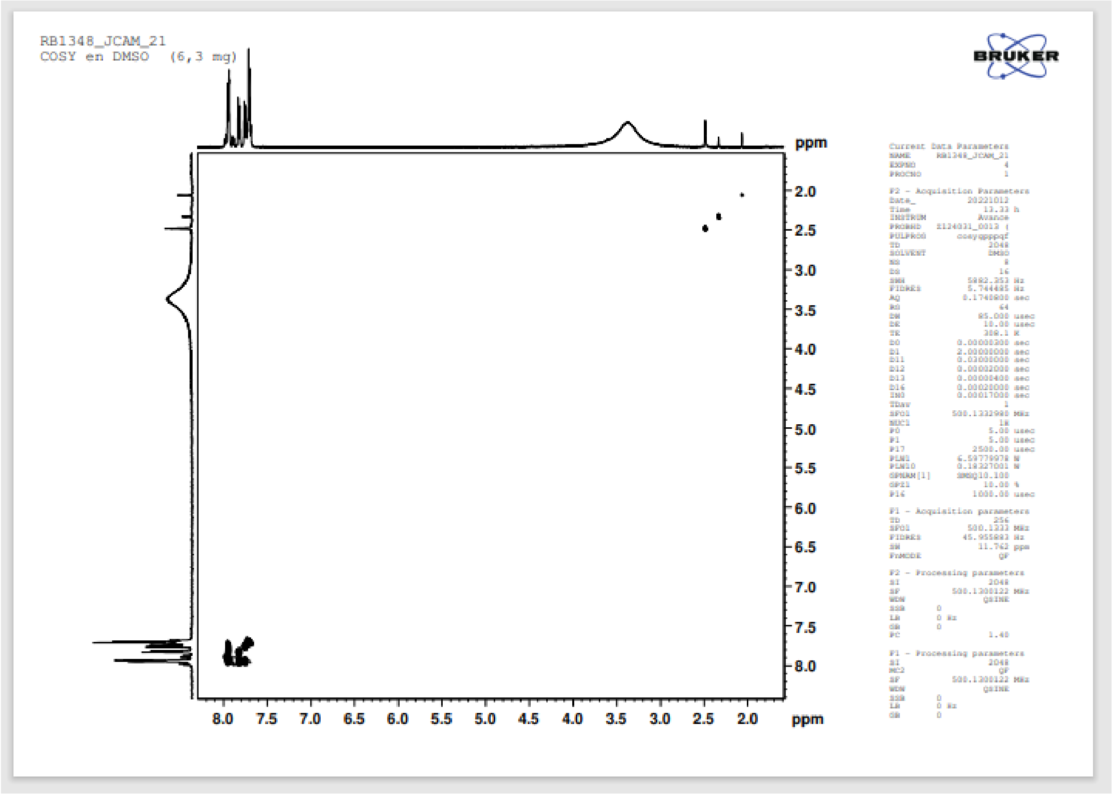
RB1348_JCAM_21
COSY en DMSO (6,3 mg)
BRUKER

S47

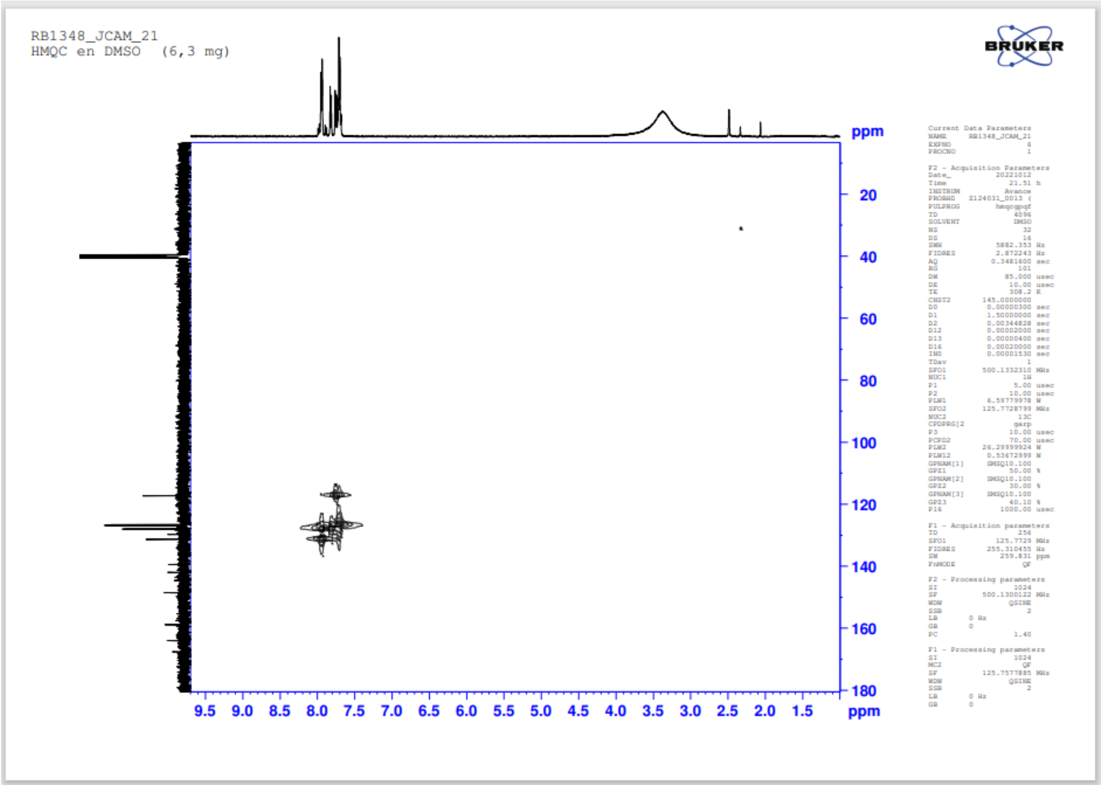
RB1348_JCAM_21
HMQC en DMSO (6,3 mg)
BRUKER

S48

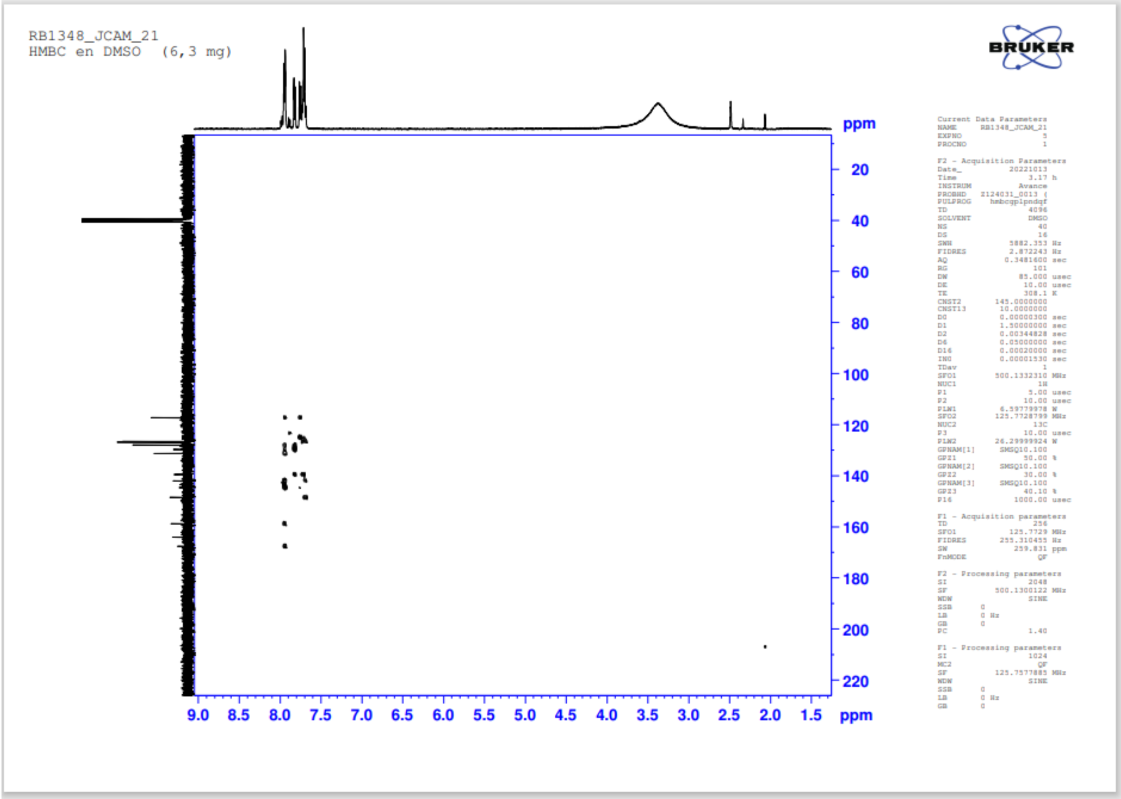
RB1348_JCAM_21
HMBC en DMSO (6,3 mg)
ppm
20
40
60
80
100
120
140
160
180
200
220
9.0 8.5 8.0 7.5 7.0 6.5 6.0 5.5 5.0 4.5 4.0 3.5 3.0 2.5 2.0 1.5 ppm
Current Data Parameters
NAME RB1348_JCAM_21
EXPNO 5
PROCNO 1
F2 - Acquisition Parameters
Date_ 20221013
Time 3.17 h
INSTRUM Avance
PROBHD Z124031_0013 (
PULPROG hmbcgplpndqf
TD 4096
SOLVENT DMSO
NS 40
DS 16
SWH 5882.353 Hz
FIDRES 2.872243 Hz
AQ 0.3481600 sec
RG 101
DW 85.000 usec
DE 10.00 usec
TE 308.1 K
CNST2 145.0000000
CNST13 10.0000000
D0 0.00000300 sec
D1 1.50000000 sec
D2 0.00344828 sec
D6 0.05000000 sec
D16 0.00020000 sec
IN0 0.00001530 sec
TDav 1
SFO1 500.1332310 MHz
NUC1 1H
P1 5.00 usec
P2 10.00 usec
PLW1 6.59779978 W
SFO2 125.7728799 MHz
NUC2 13C
P3 10.00 usec
PLW2 26.29999924 W
GPNAM[1] SMSQ10.100
GPZ1 50.00 %
GPNAM[2] SMSQ10.100
GPZ2 30.00 %
GPNAM[3] SMSQ10.100
GPZ3 40.10 %
P16 1000.00 usec
F1 - Acquisition parameters
TD 256
SFO1 125.7729 MHz
FIDRES 255.310455 Hz
SW 259.831 ppm
FnMODE QF
F2 - Processing parameters
SI 2048
SF 500.1300122 MHz
WDW SINE
SSB 0
LB 0 Hz
GB 0
PC 1.40
F1 - Processing parameters
SI 1024
MC2 QF
SF 125.7577885 MHz
WDW SINE
SSB 0
LB 0 Hz
GB 0

S49

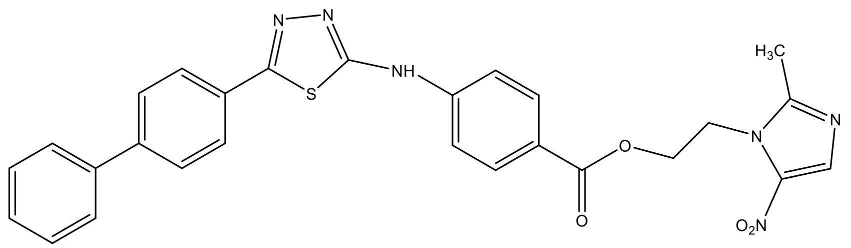

N
N
S
NH
O
O
H_3C
N
N
O_2N

22

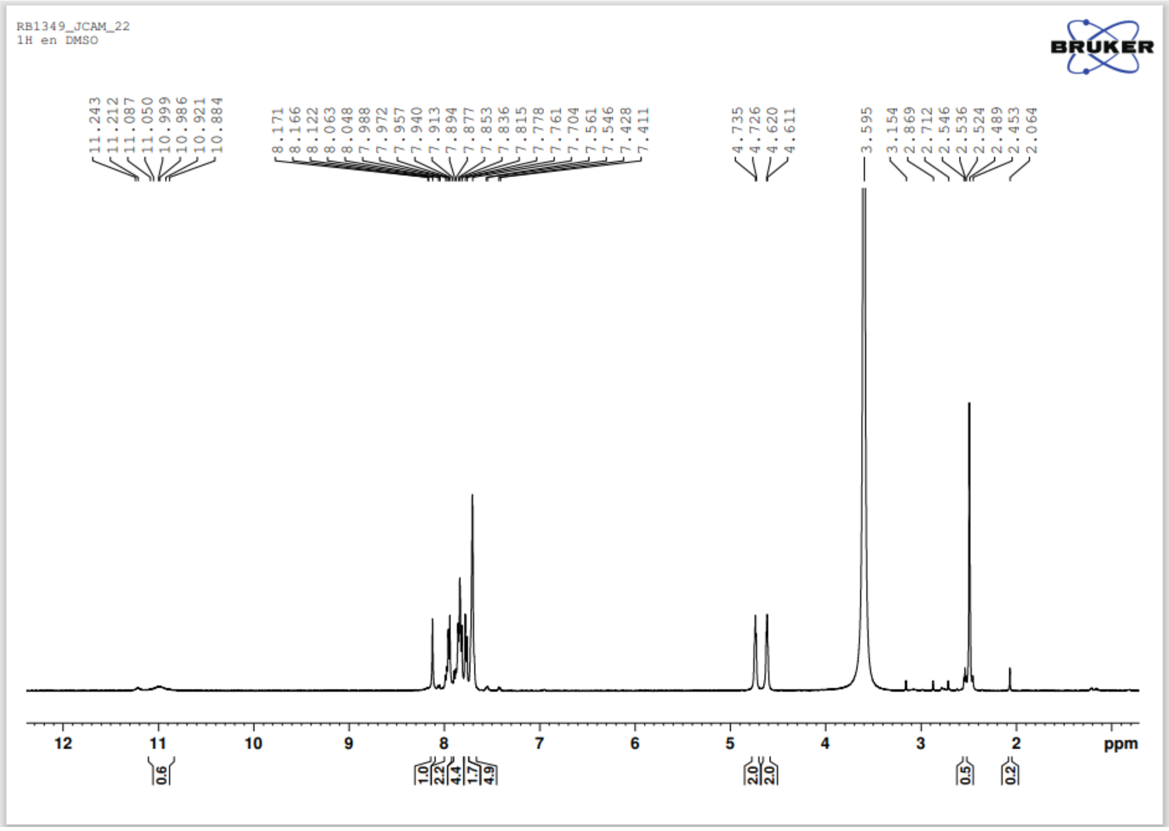

RB1349_JCAM_22
1H en DMSO
BRUKER
11.243
11.212
11.087
11.050
10.999
10.986
10.921
10.884
8.171
8.166
8.122
8.063
8.048
7.988
7.972
7.957
7.940
7.913
7.894
7.877
7.853
7.836
7.815
7.778
7.761
7.704
7.561
7.546
7.428
7.411
4.735
4.726
4.620
4.611
3.595
3.154
2.869
2.712
2.546
2.536
2.524
2.489
2.453
2.064
12
11
10
9
8
7
6
5
4
3
2
ppm
0.6
1.0
2.2
2.2
4.4
1.7
4.9
2.0
2.0
0.5
0.2

S50

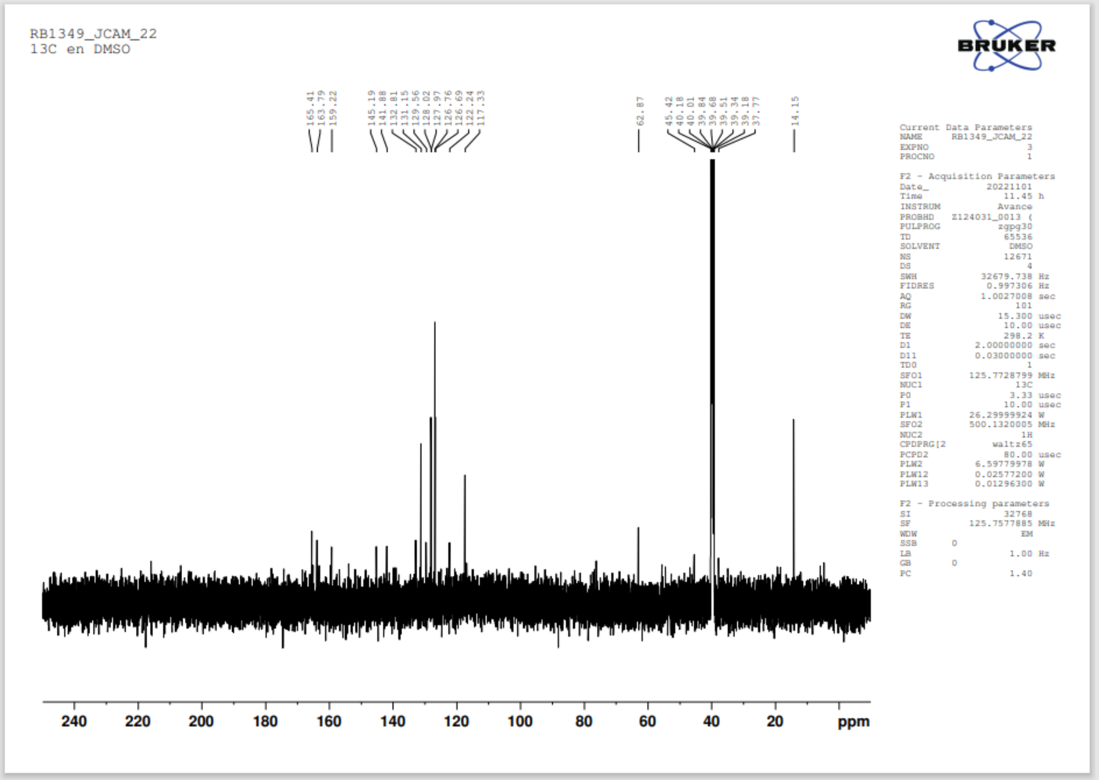
RB1349_JCAM_22
13C en DMSO
165.41
163.79
159.22
145.19
141.88
132.81
131.15
129.56
128.02
127.97
126.76
126.69
122.24
117.33
62.87
45.42
40.18
40.01
39.84
39.68
39.51
39.34
39.18
37.77
14.15
240 220 200 180 160 140 120 100 80 60 40 20 ppm
Current Data Parameters
NAME RB1349_JCAM_22
EXPNO 3
PROCNO 1
F2 - Acquisition Parameters
Date_ 20221101
Time 11.45 h
INSTRUM Avance
PROBHD Z124031_0013 (
PULPROG zgpg30
TD 65536
SOLVENT DMSO
NS 12671
DS 4
SWH 32679.738 Hz
FIDRES 0.997306 Hz
AQ 1.0027008 sec
RG 101
DW 15.300 usec
DE 10.00 usec
TE 298.2 K
D1 2.00000000 sec
D11 0.03000000 sec
TD0 1
SFO1 125.7728799 MHz
NUC1 13C
P0 3.33 usec
P1 10.00 usec
PLW1 26.29999924 W
SFO2 500.1320005 MHz
NUC2 1H
CPDPRG[2 waltz65
PCPD2 80.00 usec
PLW2 6.59779978 W
PLW12 0.02577200 W
PLW13 0.01296300 W
F2 - Processing parameters
SI 32768
SF 125.7577885 MHz
WDW EM
SSB 0
LB 1.00 Hz
GB 0
PC 1.40

S51

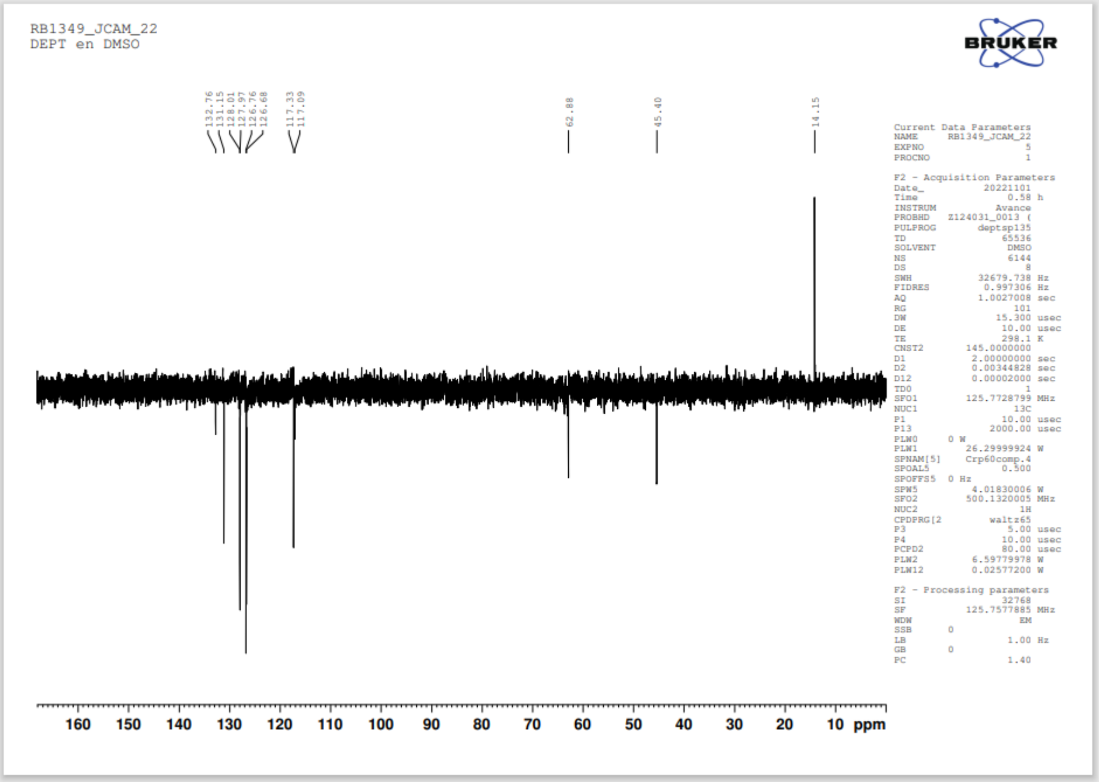
RB1349_JCAM_22
DEPT en DMSO
132.76
131.15
128.01
127.97
126.76
126.68
117.33
117.09
62.88
45.40
14.15
160 150 140 130 120 110 100 90 80 70 60 50 40 30 20 10 ppm
Current Data Parameters
NAME RB1349_JCAM_22
EXPNO 5
PROCNO 1
F2 - Acquisition Parameters
Date_ 20221101
Time 0.58 h
INSTRUM Avance
PROBHD Z124031_0013 (
PULPROG deptsp135
TD 65536
SOLVENT DMSO
NS 6144
DS 8
SWH 32679.738 Hz
FIDRES 0.997306 Hz
AQ 1.0027008 sec
RG 101
DW 15.300 usec
DE 10.00 usec
TE 298.1 K
CNST2 145.0000000
D1 2.00000000 sec
D2 0.00344828 sec
D12 0.00002000 sec
TD0 1
SFO1 125.7728799 MHz
NUC1 13C
P1 10.00 usec
P13 2000.00 usec
PLW0 0 W
PLW1 26.29999924 W
SPNAM[5] Crp60comp.4
SPOAL5 0.500
SPOFFS5 0 Hz
SPW5 4.01830006 W
SFO2 500.1320005 MHz
NUC2 1H
CPDPRG[2 waltz65
P3 5.00 usec
P4 10.00 usec
PCPD2 80.00 usec
PLW2 6.59779978 W
PLW12 0.02577200 W
F2 - Processing parameters
SI 32768
SF 125.7577885 MHz
WDW EM
SSB 0
LB 1.00 Hz
GB 0
PC 1.40

S52

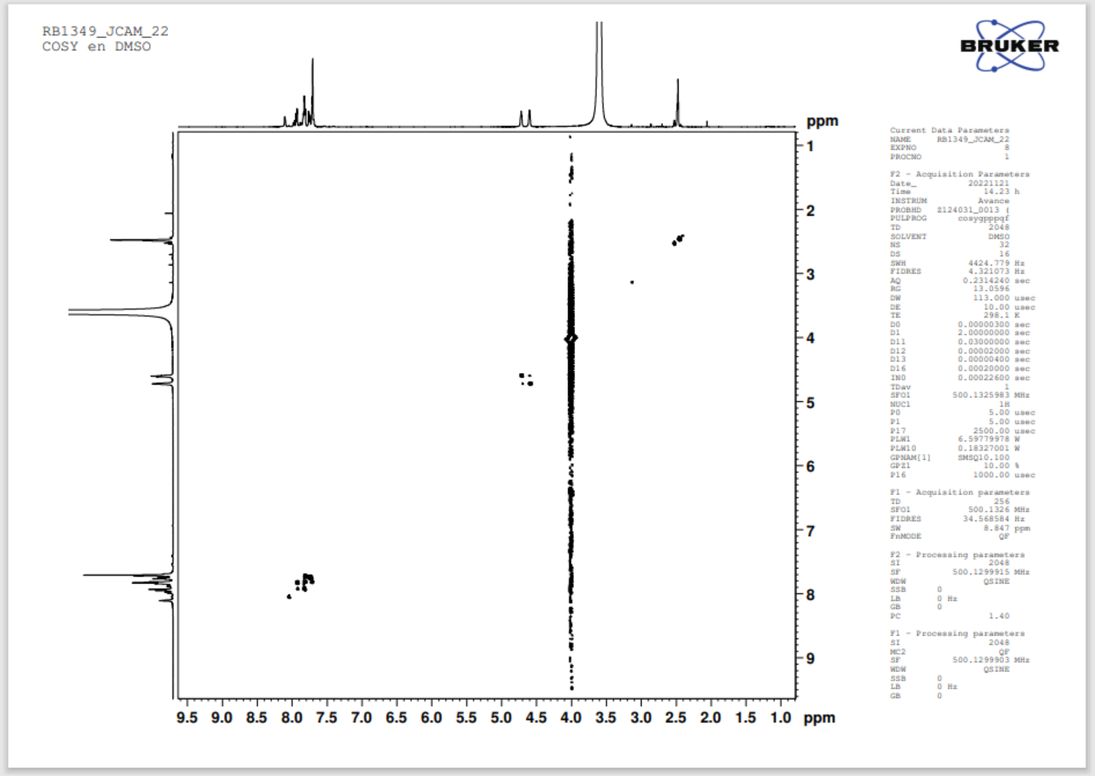
RB1349_JCAM_22
COSY en DMSO
BRUKER
ppm
9.5 9.0 8.5 8.0 7.5 7.0 6.5 6.0 5.5 5.0 4.5 4.0 3.5 3.0 2.5 2.0 1.5 1.0 ppm

S53

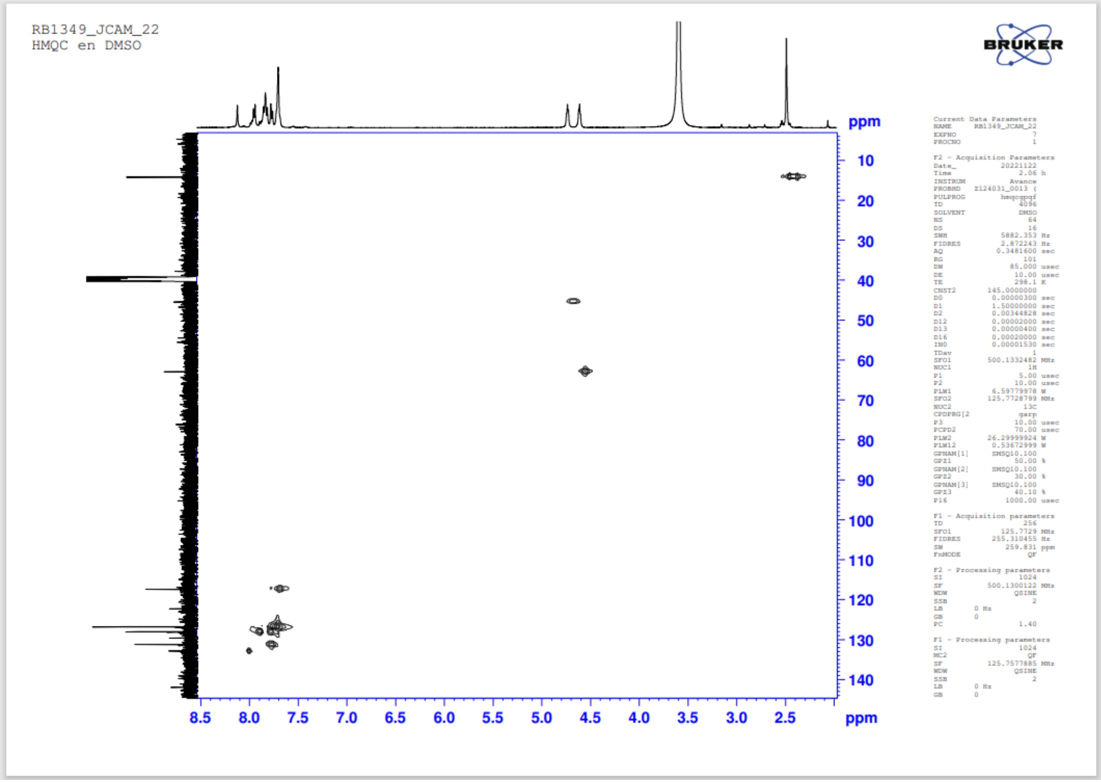
RB1349_JCAM_22
HMQC en DMSO
BRUKER
ppm
8.5 8.0 7.5 7.0 6.5 6.0 5.5 5.0 4.5 4.0 3.5 3.0 2.5 ppm

S54

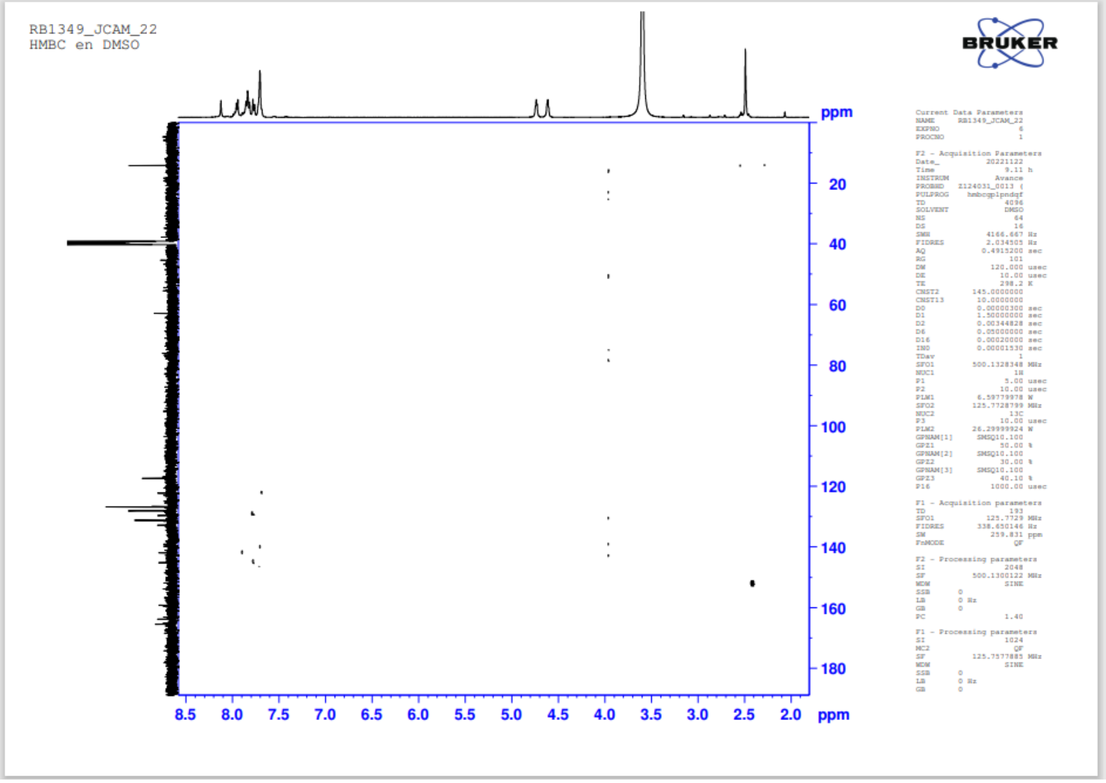
RB1349_JCAM_22
HMBC en DMSO
BRUKER
ppm
20
40
60
80
100
120
140
160
180
8.5
8.0
7.5
7.0
6.5
6.0
5.5
5.0
4.5
4.0
3.5
3.0
2.5
2.0
ppm
